# Bioinformatics analysis of lncRNA-related ceRNA networks in the peripheral blood lymphocytes of Kazakh patients with essential hypertension in Xinjiang

**DOI:** 10.3389/fcvm.2023.1155767

**Published:** 2023-06-15

**Authors:** Yan Wang, Jie Gao, Liang Zhang, Rui Yang, Yingying Zhang, Liya Shan, Xinzhi Li, Ketao Ma

**Affiliations:** ^1^ Key Laboratory of Xinjiang Endemic and Ethnic Diseases, Ministry of Education, Shihezi University School of Medicine, Shihezi, China; ^2^ NHC Key Laboratory of Prevention and Treatment of Central Asia High Incidence Diseases, First Affiliated Hospital, Shihezi University School of Medicine, Shihezi, China; ^3^ Department of Physiology, Shihezi University School of Medicine, Shihezi, China; ^4^ Department of Pathophysiology, Shihezi University School of Medicine, Shihezi, China

**Keywords:** bioinformatics analysis, ceRNA, essential hypertension, Kazakh, lncRNA

## Abstract

**Objective:**

Here, we aimed to investigate long non-coding RNA (lncRNA) expression characteristics in the peripheral blood lymphocytes of Xinjiang Kazakh people with essential hypertension and the underlying regulatory mechanisms of competing endogenous RNAs (ceRNA).

**Methods:**

From April 2016 to May 2019, six Kazakh patients with essential hypertension and six Kazakh healthy participants were randomly selected from the inpatient and outpatient cardiology departments of the First Affiliated Hospital of Shihezi University Medical College, Xinjiang. After detecting the expression levels of lncRNA and mRNA in the peripheral blood lymphocytes using gene chip technology, their levels in the hypertensive group were compared with those in the control group. Six differentially expressed lncRNAs were randomly selected for real-time PCR to verify the accuracy and reliability of the gene chip results. GO functional clustering and KEGG pathway analyses were performed for differentially expressed genes. The ceRNA regulatory network of lncRNA-miRNA-mRNA was constructed, followed by visualization of the results. The expressions of miR-139-5p and DCBLD2 after PVT1 overexpression in 293T cells were detected by qRT-PCR and Western blot.

**Results:**

In the test group, 396 and 511 differentially expressed lncRNAs and mRNAs, respectively, were screened out. The trend of real-time PCR results was consistent with that of the microarray results. The differentially expressed mRNAs were found to be primarily involved in the adhesion spot, leukocyte migration via endothelial cells, gap junction, actin cytoskeleton regulation, and extracellular matrix-receptor interaction signaling pathways. By constructing the ceRNA regulatory network, we found that lncRNA PVT1-miR-139-5p-DCBLD2 has a potential ceRNA regulatory mechanism involved in the development of essential hypertension in Xinjiang Kazakh people. In 293T cells, lncRNA PVT1 overexpression inhibited miR-139-5p and DCBLD2 levels.

**Conclusions:**

Our findings indicate that differentially expressed lncRNAs may be involved in the development of essential hypertension. lncRNA PVT1-miR-139-5p-DCBLD2 was indicated to comprise a potential ceRNA regulatory mechanism involved in the development of essential hypertension in the Xinjiang Kazakh population. Thus, it may act as a novel screening marker or therapeutic target for essential hypertension in this population.

## 1. Introduction

Hypertension, a syndrome characterized by the increasing diastolic and systolic blood pressures of the systemic circulation, is accompanied by various cardio-cerebrovascular diseases. It is the most common chronic cardiovascular disease in China. Essential hypertension accounts for 95% of all hypertension cases. According to the Fifth National Hypertension Control Survey released by the National Center for Cardiovascular Diseases in 2018, the prevalence of essential hypertension in China has risen to 23.2%. In the next 5–10 years, the average number of people with hypertension is predicted to rise to 1.1 billion globally (1). Essential hypertension is one of the leading causes of chronic renal failure and an independent risk factor for highly fatal cardiovascular diseases such as stroke, myocardial infarction, heart failure, and aneurysm (2). As a complex polygenic chronic hereditary disease formed by the alternation of environmental and genetic factors, the pathogenesis of essential hypertension is relatively complex. Here, the classic mechanisms include the vascular system, sympathetic and parasympathetic nervous system, renin–angiotensin–aldosterone system disorder, endothelin system disorder, and insulin resistance mechanisms (3, 4). In recent years, increasing studies have shown that various immune cell subsets infiltrate the blood vessels, kidneys, hearts, and other tissues or organs during hypertension. Among these immune cell subsets, lymphocyte subsets play a key role in the development of hypertension and end-organ damage (5–7). Th1 cell-mediated immune responses promote angiotensin (ANG) II-induced increases in blood pressure, vascular inflammation, and vascular dysfunction (8). Tregs can prevent ANG II hypertension-induced cerebrovascular damage by regulating brain and peripheral inflammation (9). Dingwell et al. found that B-cell deficiency can reduce blood pressure in mice and that B-cells regulate water and sodium balance through renal vasopressin receptors, thereby affecting blood pressure (10). Lymphocytes are closely involved in the pathogenesis and evolution of essential hypertension. Investigating new biomarkers of essential hypertension and elucidating the underlying molecular mechanisms of its occurrence are necessary to formulate informed strategies for hypertension prevention, control, and treatment.

In China, there are distinct geographical differences in the prevalence of hypertension, with more people suffering from hypertension in the north than in the south, and the prevalence of hypertension varies among different ethnic groups and races (11). In 2017, a survey on hypertension among the Han, Kazakh, and Uyghur ethnic groups showed (12) that the prevalence of hypertension in over 35-year-old adults in Xinjiang was 35.01%, whereas that in Kazakh was 52.57%. The risk of hypertension in the older population is 0.989 times that of normal people (13–15), which was much higher than the national level. On comparing the incidence rate of Kazakh hypertension in 56 ethnic groups in China (16), the prevalence rate is found to be high, not only an early onset age, family tend to be very clear, and blood pressure level is higher; however, the awareness and control for treating high blood pressure are lower than other ethnic and national levels (17). The proliferation of T lymphocytes and the release of inflammatory factors can promote the occurrence and development of hypertension in Kazakh hypertension patients in Xinjiang (18). Therefore, investigating the underlying mechanism of adaptive immune response regulation in hypertension in the Xinjiang Kazakh population is of great significance.

Gene-specific expression differences determine the pathological progression of individual diseases. Non-coding region genes account for 98.2% of the total number of human genes. Long non-coding RNAs (lncRNAs) play a key role in the regulation of gene expression and participate in the pathological process of the body (19). lncRNAs exhibit various molecular functions and are involved in several processes, such as dose compensation effect, epigenetic regulation, cell cycle regulation, and cell differentiation regulation. The function of lncRNAs predominantly depends on their secondary structure. lncRNAs can bind to proteins, cause chromatin remodeling, and affect the function of transcription factors; moreover, they can directly or indirectly bind to mRNA, thereby affecting its translation, cleavage, and degradation processes (20, 21). lncRNA TUG1 is highly expressed in the aorta of spontaneously hypertensive rats (SHRs). lncRNA TUG1/miR-145-5p/FGF10 promotes the proliferation and migration of vascular smooth muscle cells in the hypertensive state by activating the Wnt/β-catenin pathway. lncRNA MHRT can prevent stress-induced cardiac remodeling, while lncRNA FENDRR regulates cardiac wall development by controlling epigenetic patterns of cardiac transcription factor promoters. As a non-coding RNA associated with cardiac mesoderm enhancer, lncRNA CARMEN plays a key role in the differentiation of cardiac precursor cells (22). lncRNA is involved in the pathogenesis of hypertension. Nevertheless, the expression profile and regulatory network of lncRNA in Xinjiang Kazakh residents and Kazakh patients with essential hypertension in the same region have not yet been reported. Therefore, in this study, we aimed to elucidate the underlying molecular mechanism of hypertension. To this end, our objective was to screen the differentially expressed lncRNAs in Xinjiang Kazakh patients with essential hypertension and Kazakh healthy subjects using lncRNA microarray technology and analyze the gene regulatory network of the corresponding lncRNAs. We believe that the findings of our study will aid in providing a novel basis for the identification of early diagnostic biomarkers of hypertension.

## 2. Materials and methods

### 2.1. Research object

From April 2016 to May 2019, six Kazakh patients with essential hypertension and six Kazakh healthy subjects were randomly selected from the inpatient and outpatient cardiology departments of the First Affiliated Hospital of Shihezi University School of Medicine, Xinjiang.

According to the diagnostic criteria of the Chinese Guidelines for the Prevention and Treatment of Hypertension 2018, hypertension is defined as the measurement of blood pressure thrice on different days without the use of antihypertensive drugs, with systolic blood pressure (SBP) ≥140 (mmHg) and/or diastolic blood pressure (DBP) ≥90 (mmHg).

Inclusive criteria for this study were as follows: ≥38-year-old patients who were initially diagnosed with essential hypertension were included in the hypertension group. Kazakh healthy people with no history of hypertension and antihypertensive drug treatment, average systolic blood pressure values <140 mmHg, and average diastolic blood pressure values <90 mmHg, measured as per the aforementioned guidelines, were categorized as the control group.

Exclusion criteria for this study were as follows: Patients with secondary hypertension complicated with malignant tumors, heart/renal failure, stroke, and autoimmune system, among other diseases. All subjects signed informed consent, and the study was approved by the ethics committee of the First Affiliated Hospital of Shihezi University (Ethical review approval No.: 2018-025-01).

### 2.2. Data collection

#### 2.2.1. Information collection

The following information on the Kazakh patients with essential hypertension and Kazakh healthy people was obtained: Gender, age, SBP, DBP, total cholesterol (TC), triglyceride (TG), high-density lipoprotein cholesterol (HDL-C), low-density lipoprotein (LDL-C), serum creatinine (Scr), and alanine transaminase (ALT) levels.

#### 2.2.2. Blood sample collection

Peripheral venous blood (5 ml) was collected into EDTA anticoagulant blood collection tubes. The number of subjects was marked on the tube wall, and the anticoagulant blood vessels were mixed upside down. Peripheral blood lymphocytes were isolated by using Ficoll-Hypaque density gradient centrifugation, and Trizol reagent was added to the isolated peripheral blood lymphocytes, which were then stored in a refrigerator at −80°C for further analysis.

#### 2.2.3. RNA isolation

Total RNA in cells was extracted using the Trizol one-step method, and the amount and quality of RNA were evaluated using NanoDropTM 2000 (Thermo Fisher Scientific, Waltham, MA, USA). RNA integrity was evaluated using an Agilent2100 bioanalyzer (Agilent Technologies Inc., Palo Alto, California, USA). Total RNA was subsequently purified using RNeasy Micro Kit (Thermo Fisher Scientific, Waltham, MA, USA) and RNase-Free DNase Set (Thermo Fisher Scientific, Waltham, MA, USA).

### 2.3. Bioinformatics analysis of chip data

In this study, CapitalBio Technology Human LncRNA Array v4 chip was used to detect the gene expression profile of human species, and the lncRNA and mRNA expression profiles in peripheral blood lymphocytes were determined by Beijing Boao Jingdian Biotechnology Co., Ltd. Here, 12 microarrays were prepared using 12 specimens, and the samples were labeled with the Jingxin® Biochip Universal Labeling Kit. The chip scans were processed using Feature Extraction software to obtain the original data files; these files were imported into the GeneSpring software for chip data homogenization and difference analysis. Cluster 3.0 software was used for cluster analysis and graphical presentation. When the fold change (FC) of lncRNA or mRNA expression between the two groups was >1.5 and the *P*-value was <0.05, it was defined as a differential gene. The mRNA-lncRNA co-expression analysis was performed according to the gene expression correlation. Pearson correlation coefficient was applied to measure the correlation between lncRNA and the target genes. We then calculated the Pearson correlation coefficient matrix and performed a correlation analysis. Values of |*r*| > 0.9 and *P* < 0.05 were set as the threshold for subsequent analysis. The differentially expressed lncRNAs targeting mRNA were analyzed for the KEGG pathway and GO function enrichment.

### 2.4. ceRNA network analysis and construction

According to the selected differentially expressed lncRNAs, miRNAs interacting with them were matched using the miRcode prediction database, and target genes were screened using the miRNA target gene prediction databases (miRDB, miRTarBase, and TargetScan). Based on the selected lncRNAs, miRNAs, and mRNAs, Cytoscape architecture was used to draw the ceRNA regulatory network.

### 2.5. Cell culture and transfection

The human embryonic kidney cell line 293T used in this study was purchased from the Cell Bank of Chinese Academy of Sciences (Shanghai, China). The cells were cultured in DMEM medium (Gibco, Thermo Fisher Scientific, Inc.), in which 10% fetal bovine serum (Biological Industries), 100 U/ml penicillin and 100 µ g/mL streptomycin (Invitrogen, CA, USA) were added, and then maintained in an incubator at 37°C with 5% CO2. The overexpression plasmid of PVT1 (vector as control) was obtained from GenePharma (Shanghai, China). 293T cells cultured to 70%–80% confluence were transfected with Lipofectamine 2000 (Invitrogen, CA, USA).

### 2.6. Western blot analysis

After the cell intervention, whole-cell protein lysate was collected and ultrasonically broken, centrifuged at 12,000 rpm at 4°C for 10 min, and protein supernatant was collected. BCA protein assay kit was used to determine the total protein concentration, and then 1 × loading buffer was added and boiled for 5 min. During electrophoresis, protein in each group of samples was added according to the total amount of 30 µg per well. After electrophoresis, the protein in the gel was transferred to the PVDF membrane in the sequence of sponge—filter paper—gel—PVDF membrane—filter paper—sponge. After electrolysis, the membrane was closed with 5% BSA solution for non-specific binding sites. The antibody was diluted with TBST solution containing 1% BSA in accordance with the dilution ratio provided in the antibody instructions, and incubated at 4°C overnight. PVDF membrane was washed, secondary antibody was added, and incubated at room temperature for 90 min. 1 × TBST cleaning membrane was used and images were taken using the Tanon imaging system (Tanon, Shanghai, China).

### 2.7. Quantitative real time PCR (qRT-PCR)

qRT-PCR was performed through using SYBR Green PCR Kit (Takara, Dalian, China), the experiment was performed in accordance with the product specification. lncRNA, miRNA and mRNA expression levels were detected by ABI 7900HT fluorescence quantitative PCR, and the relative quantitative analysis of data was performed by 2^−ΔΔCT^. The primer sequences for qRT-PCR are summarized in Table 1.

**Table 1:** Primer sequence of miRNA.

LncRNA	Forward primer	Reverse primer
LINC00853	GAAGCTCAACTTCCGAGGCT	CCGCGGTGTCTGTTCATACT
RP11-885N19.6	CGACTCTAGGCAGGGGAGAT	GGCCTTTGAGGTTTTCCCGA
HLA-DQB1-AS1	TTCTGGGCAGGCATAAGCAG	CTTGGGGGAGGTGATGACAAT
PVT1	AAGAGCCAGTCTTGGTGCTC	CTATGGCATGGGCAGGGTAG
RP11-445P17.8	GGTGCAAACATTGGCAGTTTC	CCCTCAGACCAGAGGTTTCC
AC108004.3	TGCCTGTCTGCTTTTGTCCT	CCCCAGAGAGACCGATGGTA

### 2.8. Statistical analysis

We used the R language for statistical analysis, the psych package for principal component analysis, and the ggplot2 package for three-dimensional mapping. The limma, pheatmap, and ggplot2 packages were used for t-test, heatmap, and volcano maps, respectively. The normalized fluorescence signal values of the selected differential lncRNA and mRNA were imported into the psych package for the Pearson correlation coefficient, and the threshold was set at *r* = 0.9 and *P* < 0.01. The GO and KEGG pathway bubble maps of key lncRNAs were displayed using the clusterProfiler package. All statistical analyses were performed using 0SPSS 20.0 (SPSS, Inc., Chicago, IL, USA) and GraphPad Prism version 8.0.0 (GraphPad Software, San Diego, California USA). Data consistent with normal distribution were expressed as mean ± SD, and comparison between groups was performed by t-test. The data of non-normal distribution were expressed by median, and enumeration data were compared using *χ*^2^ test. The experimental data of qRT–PCR were analyzed using 2^−△△Ct^. *P* < 0.05 indicated statistically significant difference.

## 3. Results

### 3.1. Comparison of basic clinical data

The six patients each in the hypertension and control groups had an average age of 41.50 ± 2.43 and 42.17 ± 2.23 years, respectively. The average age, sex, smoking history, drinking history, body mass index, and levels of fasting glucose, homocysteine (FBG), total cholesterol (TC), triglyceride (TG), high density lipoprotein cholesterol (HDL-C) and low density lipoprotein (LDL-C) of the two groups of patients; no statistically significant difference was observed (*P* > 0.05). Compared with those of the control group, the systolic blood pressure (SBP) and diastolic blood pressure (DBP) values of the hypertensive group were significantly different (*P* < 0.05; Table 2).

**Table 2:** General clinical information.

	Normal (*n* = 6)	Hypertension (*n* = 6)	*P*-value
Age (years)	41.50 ± 2.43	42.17 ± 2.23	0.631^†^
Gender (Male:Female)	5:1	3:3	0.546^‡^
Smoking (Y:N)	1:5	2:4	>0.9999^‡^
Drinking (Y:N)	4:2	3:3	>0.9999^‡^
BMI (kg/m^2^)	25.03 ± 2.18	24.33 ± 1.62	0.541^†^
FBG (mmol/L)	5.38 ± 0.27	5.39 ± 0.37	0.979^†^
TC (mmol/L)	1.59 ± 0.30	1.40 ± 0.37	0.359^†^
TG (mmol/L)	4.76 ± 0.28	4.72 ± 0.28	0.797^†^
HDL-C (mmol/L)	2.73 ± 0.46	2.69 ± 0.42	0.854^†^
LDL-C (mmol/L)	1.50 ± 0.12	1.52 ± 0.09	0.746^†^
SBP (mm Hg)	117.67 ± 7.28	158.50 ± 13.82*	*P *< 0.05^†^
DBP (mm Hg)	74.00 ± 7.59	93.67 ± 16.88*	*P *< 0.05^†^

BMI, body mass index; FBG, fasting blood glucose; TC, total cholesterol; TG, triglycerides; HDL-C, high-density-lipoprotein-cholesterol; LDL-C, low-density-lipoprotein-choles terol; SBP, systolic blood pressure; DBP, diastolic blood pressure.

^†^ Statistical testing by independent-samples t test.

^‡^ Statistical testing by *χ*^2^ test.

* *P* < 0.05 vs. normal group.

### 3.2. Differential expression analysis

**Figure 1:**
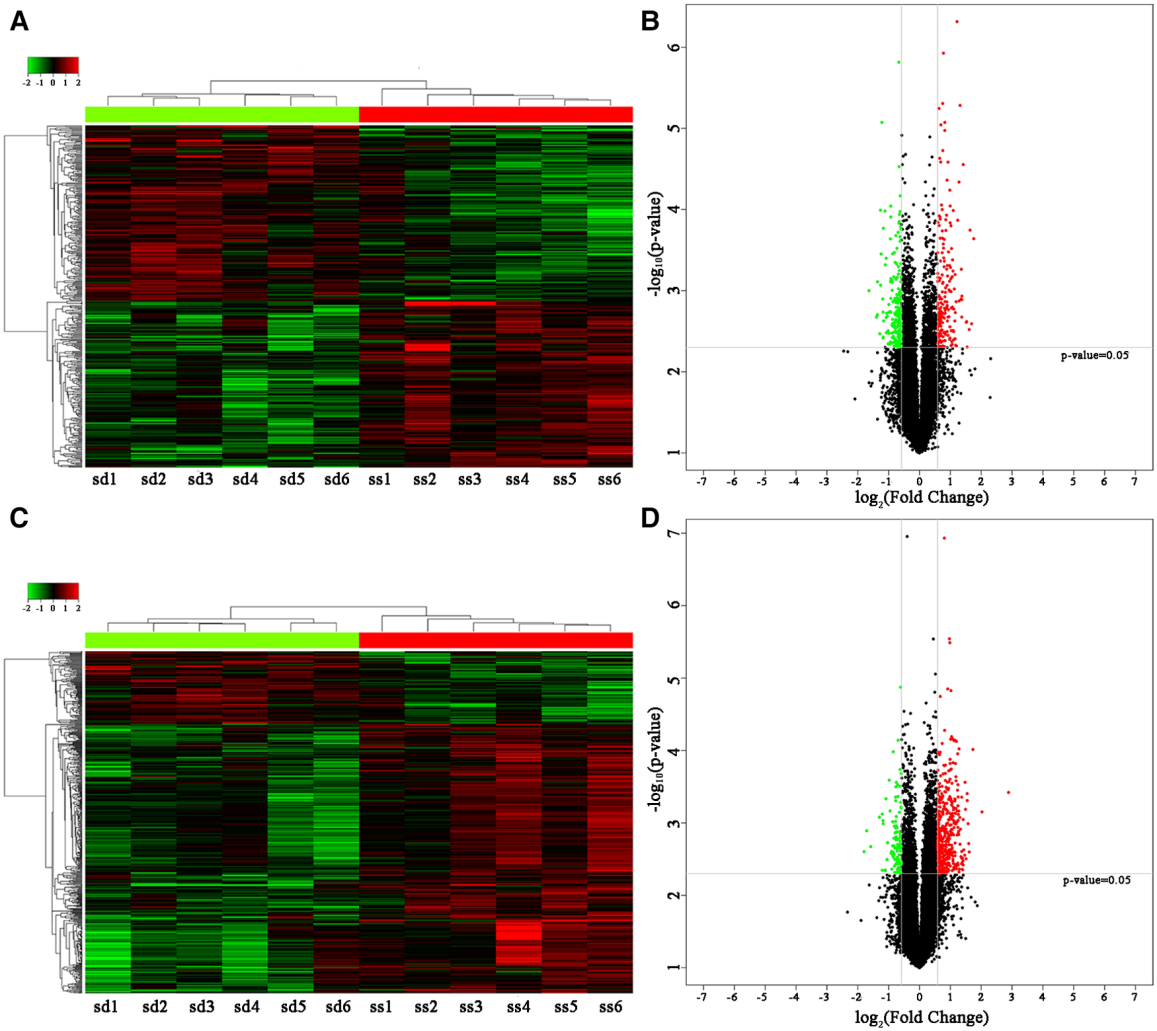
Identification of differentially expressed lncRNAs and mRNAs in the hypertensive and control groups. (**A**) The heatmap of differentially expressed lncRNAs. (**B**) The volcano map showed that a total of 193 upregulated lncRNAs and 203 downregulated lncRNAs were screened out. (**C**) The heatmap of differentially expressed mRNAs. (**D**) The volcano map showed that a total of 411 upregulated mRNAs and 111 downregulated mRNAs were screened out.Red and green represents upregulated and downregulated lncRNAs, respectively. lncRNAs, long non-coding RNAs; mRNAs, messenger RNAs.

A total of 26,112 lncRNAs and 27,046 mRNAs were detected; 396 differentially expressed lncRNAs, i.e., 193 upregulated lncRNAs and 203 downregulated lncRNAs, were detected in the hypertensive and control groups (Supplementary Table S1). Heat and volcano maps were constructed to visualize the differentially expressed lncRNAs in the peripheral blood lymphocytes of the hypertensive and control groups (Figures 1A,B). Concurrently, 511 differentially expressed mRNAs were screened (Supplementary Table S2), among which 402 were upregulated and 109 were downregulated (Figures 1C, D). Table 3 shows the information of the top 10 DE-lncRNAs and DE-mRNAs.

**Table 3:** The top 10 DE-lncRNAs and DE-mRNAs.

	lncRNA	*P*-value	FC	mRNA	*P*-value	FC
Upregulated	RP11-1033H12.1	0.002286	3.39	UTS2	0.007037	4.08
	AC093627.8	0.025532	3.23	CAV2	0.000969	3.32
	LINC00853	0.001793	3.12	COLCA2	0.025169	3.06
	RP11-885N19.6	0.030140	3.06	LGALS2	0.019311	3.02
	HLA-DQB1-AS1	0.049586	2.92	IGJ	0.031267	2.85
	linc-IQCG-1	0.024155	2.89	CDC42EP1	0.002746	2.79
	XR_245347.1	0.024536	2.86	DNAH6	0.038139	2.67
	XR_427724.1	0.000280	2.68	BEND2	0.033072	2.67
	XR_429426.1	0.011667	2.60	OR2L5	0.030834	2.59
	uc021thc.2	0.012912	2.59	KLHL14	0.004403	2.58
Downregulated	RP11-277P12.9	0.010045	3.11	PARD3B	0.024899	3.47
	RNA95583	0.021197	2.63	CCDC83	0.008363	2.46
	linc-MCPH1-2	0.019833	2.57	GTSCR1	0.045155	2.31
	RNA147577	0.007793	2.56	TAS2R41	0.007589	2.30
	uc001vsc.1	0.034042	2.44	PLXDC1	0.009301	2.27
	RP11-445P17.8	0.001022	2.41	RPH3A	0.010278	2.26
	AC108004.3	0.008564	2.39	CPB2	0.044674	2.16
	RP11-392E22.12	0.003538	2.36	OR6N1	0.045800	2.16
	XR_171078.1	0.000084	2.33	SRSF12	0.004644	2.13
	RP11-373D23.3	0.012678	2.28	OR5B12	0.032165	2.07

FC, fold change.

### 3.3. Co-expression and cluster analyses

**Figure 2:**
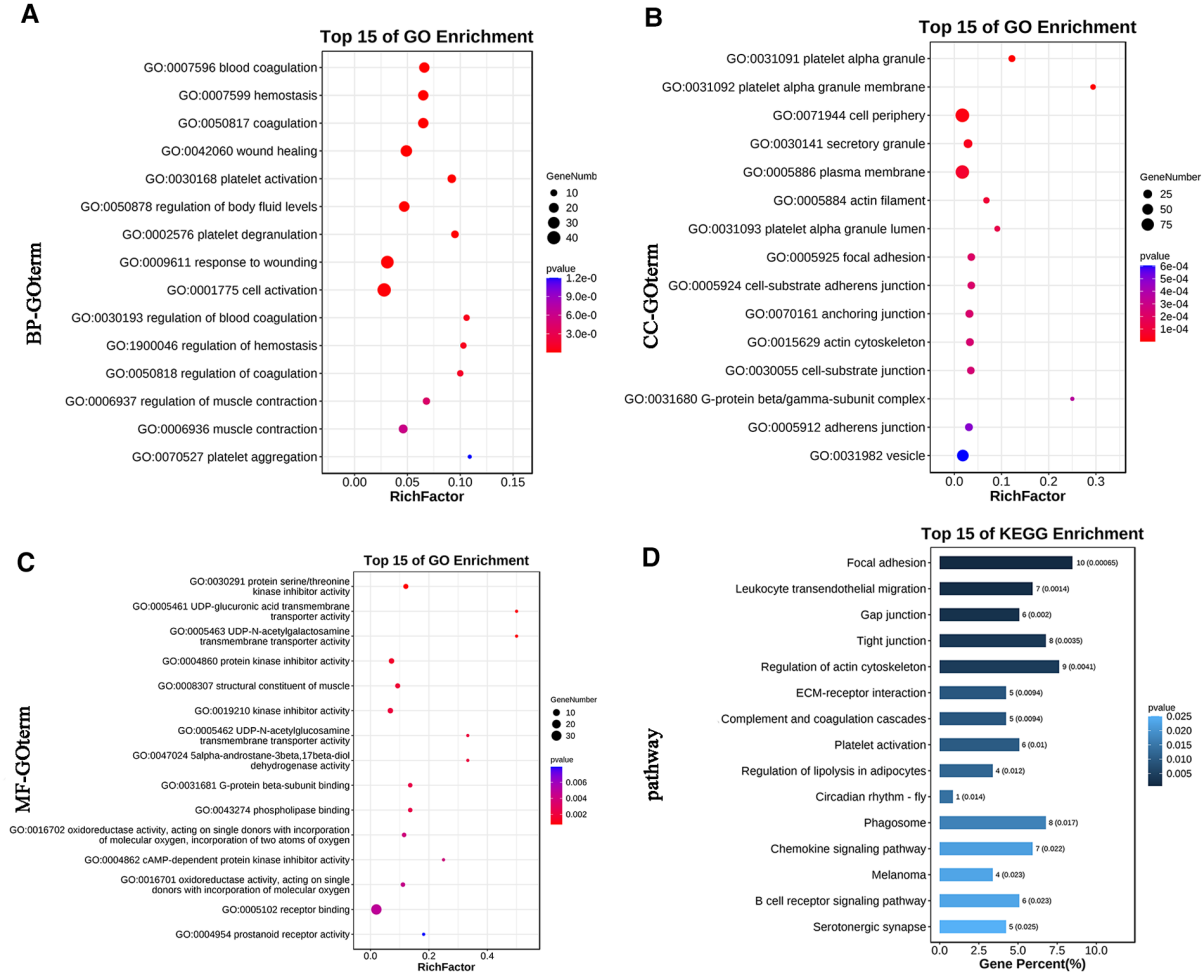
The GO and KEGG pathway enrichment analyses of genes targeted by differentially expressed lncRNAs. (**A**) Bubble Plot of BP. (**B**) Bubble Plot of CC. (**C**) Bubble Plot of MF. (**D**) Bar Plot of KEGG. GO, Gene Ontology; BP, biological processes; CC, cell component; MF, molecular function; KEGG, Kyoto Encyclopedia of Genes and Genomes.

According to the Pearson correlation coefficient matrix calculation method described in the previous section, 1,993 pairs of differential lncRNA-mRNA relationships were obtained, including 255 lncRNAs and 348 mRNAs (Supplementary Table S3). The differential lncRNA-mRNA was analyzed for KEGG pathway and GO function enrichment. The bar graph of GO enrichment analysis results of lncRNA-targeted differential genes depicts the distribution of the number of differential genes in the GO items enriched in biological processes (BP), cellular components (CC), and molecular functions (MF). BP chiefly included regulation of body fluid levels, blood coagulation, and muscle contraction; cell activation; and platelet aggregation (Figure 2A). CC primarily comprised platelet alpha granule, actin cytoskeleton, focal adhesion, cell-substrate adherens junction, actin cytoskeleton, and G-protein beta/gamma-subunit complex (Figure 2B). MF mainly included protein serine/threonine kinase inhibitor activity, UDP-glucuronic acid transmembrane transporter activity, structural constituent of muscle, G-protein beta-subunit binding, cyclic adenosine monophosphate (cAMP)-dependent protein kinase inhibitor activity, and prostanoid receptor activity (Figure 2C). KEGG enrichment analysis of lncRNA targeting differential genes principally included focal adhesion, leukocyte transendothelial migration, gap junction, regulation of actin cytoskeleton, and extracellular matrix (ECM)-receptor interaction (Figure 2D).

### 3.4. Construction of ceRNA regulatory network

**Figure 3:**
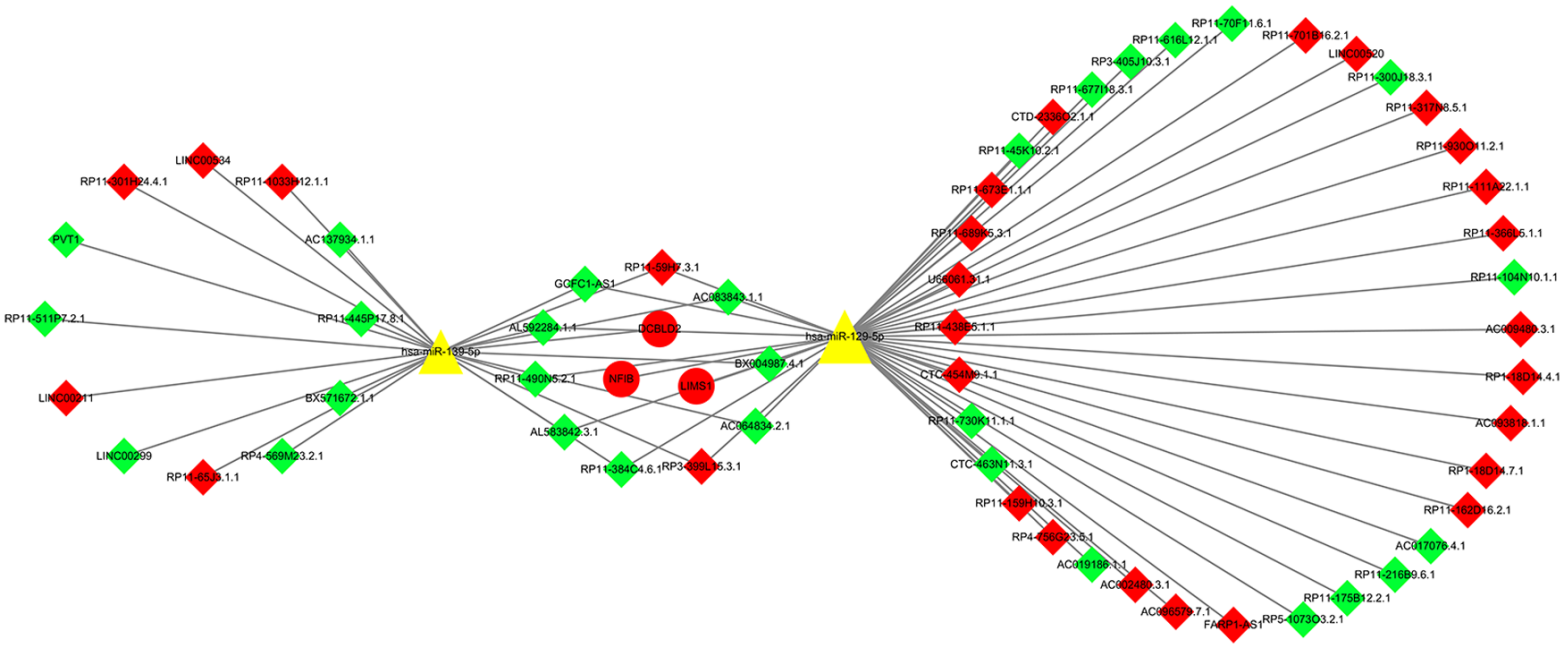
LncRNA-miRNA-mRNA ceRNA network. There were 63 nodes: 2 miRNAs (yellow triangle), 3 mRNAs (round shaped; red representing upregulation), and 58 lncRNAs (diamond shaped; green representing downregulation, red representing upregulation).

The miRcode prediction tool was used to obtain 1962 potential miRNA-lncRNA regulatory relationships, including 86 miRNAs and 128 lncRNAs (Supplementary Table S4). We obtained 107 potential miRNA-mRNA regulatory relationships, including 67 miRNAs and 36 mRNAs (Supplementary Table S5), and finally constructed the ceRNA regulatory network of lncRNA-miRNA-mRNA (Supplementary Table S6). Cytoscape 3.8.2 software was used to visualize the ceRNA regulatory network (Figure 3).

### 3.5. qRT-PCR verification

**Figure 4:**
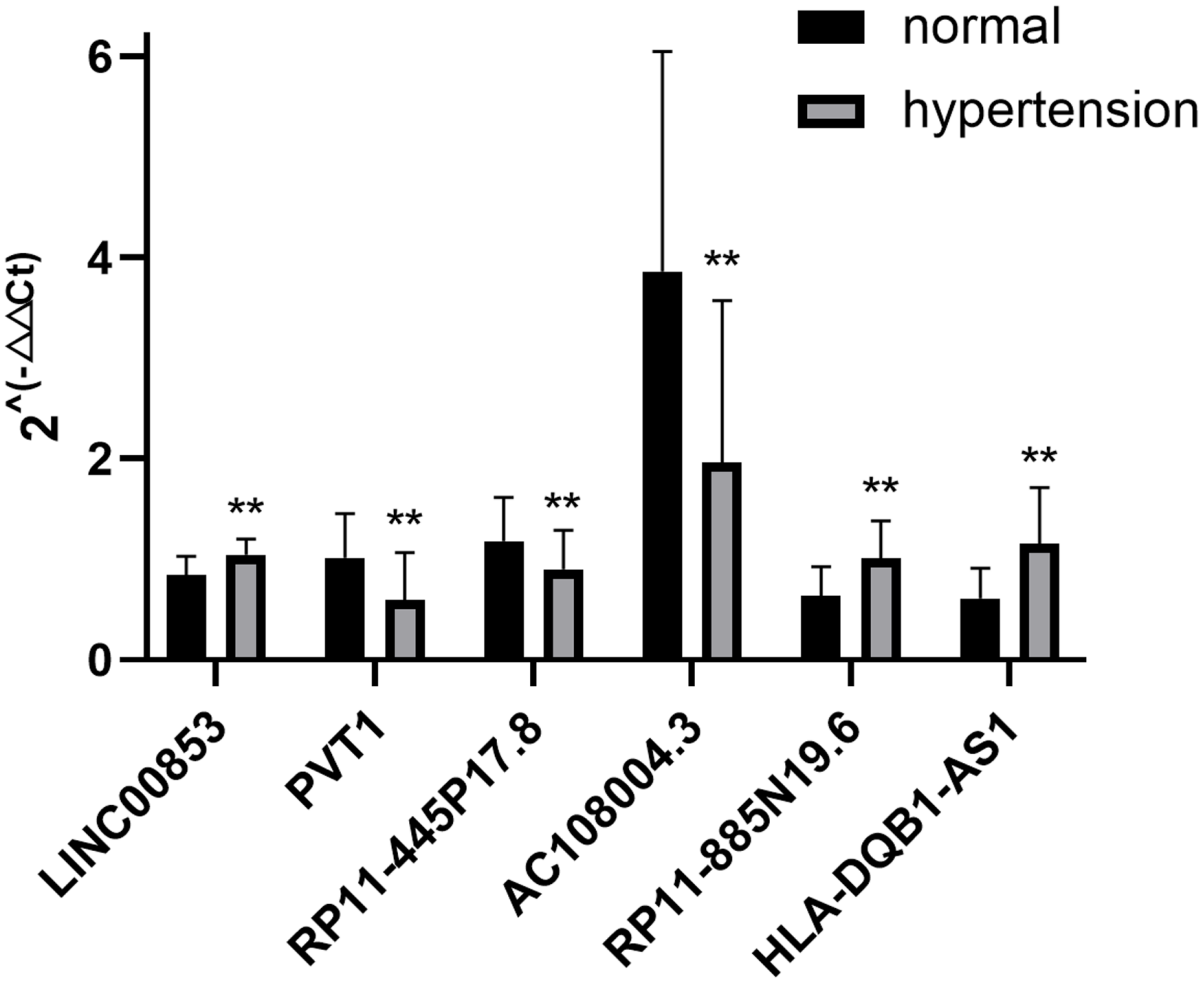
qRT-PCR verification of differentially expressed lncRNAs. n = 30, **P < 0.05 vs. normal.

30 samples were selected for the qRT-PCR test. The characteristics of the subjects are summarized in Supplementary Tables S7. The results revealed that the expression levels of lncRNAs in the hypertension group were statistically significant, compared with that in the control group, with upregulation and downregulation of three lncRNAs each (Figure 4).

### 3.6. PVT1 acts as a ceRNA and regulates the miR-139-5p mRNA target, DCBLD2

**Figure 5:**
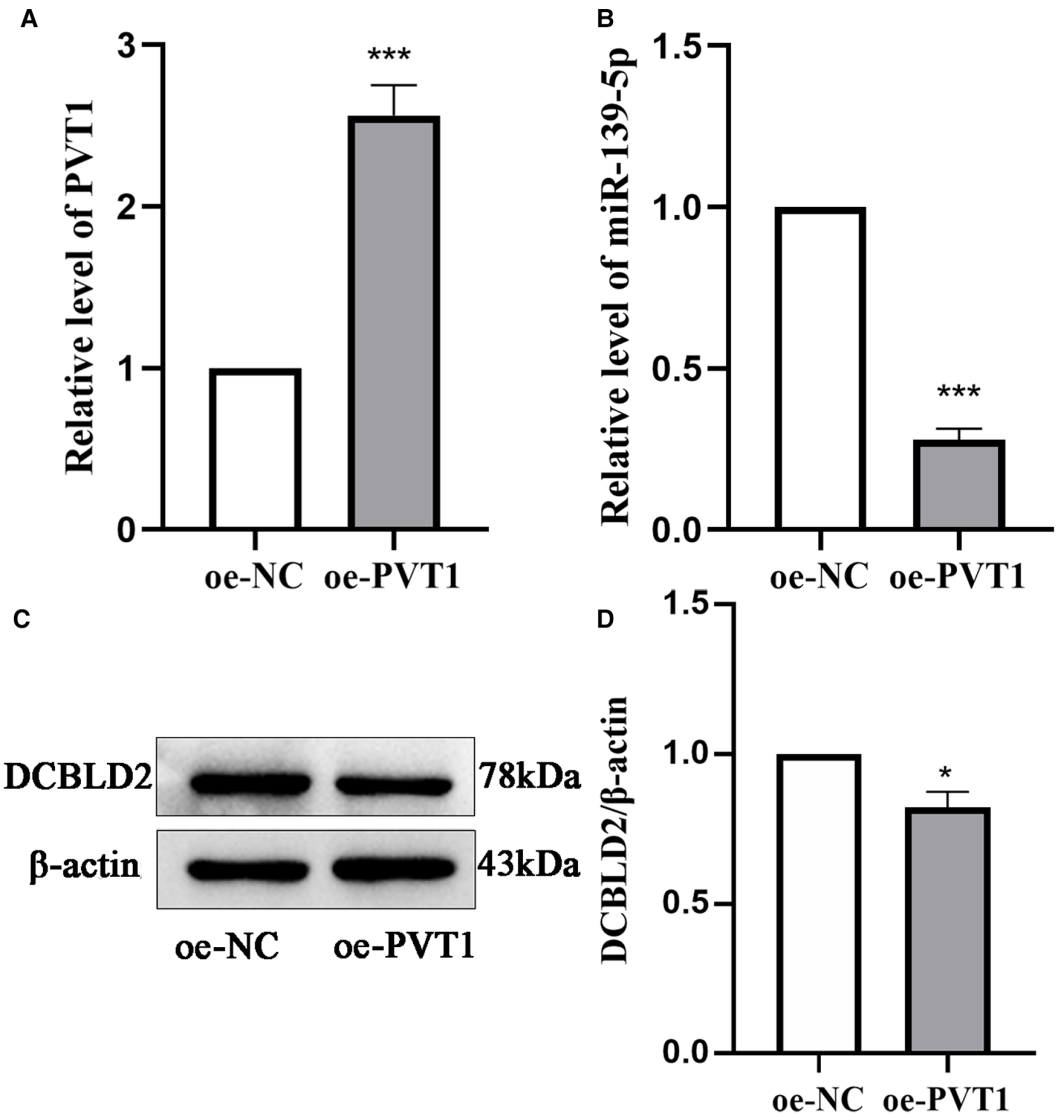
Relationship between PVT1 and the miR-139-5p target, DCBLD2. (**A**) PVT1 expression overexpressed in 293T cells, as analyzed by qRT-PCR. (**B**) The expression level of miR-139-5p was detected by qRT-PCR in 293T cells transfected with PVT1 overexpression plasmid. (**C**) Protein expression levels of DCBLD2 were determined by Western blot. (**D**) Presents the densitometric analysis of the blots normalized to β-actin. Data are reported as mean ± SD. *P < 0.05, ***P < 0.001 vs. oe-NC.

We further elucidated the regulatory relationship between PVT1 and DCBLD2 by overexpressing PVT1 in 293T cells. PCR and Western Blot results showed that overexpression of PVT1 could significantly inhibit the expression of miR-139-5p and DCBLD2. These results indicated that PVT1 acts as a ceRNA of DCBLD2 by regulating miR-139-5p (Figure 5).

## 4. Discussion

Essential hypertension is one of the most important public health concerns, affecting more than 1.2 billion people worldwide and causing an estimated 9.4 million deaths annually (23). Xinjiang has a vast territory and is inhabited by several ethnic groups. Notably, the prevalence rate of hypertension in Kazakh is higher than that in other ethnic groups in the same region (24). Therefore, discovering new diagnostic and therapeutic targets for essential hypertension in Xinjiang Kazakh is essential.

In recent years, the ceRNA hypothesis, as a new mechanism to explain the interaction between RNAs, has become a key topic of research, and various non-coding RNAs, such as ceRNAs, have become a research hotspot for various diseases (25). As a chief factor of post-transcriptional regulation, miRNA activity can be regulated by lncRNA through “sponge” adsorption. As ceRNA, lncRNA competitively binds to miRNA, affecting the ceRNA-induced gene silencing and thereby regulating the protein level of coding genes and participating in the expression regulation of target genes (26). ceRNA can participate in the pathophysiology of hypertension through various molecular functions (27). lncRNA FENDRR plays a positive role in essential hypertension by regulating the miR-423-5p/Nox4 axis, inhibiting cell proliferation and migration and accelerating cell apoptosis, thereby promoting the dysfunction of human umbilical vein endothelial cells (26). However, research on the ceRNA network in Xinjiang Kazakh hypertension has not yet been reported. Therefore, the aim of this study was to identify differentially expressed lncRNAs in the context of hypertension in the Xinjiang Kazakh population and systematically analyze the hypertension-related ceRNA.

In this study, the ceRNA network of lncRNAs in the peripheral blood lymphocytes of Xinjiang Kazakh people with essential hypertension was constructed using the bioinformatics method for the first time. Here, 511 differentially expressed mRNAs and 396 differentially expressed lncRNAs were screened using microarray analysis. Three upregulated lncRNAs and three downregulated lncRNAs were randomly selected for real-time PCR detection in 15 pairs of samples to verify the reliability and reproducibility of our microarray results. The results of qRT-PCR were consistent with the results of microarray scanning, indicating that the microarray scanning results had good reliability.

GO functional clustering of differentially expressed genes exhibited that these differentially expressed mRNAs were predominantly related to the regulation of body fluid levels, actin cytoskeleton, G-protein beta/gamma-subunit complex, cAMP-dependent protein kinase inhibitor activity, and prostanoid receptor activity. Increased vasoconstriction and arterial remodeling are important pathophysiological mechanisms that lead to the development of hypertension. Vascular smooth muscle cells play a crucial role in hypertension development through their highly plastic and dynamic properties, as well as their phenotypic differentiation ability. Increasing evidence suggests that the reorganization of the actin cytoskeleton is involved in the phenotypic differentiation of vascular smooth muscle, switching it from a contractile to a proliferative phenotype (28). AT1R can constrict blood vessels through Gq- and Gi-mediated signaling. Concurrently, Gi signaling enhances Gq-mediated PLC activity and increases intracellular Ca^2+^ levels and synergistic activation of PLCβ3 by Gβγ and Gαq, all of which are key to the regulation of vasoconstriction. SHRs are highly sensitive to ANG II, which may be due to enhanced consistent signaling between the G protein subunits αq (Gαq) and βγ (Gβγ) and PLC (29, 30). The cAMP-protein kinase A (PKA) pathway is involved in various cardiovascular diseases. Activation of the PKA-AMP-activated protein kinase signaling pathway and inhibition of myosin phosphatase targeted subunit 1-myosin light chain phosphorylation can attenuate ANG II-induced contraction of mouse smooth muscle cells *in vitro* and *in vivo* (31). Prostaglandin E2 (PGE2) and E3-class prostanoid (EP3) receptor have previously been shown to modulate blood pressure and hemodynamics in several animal models of hypertension. PGE2 plays an important role in vascular homeostasis, and its receptor E-prostaglandin receptor 4 (EP4) is essential for the physiological remodeling of ductus arteriosus. Vascular smooth muscle cell-specific EP4 gene defects can substantially increase ANG II-induced mesenteric artery vasoconstriction by stimulating intracellular calcium release from the vascular smooth muscle cells, and their dysfunction exacerbates ANG II-induced pathological vascular remodeling (32). The central blockade of EP3 receptor can reduce oxidative stress and inflammation in the paraventricular nucleus of the hypothalamus and regulate neurotransmitters in the paraventricular nucleus of the hypothalamus in SHRs, thereby partially ameliorating hypertension and myocardial hypertrophy (33).

In this study, KEGG enrichment analysis showed that focal adhesion, gap junctions, regulation of actin cytoskeleton, and ECM-receptor interaction were significantly enriched. These signaling pathways interact with each other, which can synergistically affect the occurrence and development of hypertension. Mechanical transduction, as a physiological and pathological signal transduction pathway, is involved in the process of hypertension. Mechanical stimulation of cells can activate mechanosensitive proteins, which are eventually converted into cellular processes, such as focal adhesion, cytoskeleton remodeling, and force-related activation of ion channels. Focal adhesion kinase (FAK) is a mechanotransduction-sensitive protein that plays a critical role in regulating cytoskeleton reorganization, programming cell proliferation, and regulating cell migration (34). FAK is mainly located in the vascular smooth muscle nuclei of healthy arteries, whereas nuclear FAK is inactive. Vascular injury promotes FAK cytoplasmic translocation, in which FAK is activated at the integrin adhesion sites and promotes vascular smooth muscle cell proliferation (35). The ECM reorganization of the vascular wall is closely related to vascular remodeling in hypertension, and ECM interacts with specific integrins and participates in the development and progression of hypertension (36, 37). Integrins are the main family of cell focal adhesion receptors, which have no intrinsic kinase activity. ECM signals are transmitted through serine/threonine kinases, FAK, and proline-rich tyrosine kinase 2 (Pyk2). Alterations in the ECM increase the corresponding integrin activation, resulting in increased activity of FAK or Pyk2, which regulates cell adhesion and signaling of other cell-surface receptors, such as growth factors, cytokines, and G-protein coupled receptors (35, 38, 39). ECM remodeling (elastic fiber disruption, excessive collagen deposition, and rearrangement of stromal cell proteins and proteoglycans) alters the mechanical properties of the vascular wall, exposing the vascular cells to bioactive molecules and playing a key role in hypertension-related vascular remodeling (40, 41). T lymphocytes play a chief role in the regulation of blood pressure, and inhibiting T cell-driven inflammation in target organs can ameliorate or prevent experimental hypertension. Gap junctions are involved in the communication between adjacent cells, and their basic structure comprises connexin. Cx43 is the most important connexin protein in the immune system. It plays a key role in T-cell activation and participates in the proliferation of mature T-cells and secretion of cytokines, thereby playing a role in immune-mediated hypertensive inflammation (42, 43). The accumulation or expression levels of Cx40/Cx43 in SHRs is higher than that in Wistar–Kyoto rats, and the inhibition of Cx43 channels can substantially inhibit the intercellular communication in SHR peripheral blood lymphocytes, thereby inhibiting the expression of inflammatory factors (44).

After software processing, 2 miRNAs, 3 mRNAs, and 58 lncRNAs were obtained to construct the lncRNA-miRNA-mRNA network. Plasmacytoma variant translocation 1 (PVT1) is located in the cancer risk area 8q24.21. lncRNA PVT1 can bind and degrade miR-26b, promote the expression of connective tissue growth factor and angiopoietin-2, and thus regulate the angiogenesis of vascular endothelial cells (45). Moreover, lncRNA PVT1 polymorphisms may be involved in the risk of essential hypertension in the Chinese population by regulating lipid levels (46). *DCBLD2*, a protein-coding gene located on chromosome 3, is a neurofibromin-like transmembrane protein that is upregulated in mRNA levels during arterial remodeling and may be involved in angiogenesis and tumorigenesis progression (47). DCBLD2 plays an important role in vascular remodeling by regulating the endocytosis of platelet-derived growth factor receptor-β in vascular smooth muscle cells through caveolin-1 (48). It reportedly induces endothelial cell proliferation, migration, and signal transduction through the regulation of the VEGFR-2–VE-cadherin/protein tyrosine phosphatase complex. Thus, DCBLD2 may act as a target to regulate angiogenesis (49). Therefore, we hypothesized that lncRNA PVT1-miR-139-5p-DCBLD2 has a potential ceRNA regulatory mechanism in the occurrence of essential hypertension in Xinjiang Kazakh and that all RNAs in this ceRNA network may be biomarkers for the diagnosis of essential hypertension in this population. In this study, we overexpressed lncRNA PVT1 to inhibit the expression of miR-139-5p and DCBLD2, indicating that DCBLD2 is regulated by the lncRNA PVT1/miR-139-5p axis in 293T cells. Our study reveals a new ceRNA regulatory network, lncRNA PVT1-miR-139-5p-DCBLD2, providing new insights into the mechanism of essential hypertension in Xinjiang Kazakh. The potential role of lncRNA PVT1-miR-139-5p-DCBLD2 in the process of essential hypertension in Xinjiang Kazakh needs to be further verified in future studies.

This study has some limitations. The number of included samples was limited; this may lead to some bias in the number of screened lncRNAs and mRNAs. Some analyzed biological processes may also be missing. We used only bioinformatics methods for preliminary exploration in this study. Therefore, future cell and animal experiments are needed to further study the core ceRNA network and its key lncRNAs.

## 5. Conclusions

In this study, by analyzing the differences in lncRNA and mRNA expression in the peripheral blood lymphocytes of Xinjiang Kazakh people with essential hypertension, we successfully constructed the relevant ceRNA regulatory network. Concurrently, our findings revealed that the differentially expressed lncRNA targeted genes were enriched in focal adhesion, gap junctions, regulation of actin cytoskeleton, ECM-receptor interaction, and other pathways involved in the pathogenesis of hypertension. Furthermore, we constructed a ceRNA regulatory network related to differentially expressed lncRNAs, among which lncRNA PVT1-miR-139-5p-DCBLD2 may have a potential ceRNA regulatory mechanism in the occurrence of essential hypertension in Xinjiang Kazakh people.

## Data Availability

The data presented in the study are deposited in the Gene Expression Omnibus (GEO) (https://www.ncbi.nlm.nih.gov/geo/query/acc.cgi?acc=GSE234085), accession number GSE234085.
