# Supplementary material for: Bioinformatics analysis of lncRNA-related ceRNA networks in the peripheral blood lymphocytes of Kazakh patients with essential hypertension in Xinjiang
Source: Front Cardiovasc Med. 2023 Jun 15;10:1155767. doi: 10.3389/fcvm.2023.1155767 (PMC10311024; doi:10.3389/fcvm.2023.1155767)
Supplement: Supplementary file 1 [file Table1.docx]

Supplementary Material

Bioinformatics analysis of lncRNA-related ceRNA networks in the peripheral blood lymphocytes of Kazakh patients with essential hypertension in Xinjiang

**Yan Wang^1,2+^,Jie Gao^1,2,3+^, Liang Zhang ^1,2,3^, Rui Yang^1,2,3,^ Yingying Zhang^1,2,3^, Liya Shan^1,2,3^, Xinzhi Li^1,2,4*^ and Ketao Ma ^1,2,3*^**

*** Correspondence:**

Corresponding Author: Ketao Ma
[maketao@hotmail.com](mailto:maketao@hotmail.com).

Corresponding Author: Xinzhi Li
[lixinzhi@shzu.edu.cn](mailto:lixinzhi@shzu.edu.cn).

# Table S1. All DE-lncRNAs between hypertensive and control groups

| **DE-lncRNAs** | P-value | **FC** | **Regulation** |
| --- | --- | --- | --- |
| RP11-1033H12.1 | 0.002286 | 3.39 | up |
| AC093627.8 | 0.025532 | 3.23 | up |
| LINC00853 | 0.001793 | 3.12 | up |
| RP11-885N19.6 | 0.030140 | 3.06 | up |
| HLA-DQB1-AS1 | 0.049586 | 2.92 | up |
| linc-IQCG-1 | 0.024155 | 2.89 | up |
| XR_245347.1 | 0.024536 | 2.86 | up |
| XR_427724.1 | 0.000280 | 2.68 | up |
| XR_429426.1 | 0.011667 | 2.60 | up |
| uc021thc.2 | 0.012912 | 2.59 | up |
| linc-NDST3-8 | 0.021189 | 2.58 | up |
| FARP1-AS1 | 0.005460 | 2.56 | up |
| uc001yuj.2 | 0.012727 | 2.55 | up |
| RP11-888D10.3 | 0.000052 | 2.49 | up |
| XR_429429.1 | 0.013419 | 2.44 | up |
| XR_430056.1 | 0.000458 | 2.43 | up |
| RP11-59H7.3 | 0.001356 | 2.36 | up |
| linc-LAMA1-5 | 0.000005 | 2.33 | up |
| RP11-1002K11.1 | 0.047899 | 2.29 | up |
| NAV2-AS5 | 0.013709 | 2.28 | up |
| AC002480.4 | 0.047118 | 2.26 | up |
| RP11-568N6.1 | 0.018636 | 2.24 | up |
| AC002480.3 | 0.039250 | 2.23 | up |
| RP13-578N3.3 | 0.006336 | 2.23 | up |
| HIT000651893 | 0.023637 | 2.21 | up |
| RP11-701B16.3 | 0.009537 | 2.19 | up |
| RP11-384J4.1 | 0.016916 | 2.17 | up |
| CTB-111H14.1 | 0.032020 | 2.15 | up |
| uc021vkt.1 | 0.040339 | 2.15 | up |
| RP11-185E8.1 | 0.001031 | 2.14 | up |
| HIT000078556_03 | 0.030988 | 2.14 | up |
| RP11-701B16.3 | 0.005717 | 2.12 | up |
| RP11-930O11.2 | 0.004180 | 2.10 | up |
| uc002sti.1 | 0.015911 | 2.10 | up |
| RP11-556E13.1 | 0.006721 | 2.09 | up |
| XR_429366.1 | 0.046104 | 2.09 | up |
| RNA147248 | 0.042296 | 2.07 | up |
| LINC00520 | 0.003032 | 2.06 | up |
| linc-TREM2-1 | 0.016404 | 2.04 | up |
| CTC-454M9.1 | 0.031176 | 2.04 | up |
| uc031qlc.1 | 0.047491 | 2.04 | up |
| linc-TECTB-2 | 0.010248 | 2.03 | up |
| RP11-301O19.1 | 0.001588 | 2.03 | up |
| linc-UBE2E1-1 | 0.007118 | 2.03 | up |
| ASO1647 | 0.005190 | 2.01 | up |
| AC093415.2 | 0.001458 | 1.99 | up |
| XR_427192.1 | 0.000906 | 1.99 | up |
| RNA143600 | 0.038244 | 1.99 | up |
| LIT2061 | 0.027893 | 1.98 | up |
| AC078883.3 | 0.000578 | 1.98 | up |
| uc003qls.2 | 0.010172 | 1.97 | up |
| RNA147249 | 0.039797 | 1.97 | up |
| uc022caj.1 | 0.004909 | 1.97 | up |
| NR_026800.1 | 0.007656 | 1.97 | up |
| RP11-556E13.1 | 0.009796 | 1.94 | up |
| linc-POTEC-3 | 0.021046 | 1.94 | up |
| LINC00520 | 0.003341 | 1.92 | up |
| RP11-326A19.3 | 0.017190 | 1.92 | up |
| RNA143521 | 0.035528 | 1.90 | up |
| CTC-454M9.1 | 0.000261 | 1.90 | up |
| AC096579.7 | 0.021155 | 1.90 | up |
| linc-TMEM206-5 | 0.006612 | 1.88 | up |
| AC093415.2 | 0.023612 | 1.87 | up |
| STARD13-AS2 | 0.032001 | 1.87 | up |
| AC093818.1 | 0.004910 | 1.87 | up |
| RP1-118J21.5 | 0.015228 | 1.86 | up |
| RP11-930O11.1 | 0.008610 | 1.86 | up |
| WDR11-AS1 | 0.000434 | 1.86 | up |
| LIT2094 | 0.015944 | 1.85 | up |
| RP11-561I11.4 | 0.013765 | 1.85 | up |
| uc004ehp.2 | 0.005423 | 1.84 | up |
| RP11-673E1.1 | 0.001176 | 1.84 | up |
| ARHGEF26-AS1 | 0.001807 | 1.81 | up |
| NR_037616.1 | 0.001340 | 1.81 | up |
| RP11-259P20.1 | 0.009251 | 1.80 | up |
| XR_426860.1 | 0.016738 | 1.79 | up |
| linc-DUSP4-6 | 0.034654 | 1.79 | up |
| RP1-86C11.7 | 0.010495 | 1.79 | up |
| linc-C14orf101-4 | 0.041326 | 1.78 | up |
| LINC00211 | 0.019015 | 1.78 | up |
| XR_242672.2 | 0.017463 | 1.78 | up |
| RP11-159H10.3 | 0.032500 | 1.78 | up |
| RP11-874J12.4 | 0.013730 | 1.77 | up |
| XR_430341.1 | 0.011895 | 1.77 | up |
| AC093415.2 | 0.000106 | 1.77 | up |
| CTC-454M9.1 | 0.000084 | 1.77 | up |
| linc-CDH9-4 | 0.041189 | 1.76 | up |
| RP11-77H9.8 | 0.016455 | 1.76 | up |
| RP5-977B1.11 | 0.048858 | 1.76 | up |
| AC098617.1 | 0.019132 | 1.76 | up |
| linc-C14orf101-5 | 0.004339 | 1.75 | up |
| linc-GCNT2-3 | 0.002330 | 1.74 | up |
| AC007319.1 | 0.021918 | 1.74 | up |
| linc-LYPLAL1-1 | 0.005093 | 1.74 | up |
| AC093415.2 | 0.003954 | 1.73 | up |
| STARD13-AS | 0.033273 | 1.72 | up |
| linc-FGD3-2 | 0.002985 | 1.72 | up |
| CTC-454M9.1 | 0.000012 | 1.71 | up |
| linc-COX19-1 | 0.002664 | 1.71 | up |
| linc-MC4R-1 | 0.019132 | 1.70 | up |
| TPTEP1 | 0.036886 | 1.70 | up |
| KB-1460A1.3 | 0.007903 | 1.70 | up |
| uc010vhc.3 | 0.021098 | 1.69 | up |
| RP11-366L5.1 | 0.000187 | 1.69 | up |
| U66061.31 | 0.048000 | 1.69 | up |
| RP11-115D19.1 | 0.014715 | 1.69 | up |
| XR_430238.1 | 0.000049 | 1.68 | up |
| RP11-930O11.1 | 0.008120 | 1.68 | up |
| XXbac-BPG254F23.6 | 0.024718 | 1.68 | up |
| NR_027145.2 | 0.002252 | 1.67 | up |
| LINC00163 | 0.010371 | 1.67 | up |
| RNA95734 | 0.001838 | 1.67 | up |
| XR_242690.1 | 0.018637 | 1.67 | up |
| AC009480.3 | 0.003413 | 1.67 | up |
| RP11-301H24.4 | 0.001003 | 1.67 | up |
| RP11-768F21.1 | 0.006392 | 1.66 | up |
| linc-FAM19A5-1 | 0.030705 | 1.66 | up |
| LINC00534 | 0.041986 | 1.66 | up |
| linc-DOCK3-1 | 0.021257 | 1.65 | up |
| XR_108954.2 | 0.012678 | 1.65 | up |
| AC093159.1 | 0.030270 | 1.64 | up |
| linc-TMEM206-5 | 0.000880 | 1.64 | up |
| RP11-597D13.9 | 0.020928 | 1.63 | up |
| linc-MC4R-1 | 0.019409 | 1.62 | up |
| RP11-16B13.1 | 0.043325 | 1.62 | up |
| RP11-190J1.10 | 0.014089 | 1.62 | up |
| RP11-872D17.4 | 0.016085 | 1.62 | up |
| RP11-490O6.2 | 0.027444 | 1.62 | up |
| RP11-597D13.9 | 0.036007 | 1.62 | up |
| linc-LAMA1-5 | 0.000091 | 1.62 | up |
| TCONS_00013375 | 0.022923 | 1.62 | up |
| RP11-498E2.9 | 0.017919 | 1.61 | up |
| RP4-756G23.5 | 0.024765 | 1.61 | up |
| AC012501.2 | 0.020427 | 1.61 | up |
| RP13-942N8.1 | 0.000260 | 1.61 | up |
| RP11-438E5.1 | 0.004313 | 1.60 | up |
| uc003fap.1 | 0.034288 | 1.60 | up |
| RP11-492A10.1 | 0.031736 | 1.60 | up |
| AFAP1-AS1 | 0.024461 | 1.60 | up |
| AC147651.3 | 0.007008 | 1.60 | up |
| XR_246348.2 | 0.048372 | 1.60 | up |
| RP11-65J3.1 | 0.032812 | 1.59 | up |
| RP3-399L15.3 | 0.001313 | 1.59 | up |
| XXbac-BPG170G13.32 | 0.043057 | 1.59 | up |
| RP11-672L10.6 | 0.023460 | 1.59 | up |
| TAPT1-AS1 | 0.033500 | 1.59 | up |
| linc-DHFRL1-4 | 0.002259 | 1.59 | up |
| RP11-689K5.3 | 0.034735 | 1.58 | up |
| RP11-248J18.2 | 0.040787 | 1.58 | up |
| linc-PRPS1L1-1 | 0.012006 | 1.58 | up |
| RP11-142C4.6 | 0.001233 | 1.58 | up |
| ASO3535 | 0.012761 | 1.58 | up |
| RP11-162D16.2 | 0.026320 | 1.58 | up |
| linc-CD1D-1 | 0.034576 | 1.58 | up |
| linc-YWHAZ-1 | 0.021261 | 1.58 | up |
| XR_426970.1 | 0.017261 | 1.58 | up |
| AC093415.2 | 0.000234 | 1.57 | up |
| RP11-52A20.2 | 0.024930 | 1.57 | up |
| RP11-701B16.2 | 0.007516 | 1.57 | up |
| AC139100.3 | 0.025030 | 1.57 | up |
| XR_428603.1 | 0.023479 | 1.56 | up |
| uc.435- | 0.025367 | 1.56 | up |
| RP1-18D14.7 | 0.001197 | 1.56 | up |
| RP11-317N8.5 | 0.001577 | 1.56 | up |
| NR_026710.1 | 0.023684 | 1.56 | up |
| linc-PRPS1L1-1 | 0.021809 | 1.56 | up |
| RP11-116G8.5 | 0.036518 | 1.55 | up |
| RP5-887A10.1 | 0.048261 | 1.55 | up |
| XR_427746.1 | 0.000057 | 1.55 | up |
| linc-NBAS-9 | 0.043466 | 1.55 | up |
| XR_428102.1 | 0.043429 | 1.55 | up |
| RNASEH2B-AS1 | 0.020102 | 1.55 | up |
| linc-LYZL2-1 | 0.011457 | 1.55 | up |
| RP1-90J20.7 | 0.018674 | 1.54 | up |
| linc-GRHL2-4 | 0.021224 | 1.54 | up |
| eHIT000015952 | 0.036831 | 1.53 | up |
| linc-KLF8-1 | 0.034219 | 1.53 | up |
| RP11-672A2.4 | 0.037616 | 1.53 | up |
| RP11-111A22.1 | 0.042049 | 1.53 | up |
| CTD-2336O2.1 | 0.027214 | 1.53 | up |
| XR_428739.1 | 0.027751 | 1.52 | up |
| RP11-368D24__A.1 | 0.001445 | 1.52 | up |
| RP11-701B16.2 | 0.014246 | 1.52 | up |
| XR_108564.1 | 0.027913 | 1.51 | up |
| RP11-15I20.1 | 0.014873 | 1.51 | up |
| XR_241556.1 | 0.016417 | 1.51 | up |
| RP11-91G21.1 | 0.006891 | 1.51 | up |
| NR_110177.1 | 0.006145 | 1.51 | up |
| linc-LARGE-3 | 0.044525 | 1.51 | up |
| linc-MKI67-4 | 0.030133 | 1.51 | up |
| XR_428101.1 | 0.003409 | 1.51 | up |
| NR_037928.1 | 0.007678 | 1.50 | up |
| RP1-90J20.7 | 0.035010 | 1.50 | up |
| RP11-277P12.9 | 0.010045 | 3.11 | down |
| RNA95583 | 0.021197 | 2.63 | down |
| linc-MCPH1-2 | 0.019833 | 2.57 | down |
| RNA147577 | 0.007793 | 2.56 | down |
| uc001vsc.1 | 0.034042 | 2.44 | down |
| RP11-445P17.8 | 0.001022 | 2.41 | down |
| AC108004.3 | 0.008564 | 2.39 | down |
| RP11-392E22.12 | 0.003538 | 2.36 | down |
| XR_171078.1 | 0.000084 | 2.33 | down |
| RP11-373D23.3 | 0.012678 | 2.28 | down |
| ZNF252P-AS1 | 0.038410 | 2.26 | down |
| RP3-405J10.3 | 0.018401 | 2.26 | down |
| RP11-466P24.7 | 0.037478 | 2.26 | down |
| RP11-350N15.6 | 0.001706 | 2.25 | down |
| uc002tzb.1 | 0.023672 | 2.24 | down |
| INTS6-AS1 | 0.001042 | 2.17 | down |
| RP3-466P17.1 | 0.004806 | 2.15 | down |
| XR_429339.1 | 0.021499 | 2.11 | down |
| RNA147084 | 0.003992 | 2.11 | down |
| NR_026658.1 | 0.033140 | 2.10 | down |
| linc-TNRC6C-2 | 0.019017 | 2.10 | down |
| RNF144A-AS1 | 0.020692 | 2.06 | down |
| PAXBP1-AS1 | 0.031254 | 2.02 | down |
| RP5-944M2.2 | 0.045139 | 2.02 | down |
| uc002ott.1 | 0.018589 | 1.99 | down |
| RP11-1094M14.11 | 0.033474 | 1.99 | down |
| RP11-76E17.3 | 0.014845 | 1.99 | down |
| linc-C20orf29-1 | 0.045298 | 1.96 | down |
| XR_242712.2 | 0.018151 | 1.94 | down |
| RNA146910 | 0.002304 | 1.92 | down |
| RP11-458D21.1 | 0.007705 | 1.92 | down |
| RP11-104N10.1 | 0.042746 | 1.92 | down |
| RP11-640M9.1 | 0.000910 | 1.91 | down |
| linc-LYZL2-4 | 0.012468 | 1.90 | down |
| int-HOXB3-66 | 0.001235 | 1.90 | down |
| NR_036530.1 | 0.007108 | 1.90 | down |
| RP11-399O19.8 | 0.018427 | 1.89 | down |
| RNA95672 | 0.033515 | 1.87 | down |
| hox-HOXB3-74 | 0.007384 | 1.86 | down |
| uc003tgm.1 | 0.026688 | 1.85 | down |
| RP11-514F3.5 | 0.009908 | 1.85 | down |
| RP1-197B17.3 | 0.007908 | 1.84 | down |
| BX571672.1 | 0.037967 | 1.84 | down |
| linc-SDHC-1 | 0.044305 | 1.84 | down |
| RP11-47J17.2 | 0.022626 | 1.83 | down |
| TTTY21 | 0.003517 | 1.83 | down |
| NR_110099.1 | 0.025186 | 1.82 | down |
| RP4-569M23.2 | 0.027549 | 1.82 | down |
| AL109761.5 | 0.013704 | 1.82 | down |
| XR_246176.1 | 0.037198 | 1.81 | down |
| XR_429354.1 | 0.036610 | 1.80 | down |
| RNA147574 | 0.032985 | 1.80 | down |
| CTD-2666L21.1 | 0.038532 | 1.78 | down |
| RP11-108M9.3 | 0.006748 | 1.78 | down |
| NR_026880.1 | 0.013409 | 1.77 | down |
| RNA95727 | 0.048504 | 1.76 | down |
| int-HOXB3-91 | 0.003392 | 1.76 | down |
| LIT1273 | 0.042404 | 1.75 | down |
| linc-SLC25A13-1 | 0.026989 | 1.75 | down |
| RP11-219O3.2 | 0.047918 | 1.75 | down |
| ASO2050 | 0.019180 | 1.75 | down |
| PTCHD3P1 | 0.013041 | 1.74 | down |
| RP11-45K10.2 | 0.012479 | 1.74 | down |
| RP11-175B12.2 | 0.024641 | 1.74 | down |
| RP11-76E17.3 | 0.028778 | 1.73 | down |
| AC064834.2 | 0.039646 | 1.73 | down |
| RP11-13N13.6 | 0.004485 | 1.73 | down |
| AC005682.5 | 0.043175 | 1.72 | down |
| uc002vvv.3 | 0.031448 | 1.72 | down |
| linc-TNFRSF9-1 | 0.012914 | 1.72 | down |
| LINC00623 | 0.023683 | 1.72 | down |
| linc-CHD9-11 | 0.012838 | 1.72 | down |
| RP11-384C4.7 | 0.019952 | 1.71 | down |
| BCDIN3D-AS1 | 0.022786 | 1.70 | down |
| linc-SLC25A27-4 | 0.045887 | 1.70 | down |
| HIT000079323 | 0.012561 | 1.70 | down |
| linc-ST6GAL2-9 | 0.007119 | 1.70 | down |
| AC005895.3 | 0.007249 | 1.70 | down |
| linc-FAM153C-1 | 0.047282 | 1.70 | down |
| RP11-300J18.3 | 0.002096 | 1.70 | down |
| linc-IFIT2-1 | 0.023698 | 1.70 | down |
| RP11-110I1.14 | 0.014001 | 1.69 | down |
| linc-DIRAS2-4 | 0.022557 | 1.69 | down |
| BX004987.4 | 0.016009 | 1.69 | down |
| RNA147028 | 0.032743 | 1.69 | down |
| TMEM254-AS1 | 0.018187 | 1.68 | down |
| nc-HOXB4-167 | 0.028635 | 1.68 | down |
| NR_047651.1 | 0.004637 | 1.68 | down |
| MIR302B | 0.019538 | 1.67 | down |
| linc-SLC25A13-1 | 0.042812 | 1.67 | down |
| RP11-737O24.2 | 0.004701 | 1.67 | down |
| RP11-677I18.3 | 0.044180 | 1.66 | down |
| RP1-122O8.7 | 0.011127 | 1.66 | down |
| ASO1737 | 0.043961 | 1.66 | down |
| RNA146964 | 0.012246 | 1.66 | down |
| RP11-216B9.6 | 0.003014 | 1.65 | down |
| RP3-323P24.3 | 0.020383 | 1.65 | down |
| uc003jxa.1 | 0.019998 | 1.65 | down |
| ASO3932 | 0.006859 | 1.65 | down |
| RNA147009 | 0.049078 | 1.64 | down |
| H19 | 0.048255 | 1.64 | down |
| int-HOXB3-67 | 0.013345 | 1.64 | down |
| RP11-14C22.6 | 0.049936 | 1.64 | down |
| linc-PROKR2-4 | 0.041474 | 1.64 | down |
| RP11-255A11.21 | 0.014116 | 1.64 | down |
| RP11-214O1.3 | 0.048736 | 1.63 | down |
| RP11-396C23.4 | 0.018285 | 1.63 | down |
| HOTTIP | 0.027576 | 1.63 | down |
| AC019186.1 | 0.048890 | 1.63 | down |
| AKAP11-IT1 | 0.014264 | 1.63 | down |
| RP11-7F18.2 | 0.016090 | 1.63 | down |
| XR_426862.1 | 0.013436 | 1.62 | down |
| RP5-827C21.2 | 0.037472 | 1.62 | down |
| uc001lku.1 | 0.032559 | 1.62 | down |
| EGFLAM-AS3 | 0.017351 | 1.62 | down |
| CTC-463N11.3 | 0.031623 | 1.62 | down |
| linc-FCGR1A-3 | 0.001917 | 1.62 | down |
| NR_022010.1 | 0.026461 | 1.62 | down |
| RP11-214O1.2 | 0.025142 | 1.62 | down |
| uc001ttj.1 | 0.013869 | 1.61 | down |
| LL09NC01-139C3.1 | 0.034170 | 1.61 | down |
| RP11-476C8.3 | 0.005065 | 1.61 | down |
| PRMT5-AS1 | 0.009504 | 1.61 | down |
| linc-WDR26-3 | 0.006263 | 1.60 | down |
| CTC-523E23.1 | 0.024952 | 1.60 | down |
| RP11-715F3.2 | 0.041841 | 1.60 | down |
| RP11-250B2.6 | 0.011257 | 1.60 | down |
| RP11-173C20.2 | 0.036147 | 1.60 | down |
| LINC00663 | 0.023161 | 1.60 | down |
| RNA95016 | 0.020867 | 1.60 | down |
| RP5-1073O3.2 | 0.018477 | 1.60 | down |
| XR_158905.1 | 0.011140 | 1.59 | down |
| AC009531.2 | 0.017990 | 1.59 | down |
| AC087294.2 | 0.000015 | 1.59 | down |
| AC114273.1 | 0.021171 | 1.59 | down |
| AC105461.1 | 0.046190 | 1.59 | down |
| AC137934.1 | 0.040711 | 1.58 | down |
| uc002tdp.4 | 0.001476 | 1.58 | down |
| RP11-445P17.3 | 0.019675 | 1.58 | down |
| AC017002.2 | 0.031097 | 1.58 | down |
| NR_003703.1 | 0.000297 | 1.58 | down |
| XR_427244.1 | 0.036487 | 1.58 | down |
| RNA147330 | 0.049432 | 1.58 | down |
| XR_430436.1 | 0.020267 | 1.57 | down |
| LINC00623 | 0.001404 | 1.57 | down |
| linc-USP14-3 | 0.012480 | 1.57 | down |
| AL583842.3 | 0.020808 | 1.57 | down |
| linc-SPATA7-3 | 0.010475 | 1.57 | down |
| linc-TAGAP-1 | 0.005681 | 1.57 | down |
| NR_027034.1 | 0.001150 | 1.57 | down |
| RNA95005 | 0.013426 | 1.56 | down |
| RP11-235G24.1 | 0.019789 | 1.56 | down |
| uc003kmx.1 | 0.043983 | 1.56 | down |
| RP11-730K11.1 | 0.026901 | 1.56 | down |
| XR_244697.1 | 0.030191 | 1.56 | down |
| AC083843.1 | 0.031476 | 1.56 | down |
| XR_245040.2 | 0.031679 | 1.56 | down |
| linc-CCR8-1 | 0.011385 | 1.56 | down |
| RP11-661A12.5 | 0.013644 | 1.55 | down |
| RP11-490N5.2 | 0.031782 | 1.55 | down |
| linc-TCERG1L-3 | 0.032507 | 1.55 | down |
| XR_245446.2 | 0.002167 | 1.55 | down |
| LINC00299 | 0.047707 | 1.55 | down |
| ASO1895 | 0.002460 | 1.55 | down |
| NR_046135.1 | 0.001060 | 1.54 | down |
| XR_428131.1 | 0.010249 | 1.54 | down |
| AC069257.8 | 0.000678 | 1.54 | down |
| CTD-2560E9.3 | 0.012496 | 1.54 | down |
| NR_024586.1 | 0.011095 | 1.54 | down |
| NBPF1 | 0.023685 | 1.53 | down |
| RP11-296I10.3 | 0.017691 | 1.53 | down |
| RP11-511P7.2 | 0.004660 | 1.53 | down |
| CTC-523E23.4 | 0.016453 | 1.53 | down |
| AP001619.2 | 0.010003 | 1.53 | down |
| linc-TCERG1L-1 | 0.021729 | 1.53 | down |
| linc-NME4-1 | 0.042012 | 1.53 | down |
| RP11-384C4.6 | 0.030414 | 1.53 | down |
| RP11-53O19.1 | 0.010384 | 1.53 | down |
| linc-ZNF30-3 | 0.048365 | 1.53 | down |
| linc-DHX37-4 | 0.041969 | 1.53 | down |
| RP11-109E24.1 | 0.033581 | 1.53 | down |
| RP1-8B1.4 | 0.018431 | 1.52 | down |
| RP11-429J17.8 | 0.001638 | 1.52 | down |
| RP5-1139B12.2 | 0.038320 | 1.52 | down |
| RP11-498D10.6 | 0.047831 | 1.52 | down |
| RNA147255 | 0.007784 | 1.52 | down |
| RP11-300J18.3 | 0.017230 | 1.52 | down |
| linc-USP14-3 | 0.018924 | 1.52 | down |
| PVT1 | 0.038242 | 1.52 | down |
| RP11-616L12.1 | 0.012798 | 1.52 | down |
| RNA95083 | 0.025453 | 1.51 | down |
| RP11-121M22.1 | 0.030452 | 1.51 | down |
| uc009wka.2 | 0.001762 | 1.51 | down |
| GLIS3-AS1 | 0.026579 | 1.51 | down |
| RP11-173C20.2 | 0.042724 | 1.51 | down |
| EAF1-AS1 | 0.041449 | 1.51 | down |
| int-HOXB3-90 | 0.028825 | 1.51 | down |
| linc-FAM82A1-3 | 0.006759 | 1.50 | down |
| XR_244022.2 | 0.034231 | 1.50 | down |
| linc-FSHB-4 | 0.048349 | 1.50 | down |
| linc-C1orf63-1 | 0.047725 | 1.50 | down |
| RNA147138 | 0.036322 | 1.50 | down |
| RPS6KA2-IT1 | 0.010725 | 1.50 | down |

# Table S2. All DE-mRNAs between hypertensive and control groups

| **DE-mRNAs** | **P-value** | **FC** | **Regulation** |
| --- | --- | --- | --- |
| NFIB | 0.045378 | 1.75 | up |
| HLA-DRB1 | 0.017160 | 1.51 | up |
| ASAP2 | 0.046908 | 1.56 | up |
| PVRL2 | 0.025037 | 1.86 | up |
| MYL5 | 0.030601 | 1.82 | up |
| KLHDC8B | 0.001047 | 1.51 | up |
| lnc-AL353698.1-1 | 0.030099 | 1.61 | up |
| ENKUR | 0.006106 | 2.14 | up |
| SPX | 0.023575 | 1.93 | up |
| ESAM | 0.031275 | 1.55 | up |
| ACRBP | 0.007003 | 1.69 | up |
| CD36 | 0.007798 | 1.54 | up |
| NENF | 0.011608 | 1.66 | up |
| TMEM40 | 0.003748 | 1.80 | up |
| HLA-DQB1 | 0.008088 | 2.13 | up |
| LMNA | 0.004363 | 1.59 | up |
| PCYT1B | 0.000724 | 2.22 | up |
| GP9 | 0.013105 | 1.75 | up |
| INMT | 0.016317 | 1.68 | up |
| LOC101927412 | 0.026487 | 1.58 | up |
| GPX1 | 0.015611 | 1.63 | up |
| CCL3 | 0.027285 | 2.02 | up |
| CTSA | 0.009109 | 1.51 | up |
| KCND3 | 0.006281 | 2.27 | up |
| MFAP3L | 0.001327 | 1.99 | up |
| TNS1 | 0.004687 | 2.21 | up |
| CENPW | 0.041915 | 1.50 | up |
| RAB30 | 0.016922 | 1.55 | up |
| PDE3A | 0.020431 | 1.66 | up |
| OR2L5 | 0.030834 | 2.59 | up |
| RASGRP3 | 0.018020 | 1.85 | up |
| FRMD3 | 0.002711 | 1.94 | up |
| HDAC9 | 0.008770 | 1.57 | up |
| PGRMC1 | 0.006462 | 1.82 | up |
| SPARC | 0.013885 | 2.03 | up |
| SCGB1C1 | 0.027679 | 2.00 | up |
| KCNG1 | 0.031766 | 2.12 | up |
| LOC101930405 | 0.007649 | 2.39 | up |
| LOC101928195 | 0.041849 | 1.55 | up |
| GP1BB | 0.004659 | 1.94 | up |
| ZNF90 | 0.005977 | 1.50 | up |
| VSIG2 | 0.035145 | 1.70 | up |
| PRKG1 | 0.009057 | 1.92 | up |
| BMP6 | 0.000529 | 1.76 | up |
| CDC14B | 0.033046 | 1.57 | up |
| COMMD9 | 0.029371 | 1.52 | up |
| ABCC3 | 0.034507 | 1.74 | up |
| LPAR3 | 0.047433 | 2.43 | up |
| GNGT1 | 0.000697 | 2.11 | up |
| SH3BGRL2 | 0.007600 | 2.05 | up |
| TREML1 | 0.016415 | 1.85 | up |
| lnc-IL6-3 | 0.011986 | 1.65 | up |
| TUBB1 | 0.020153 | 1.65 | up |
| MITF | 0.000142 | 1.89 | up |
| NAPSA | 0.012196 | 1.82 | up |
| BMP7 | 0.044740 | 1.60 | up |
| C10orf11 | 0.013178 | 1.50 | up |
| WASF1 | 0.026174 | 1.55 | up |
| NFIB | 0.037825 | 1.90 | up |
| ZNF438 | 0.010602 | 1.53 | up |
| C6orf25 | 0.047605 | 1.74 | up |
| CMTM5 | 0.031222 | 1.93 | up |
| ATP5E | 0.018245 | 1.68 | up |
| SLC35D2 | 0.014368 | 1.59 | up |
| NUP210L | 0.032545 | 1.76 | up |
| PRKAR1B | 0.027869 | 1.54 | up |
| VAV2 | 0.022901 | 1.69 | up |
| ANK1 | 0.003607 | 1.56 | up |
| ENDOD1 | 0.038322 | 1.59 | up |
| XK | 0.002624 | 2.27 | up |
| LOC102724479 | 0.001805 | 2.04 | up |
| TPTE | 0.023182 | 1.79 | up |
| ETFA | 0.001058 | 1.59 | up |
| LILRB4 | 0.002733 | 1.87 | up |
| FKBP1B | 0.008146 | 1.51 | up |
| ST7 | 0.007206 | 1.50 | up |
| TRIM58 | 0.015044 | 1.61 | up |
| lnc-IL6-3 | 0.028323 | 2.22 | up |
| ARHGAP8 | 0.004343 | 1.98 | up |
| ZFHX3 | 0.018454 | 1.52 | up |
| C6orf25 | 0.025683 | 1.64 | up |
| NRG1 | 0.014464 | 2.42 | up |
| ITGB5 | 0.043021 | 1.52 | up |
| CDC42EP1 | 0.002746 | 2.79 | up |
| lnc-MBL2-3 | 0.005346 | 2.08 | up |
| PRLR | 0.010799 | 1.97 | up |
| SPDYC | 0.005197 | 1.69 | up |
| CABP5 | 0.024805 | 1.76 | up |
| PTGS1 | 0.012334 | 1.78 | up |
| PARD3 | 0.036672 | 1.84 | up |
| ANKRD9 | 0.008280 | 2.00 | up |
| PLSCR4 | 0.037355 | 1.81 | up |
| MSN | 0.001141 | 1.57 | up |
| FAM69B | 0.020683 | 1.74 | up |
| ZNF385D | 0.044919 | 1.93 | up |
| MITF | 0.000700 | 2.02 | up |
| HIST1H2AG | 0.029519 | 1.60 | up |
| IFI27L2 | 0.004549 | 1.66 | up |
| SELP | 0.030374 | 1.96 | up |
| UNC93B1 | 0.001870 | 1.51 | up |
| TLE1 | 0.015961 | 1.61 | up |
| PRTFDC1 | 0.007413 | 1.85 | up |
| XLOC_l2_010908 | 0.027834 | 1.97 | up |
| CARD9 | 0.016815 | 1.64 | up |
| LRRC23 | 0.042763 | 1.51 | up |
| RTN2 | 0.025223 | 1.56 | up |
| TLR7 | 0.022960 | 1.52 | up |
| NAPSA | 0.023790 | 1.64 | up |
| PRDX4 | 0.040397 | 1.56 | up |
| IQSEC2 | 0.020252 | 1.74 | up |
| SLC31A1 | 0.018872 | 1.51 | up |
| MARCO | 0.013524 | 1.86 | up |
| DDX11 | 0.003195 | 1.57 | up |
| DNAH14 | 0.016184 | 1.71 | up |
| PF4 | 0.011746 | 1.53 | up |
| BEND2 | 0.033072 | 2.67 | up |
| ACER2 | 0.016984 | 1.61 | up |
| PKIG | 0.001114 | 2.36 | up |
| LMNTD2 | 0.020940 | 1.60 | up |
| MEIS1 | 0.006038 | 1.59 | up |
| SPAG11B | 0.027207 | 1.81 | up |
| ENKUR | 0.001479 | 2.13 | up |
| CD79B | 0.036229 | 1.94 | up |
| DDX11 | 0.012290 | 2.05 | up |
| JAM3 | 0.045939 | 1.64 | up |
| MZB1 | 0.036699 | 2.57 | up |
| HLF | 0.025529 | 2.23 | up |
| C4orf36 | 0.009033 | 1.57 | up |
| MYL2 | 0.038774 | 1.87 | up |
| PKHD1L1 | 0.036286 | 1.79 | up |
| PTCRA | 0.026275 | 1.62 | up |
| AQP10 | 0.029226 | 2.54 | up |
| UTS2 | 0.003802 | 7.41 | up |
| FHL2 | 0.000747 | 2.29 | up |
| HIST2H2BE | 0.016228 | 1.56 | up |
| MITF | 0.000179 | 1.59 | up |
| HIST1H3H | 0.001618 | 1.76 | up |
| IGLL1 | 0.037491 | 2.22 | up |
| LGALS3 | 0.027811 | 1.58 | up |
| POU2AF1 | 0.037070 | 1.73 | up |
| TUBB6 | 0.014251 | 1.90 | up |
| UTS2 | 0.007037 | 4.08 | up |
| ABCC3 | 0.032193 | 1.84 | up |
| PDLIM1 | 0.003946 | 1.65 | up |
| BLNK | 0.049726 | 1.59 | up |
| RILP | 0.034639 | 1.54 | up |
| PBX1 | 0.004957 | 2.03 | up |
| ARL15 | 0.030218 | 1.55 | up |
| OR2L3 | 0.025360 | 1.70 | up |
| TPM1 | 0.030716 | 1.60 | up |
| lnc-GTF2E2-1 | 0.005040 | 1.56 | up |
| HIST1H2BK | 0.000802 | 1.66 | up |
| ALOX12 | 0.049760 | 1.83 | up |
| CAV2 | 0.000969 | 3.32 | up |
| LOC102724332 | 0.009556 | 2.20 | up |
| CLEC1B | 0.041238 | 1.76 | up |
| BLK | 0.027773 | 1.72 | up |
| lnc-PDZD8-1 | 0.049938 | 1.56 | up |
| GNG11 | 0.017148 | 1.86 | up |
| LOC102725284 | 0.012635 | 1.69 | up |
| NT5M | 0.047408 | 1.58 | up |
| WDFY4 | 0.023745 | 1.56 | up |
| CRAT | 0.004256 | 1.69 | up |
| SLC3A1 | 0.002491 | 1.98 | up |
| ZMYND12 | 0.002109 | 1.70 | up |
| LOC101928932 | 0.039020 | 2.31 | up |
| TSPAN4 | 0.020797 | 1.53 | up |
| PNOC | 0.049988 | 1.57 | up |
| SH3TC2 | 0.009202 | 2.14 | up |
| PPP1R14A | 0.002595 | 1.79 | up |
| IGLL5 | 0.041145 | 2.16 | up |
| DNM3 | 0.000732 | 2.18 | up |
| SH3BGRL2 | 0.015202 | 1.92 | up |
| TMEM144 | 0.020337 | 1.58 | up |
| KLHL14 | 0.004403 | 2.58 | up |
| H2AFJ | 0.007345 | 1.53 | up |
| TFPI | 0.006854 | 1.89 | up |
| CCL3L3 | 0.023307 | 1.99 | up |
| HOMER3 | 0.007243 | 1.53 | up |
| ATF7 | 0.013009 | 1.61 | up |
| GSTO1 | 0.040829 | 1.53 | up |
| FSCN1 | 0.015154 | 1.76 | up |
| SNCA | 0.015074 | 1.78 | up |
| RNF208 | 0.007624 | 1.69 | up |
| LEPROT | 0.011210 | 1.51 | up |
| STAB1 | 0.011426 | 1.76 | up |
| HLA-DQA1 | 0.011580 | 2.18 | up |
| TFPI | 0.035672 | 1.91 | up |
| LTBR | 0.026937 | 1.52 | up |
| lnc-CHADL-1 | 0.022549 | 1.67 | up |
| KLHL6 | 0.003480 | 1.91 | up |
| PDGFA | 0.031571 | 1.72 | up |
| LAMTOR1 | 0.005083 | 1.52 | up |
| NCK1-AS1 | 0.002866 | 1.57 | up |
| MEIS1 | 0.005240 | 1.70 | up |
| PRKAR2B | 0.042983 | 1.68 | up |
| LOC101929911 | 0.028223 | 1.72 | up |
| RAB32 | 0.042595 | 1.55 | up |
| RHCE | 0.039267 | 1.67 | up |
| MPL | 0.020066 | 1.66 | up |
| NT5C3A | 0.017236 | 1.61 | up |
| ZNF185 | 0.003988 | 1.52 | up |
| lnc-MBL2-3 | 0.006593 | 2.15 | up |
| GP1BB | 0.004614 | 2.02 | up |
| PBXIP1 | 0.014942 | 1.53 | up |
| CAV1 | 0.035519 | 1.70 | up |
| LOC100131508 | 0.005691 | 2.06 | up |
| ENKUR | 0.000947 | 2.43 | up |
| DNAH6 | 0.038139 | 2.67 | up |
| RAD23B | 0.022113 | 1.54 | up |
| LIMS1 | 0.039648 | 1.55 | up |
| HIST1H2AC | 0.039900 | 1.61 | up |
| FHL2 | 0.002898 | 1.55 | up |
| THSD1 | 0.013911 | 1.55 | up |
| MEIS1 | 0.003536 | 2.35 | up |
| HLA-DRB5 | 0.043434 | 2.44 | up |
| CTTN | 0.032901 | 1.54 | up |
| MAPK10 | 0.003439 | 1.86 | up |
| PRDX6 | 0.007939 | 1.52 | up |
| SMIM5 | 0.007396 | 1.98 | up |
| PTPRO | 0.021599 | 1.50 | up |
| NAT8B | 0.011514 | 1.72 | up |
| TPM1 | 0.028399 | 1.57 | up |
| LY6G6F | 0.046296 | 1.63 | up |
| SH2B2 | 0.004569 | 1.60 | up |
| TBC1D12 | 0.025348 | 1.95 | up |
| LOC729451 | 0.014760 | 2.03 | up |
| XLOC_l2_013153 | 0.022120 | 2.09 | up |
| CXCL5 | 0.005322 | 1.58 | up |
| TMPRSS9 | 0.020011 | 1.57 | up |
| PTGS1 | 0.020017 | 1.82 | up |
| ST3GAL3 | 0.026343 | 1.59 | up |
| TM4SF1 | 0.029525 | 1.89 | up |
| SEPT10 | 0.001595 | 1.79 | up |
| ARMCX6 | 0.003127 | 1.66 | up |
| NFIB | 0.042037 | 1.76 | up |
| LOC338694 | 0.012929 | 1.53 | up |
| ZAK | 0.015363 | 1.50 | up |
| EHD3 | 0.016687 | 1.69 | up |
| SIAE | 0.022485 | 1.50 | up |
| DCBLD2 | 0.018134 | 1.79 | up |
| TTLL7 | 0.004413 | 1.64 | up |
| MYDGF | 0.011578 | 1.67 | up |
| GNG7 | 0.011768 | 1.56 | up |
| FRMD3 | 0.000149 | 2.03 | up |
| RNF11 | 0.033066 | 1.51 | up |
| NRP2 | 0.010642 | 2.04 | up |
| ARMC3 | 0.006459 | 1.71 | up |
| PEAR1 | 0.020327 | 2.06 | up |
| TK2 | 0.032828 | 1.51 | up |
| SLC17A9 | 0.009305 | 1.52 | up |
| HSPB1 | 0.020324 | 1.52 | up |
| ADI1 | 0.004756 | 1.56 | up |
| PRSS21 | 0.011571 | 2.37 | up |
| KIAA1598 | 0.024206 | 1.81 | up |
| C10orf25 | 0.035985 | 1.57 | up |
| SDPR | 0.008914 | 1.98 | up |
| NRG1 | 0.012431 | 2.07 | up |
| NGFRAP1 | 0.003332 | 1.92 | up |
| C19orf18 | 0.026474 | 1.69 | up |
| TBXA2R | 0.007224 | 1.69 | up |
| PKIG | 0.013259 | 1.82 | up |
| MMD | 0.003225 | 1.82 | up |
| FCRL1 | 0.029783 | 1.69 | up |
| TUBA8 | 0.012133 | 1.65 | up |
| CLU | 0.027908 | 1.77 | up |
| GPX1 | 0.003322 | 1.87 | up |
| C2orf88 | 0.021384 | 1.68 | up |
| IGJ | 0.031267 | 2.85 | up |
| RNF208 | 0.002313 | 2.27 | up |
| MYL9 | 0.033248 | 1.62 | up |
| TUBB2A | 0.006616 | 1.82 | up |
| PPDPF | 0.033356 | 1.52 | up |
| TMEM150C | 0.038158 | 1.64 | up |
| EMP1 | 0.002832 | 2.07 | up |
| ARHGAP6 | 0.025576 | 1.85 | up |
| PBX1 | 0.040886 | 2.26 | up |
| COLCA2 | 0.025169 | 3.06 | up |
| RNF208 | 0.002972 | 2.37 | up |
| MTURN | 0.004749 | 1.92 | up |
| MSN | 0.003711 | 1.76 | up |
| NAT8B | 0.005369 | 1.85 | up |
| HGD | 0.000644 | 2.06 | up |
| MPP1 | 0.005884 | 1.74 | up |
| RAB27B | 0.016607 | 1.67 | up |
| ATF3 | 0.017882 | 1.83 | up |
| GRAP2 | 0.007751 | 1.58 | up |
| DENND5B | 0.001518 | 1.99 | up |
| ZNF768 | 0.002236 | 1.61 | up |
| F13A1 | 0.010806 | 1.83 | up |
| HIST1H2AC | 0.010307 | 1.84 | up |
| SMOX | 0.008232 | 1.62 | up |
| BEND2 | 0.019715 | 1.56 | up |
| ARHGAP6 | 0.005391 | 1.77 | up |
| RASGRP3 | 0.018333 | 1.83 | up |
| BLNK | 0.034910 | 1.63 | up |
| RGS10 | 0.002071 | 1.79 | up |
| PCYT1B | 0.004384 | 2.38 | up |
| NCKAP1 | 0.028983 | 1.71 | up |
| DAPP1 | 0.000001 | 1.75 | up |
| PCDH9 | 0.043942 | 2.16 | up |
| UGT3A1 | 0.017659 | 1.50 | up |
| MCUR1 | 0.018143 | 1.51 | up |
| GSTA5 | 0.030421 | 1.89 | up |
| MPL | 0.012577 | 2.04 | up |
| OR52N4 | 0.049148 | 1.57 | up |
| VWF | 0.016637 | 1.63 | up |
| HIST1H2AI | 0.001848 | 2.00 | up |
| LGALS2 | 0.019311 | 3.02 | up |
| FHL1 | 0.045791 | 1.61 | up |
| CCDC149 | 0.021652 | 1.82 | up |
| PVALB | 0.009248 | 1.55 | up |
| PPAPDC1B | 0.023327 | 1.51 | up |
| LOC102724810 | 0.010085 | 2.18 | up |
| MGLL | 0.048070 | 1.67 | up |
| lnc-TNKS2-1 | 0.017780 | 1.62 | up |
| PTGER3 | 0.001913 | 1.93 | up |
| SAMD14 | 0.036667 | 1.58 | up |
| F13A1 | 0.013951 | 1.77 | up |
| GMPR | 0.006395 | 1.78 | up |
| INTU | 0.000032 | 1.98 | up |
| TDRP | 0.005799 | 2.37 | up |
| FRMD3 | 0.024270 | 1.52 | up |
| PDGFC | 0.007597 | 1.70 | up |
| SUCNR1 | 0.019761 | 1.94 | up |
| GUCY1B3 | 0.016360 | 1.96 | up |
| SNN | 0.019554 | 1.50 | up |
| ALCAM | 0.007069 | 1.65 | up |
| MAP3K7CL | 0.013461 | 1.88 | up |
| RUFY1 | 0.044129 | 1.62 | up |
| MTURN | 0.020387 | 1.75 | up |
| TTLL7 | 0.004136 | 1.52 | up |
| RGS6 | 0.022707 | 1.58 | up |
| CDKN1A | 0.000029 | 1.96 | up |
| ZAK | 0.001603 | 1.73 | up |
| MFAP3L | 0.019465 | 1.55 | up |
| MT1X | 0.011104 | 1.68 | up |
| CATSPER1 | 0.030600 | 1.54 | up |
| GFI1B | 0.004790 | 1.62 | up |
| lnc-SH3BGRL2-1 | 0.048010 | 1.78 | up |
| WASF1 | 0.010040 | 1.89 | up |
| ZNF532 | 0.044454 | 1.62 | up |
| AF176921 | 0.036033 | 2.37 | up |
| ENST00000558425 | 0.013035 | 2.00 | up |
| ENST00000455737 | 0.012194 | 2.51 | up |
| ENST00000383417 | 0.039501 | 2.00 | up |
| ENST00000390323 | 0.018978 | 2.23 | up |
| ENST00000390626 | 0.027981 | 2.77 | up |
| ENST00000390604 | 0.013938 | 2.39 | up |
| BX107688 | 0.012077 | 1.59 | up |
| ENST00000603660 | 0.008942 | 1.95 | up |
| ENST00000390593 | 0.035668 | 2.60 | up |
| XR_426087 | 0.043161 | 1.91 | up |
| ENST00000390605 | 0.008389 | 2.47 | up |
| ENST00000390622 | 0.013044 | 2.41 | up |
| ENST00000390610 | 0.010239 | 2.98 | up |
| ENST00000390615 | 0.004982 | 2.22 | up |
| ENST00000570121 | 0.020144 | 1.95 | up |
| ENST00000390278 | 0.045526 | 2.08 | up |
| ENST00000338912 | 0.042771 | 1.74 | up |
| ENST00000390247 | 0.036237 | 2.14 | up |
| AF194718 | 0.024778 | 2.59 | up |
| ENST00000434710 | 0.007386 | 2.10 | up |
| ENST00000390323 | 0.024966 | 2.16 | up |
| KJ487098 | 0.045100 | 1.86 | up |
| ENST00000607355 | 0.006881 | 1.55 | up |
| ENST00000390628 | 0.018079 | 1.91 | up |
| ENST00000425181 | 0.010353 | 2.26 | up |
| ENST00000390252 | 0.038448 | 2.30 | up |
| ENST00000390268 | 0.017613 | 1.61 | up |
| ENST00000468879 | 0.043297 | 1.90 | up |
| ENST00000390616 | 0.014707 | 2.33 | up |
| ENST00000390636 | 0.006824 | 2.32 | up |
| ENST00000562905 | 0.009186 | 2.38 | up |
| ENST00000390625 | 0.013935 | 2.10 | up |
| AK025118 | 0.033168 | 1.70 | up |
| ENST00000390547 | 0.043644 | 2.60 | up |
| ENST00000410078 | 0.026010 | 2.09 | up |
| ENST00000390256 | 0.001512 | 2.25 | up |
| ENST00000471857 | 0.038209 | 2.38 | up |
| ENST00000383418 | 0.046421 | 1.91 | up |
| ENST00000390271 | 0.023588 | 2.71 | up |
| BF175071 | 0.044430 | 2.63 | up |
| ENST00000390621 | 0.003936 | 2.92 | up |
| DQ098707 | 0.033603 | 2.12 | up |
| ENST00000390603 | 0.007388 | 2.23 | up |
| ENST00000390283 | 0.006477 | 2.82 | up |
| ENST00000453673 | 0.041525 | 1.83 | up |
| ENST00000604106 | 0.014712 | 2.16 | up |
| ENST00000483158 | 0.042930 | 2.29 | up |
| ENST00000492167 | 0.037433 | 2.25 | up |
| DA142060 | 0.010160 | 1.54 | up |
| ENST00000424969 | 0.008805 | 2.06 | up |
| ENST00000390606 | 0.026767 | 1.88 | up |
| ENST00000390632 | 0.004697 | 2.23 | up |
| ENST00000492446 | 0.019719 | 2.49 | up |
| ENST00000390243 | 0.024941 | 2.02 | up |
| AJ252276 | 0.004924 | 2.93 | up |
| ENST00000390632 | 0.007434 | 2.25 | up |
| ENST00000390617 | 0.009715 | 2.32 | up |
| THC2497807 | 0.009897 | 1.52 | up |
| ENST00000390556 | 0.028614 | 2.11 | up |
| OR10G2 | 0.037584 | 1.52 | down |
| ERRFI1 | 0.002983 | 1.63 | down |
| PLAC8 | 0.015974 | 1.55 | down |
| EFR3B | 0.023133 | 1.64 | down |
| LINC00944 | 0.035159 | 1.66 | down |
| BCKDHB | 0.007169 | 1.57 | down |
| SRSF12 | 0.004644 | 2.13 | down |
| LOC101927095 | 0.021889 | 1.65 | down |
| FAM231A | 0.001827 | 1.54 | down |
| PDE3B | 0.046939 | 1.55 | down |
| KCNE5 | 0.009542 | 1.71 | down |
| SCARNA23 | 0.019241 | 1.54 | down |
| N4BP2L1 | 0.043605 | 1.56 | down |
| HSD17B7 | 0.008185 | 1.68 | down |
| lnc-C1orf31-1 | 0.017124 | 1.69 | down |
| APOLD1 | 0.001040 | 1.80 | down |
| LOC642943 | 0.021674 | 1.55 | down |
| lnc-CX3CR1-2 | 0.004326 | 1.62 | down |
| XLOC_l2_003882 | 0.006051 | 1.51 | down |
| PIGK | 0.032232 | 1.58 | down |
| PARD3B | 0.024899 | 3.47 | down |
| GPR160 | 0.047903 | 1.52 | down |
| FAM122C | 0.003131 | 1.55 | down |
| HDDC2 | 0.044441 | 1.62 | down |
| LOC101928669 | 0.016313 | 1.78 | down |
| ZNF780A | 0.006250 | 1.51 | down |
| C15orf53 | 0.033329 | 1.64 | down |
| ZNF239 | 0.002765 | 1.68 | down |
| PDCD6 | 0.044418 | 1.55 | down |
| CCDC83 | 0.008363 | 2.46 | down |
| CCDC37 | 0.013447 | 1.69 | down |
| SPATA13 | 0.036789 | 1.50 | down |
| TSGA10 | 0.049802 | 1.57 | down |
| CCDC13 | 0.024722 | 1.53 | down |
| MDFIC | 0.048324 | 1.75 | down |
| RPH3A | 0.026973 | 1.77 | down |
| lnc-RP3-377D14.1.1-3 | 0.028106 | 1.82 | down |
| GDAP1L1 | 0.020735 | 1.77 | down |
| lnc-BAZ1A-1 | 0.037240 | 1.71 | down |
| ANKRD18A | 0.004413 | 1.71 | down |
| PEBP4 | 0.000722 | 1.62 | down |
| KIF3A | 0.003168 | 1.70 | down |
| ELOVL6 | 0.020335 | 1.63 | down |
| C5orf66-AS1 | 0.013289 | 1.57 | down |
| PLGLB1 | 0.002574 | 1.97 | down |
| NPAS2 | 0.043374 | 1.86 | down |
| PLXDC1 | 0.009301 | 2.27 | down |
| MYO5B | 0.019860 | 1.64 | down |
| ZC3H13 | 0.014184 | 1.54 | down |
| APOLD1 | 0.020756 | 1.83 | down |
| ADAMTS17 | 0.016996 | 1.61 | down |
| DLX1 | 0.043580 | 1.54 | down |
| INTS6 | 0.039531 | 1.75 | down |
| lnc-FAM84B-1 | 0.021788 | 1.65 | down |
| CREBZF | 0.011584 | 1.66 | down |
| AVIL | 0.024804 | 1.87 | down |
| CPB2 | 0.044674 | 2.16 | down |
| FGFR1 | 0.011713 | 1.71 | down |
| GPR114 | 0.022596 | 1.61 | down |
| LOC100507144 | 0.022346 | 1.73 | down |
| FAM47A | 0.011034 | 1.69 | down |
| AKTIP | 0.021101 | 1.50 | down |
| DPAGT1 | 0.026955 | 1.77 | down |
| PLXDC1 | 0.043719 | 1.65 | down |
| ZFP42 | 0.027645 | 1.58 | down |
| RPH3A | 0.010278 | 2.26 | down |
| SLC26A3 | 0.049549 | 1.70 | down |
| AKR1C1 | 0.011071 | 1.75 | down |
| AKTIP | 0.005203 | 1.53 | down |
| HOXB2 | 0.036762 | 1.62 | down |
| LOC100996741 | 0.004934 | 1.70 | down |
| FAM178A | 0.029385 | 1.80 | down |
| ZMYND10 | 0.002079 | 1.51 | down |
| SCGB1D2 | 0.016872 | 1.65 | down |
| OLFM2 | 0.009626 | 1.83 | down |
| TAS2R60 | 0.013719 | 1.72 | down |
| FAM200B | 0.021645 | 1.55 | down |
| GTSCR1 | 0.045155 | 2.31 | down |
| NOL4L | 0.024539 | 1.65 | down |
| FASLG | 0.048319 | 1.57 | down |
| OR5B21 | 0.023385 | 1.52 | down |
| SLC14A2 | 0.017918 | 1.53 | down |
| POT1 | 0.014810 | 1.53 | down |
| OR5B12 | 0.032165 | 2.07 | down |
| TRAPPC10 | 0.036328 | 1.52 | down |
| LOC100128670 | 0.013317 | 1.67 | down |
| PLGLB1 | 0.034471 | 1.50 | down |
| lnc-SNX20-2 | 0.031586 | 1.62 | down |
| OR6N1 | 0.045800 | 2.16 | down |
| KLHL32 | 0.021154 | 1.56 | down |
| TAS2R41 | 0.007589 | 2.30 | down |
| lnc-PPIAL4G-4 | 0.015561 | 1.69 | down |
| TLR3 | 0.018785 | 2.04 | down |
| SPATA9 | 0.026268 | 1.89 | down |
| ZNF706 | 0.046524 | 1.54 | down |
| TRMT13 | 0.024607 | 1.52 | down |
| lnc-AC092031.1-1 | 0.029930 | 1.65 | down |
| THC2648833 | 0.041018 | 1.61 | down |
| AK094554 | 0.031839 | 1.75 | down |
| XR_158882 | 0.048206 | 1.66 | down |
| ENST00000456741 | 0.031903 | 1.71 | down |
| ENST00000431580 | 0.042764 | 1.56 | down |
| AK024925 | 0.000134 | 1.53 | down |
| AK127758 | 0.022190 | 1.59 | down |
| ENST00000617347 | 0.034523 | 1.56 | down |
| ENST00000390432 | 0.024763 | 1.52 | down |
| THC2710827 | 0.006823 | 1.93 | down |
| ENST00000390460 | 0.025388 | 1.80 | down |
| THC2715287 | 0.019698 | 1.54 | down |

# Table S3. lncRNA-mRNA co-expression pairs

| **lncRNA** | **mRNA** | **Correlation** | P_value |
| --- | --- | --- | --- |
| TCONS_00012168 | ASAP2 | 0.913754397 | 3.25E-05 |
| ENST00000420595.1 | ASAP2 | 0.934548164 | 8.47E-06 |
| RNA95016\|RNS_98_196 | PVRL2 | -0.914168747 | 3.17E-05 |
| ENST00000561134.1 | MYL5 | 0.986621179 | 3.30E-09 |
| HIT000078556_03 | A_23_P343900 | 0.969675527 | 1.92E-07 |
| uc021thc.2 | A_23_P343900 | 0.910438334 | 3.90E-05 |
| XR_429426.1 | A_23_P343900 | 0.908776823 | 4.26E-05 |
| XR_429429.1 | A_23_P343900 | 0.904966647 | 5.19E-05 |
| NR_024586.1 | PLAC8 | 0.904976657 | 5.19E-05 |
| ENST00000552061.1 | PLAC8 | 0.908389036 | 4.35E-05 |
| NR_003703.1 | KLHDC8B | -0.90108912 | 6.30E-05 |
| uc021thc.2 | A_33_P3332982 | 0.925529406 | 1.59E-05 |
| TCONS_00020976 | LINC00944 | 0.918377561 | 2.48E-05 |
| TCONS_00017180 | lnc-AL353698.1-1 | 0.986604981 | 3.32E-09 |
| NR_027145.2 | ENKUR | 0.947856225 | 2.78E-06 |
| ENST00000467896.2 | ENKUR | 0.912468043 | 3.49E-05 |
| ENST00000420595.1 | ENKUR | 0.911625692 | 3.65E-05 |
| ENST00000429328.2 | ENKUR | 0.920129347 | 2.24E-05 |
| uc004ehp.2 | ENKUR | 0.902852272 | 5.78E-05 |
| ENST00000555918.1 | SPX | 0.907247599 | 4.62E-05 |
| ENST00000558120.1 | SPX | 0.918565882 | 2.46E-05 |
| NR_027145.2 | SPX | 0.9114939 | 3.68E-05 |
| TCONS_00018467 | SPX | 0.916977182 | 2.70E-05 |
| XR_429366.1 | SPX | 0.907561468 | 4.54E-05 |
| TCONS_00018641 | SPX | 0.909122988 | 4.18E-05 |
| uc004ehp.2 | SPX | 0.929019215 | 1.26E-05 |
| uc022caj.1 | SPX | 0.932913192 | 9.56E-06 |
| TCONS_00022516 | SPX | 0.915430832 | 2.95E-05 |
| ENST00000578583.1 | SRSF12 | 0.926096866 | 1.53E-05 |
| uc009wka.2 | SRSF12 | 0.905825865 | 4.97E-05 |
| ENST00000609413.1 | SRSF12 | 0.911478165 | 3.68E-05 |
| RNA147577\|p0681_imsncRNA442 | SRSF12 | 0.927138265 | 1.43E-05 |
| NR_110099.1 | SRSF12 | 0.920243588 | 2.22E-05 |
| ENST00000424245.2 | SRSF12 | 0.90870426 | 4.28E-05 |
| ENST00000535911.1 | SRSF12 | 0.90269842 | 5.82E-05 |
| TCONS_00005965 | SRSF12 | -0.900298401 | 6.55E-05 |
| ENST00000416310.1 | SRSF12 | 0.926092003 | 1.53E-05 |
| ENST00000432411.1 | SRSF12 | 0.916467303 | 2.78E-05 |
| ENST00000558120.1 | ESAM | 0.90956006 | 4.09E-05 |
| XR_241556.1 | ESAM | 0.937456384 | 6.78E-06 |
| ENST00000561134.1 | ESAM | 0.94190392 | 4.73E-06 |
| ENST00000420595.1 | ESAM | 0.915140965 | 3.00E-05 |
| XR_429366.1 | ESAM | 0.921716254 | 2.03E-05 |
| ENST00000602325.1 | ESAM | 0.91309618 | 3.37E-05 |
| ENST00000424116.2 | ESAM | 0.940589881 | 5.27E-06 |
| ENST00000607175.1 | ESAM | 0.907030473 | 4.67E-05 |
| TCONS_00022516 | ESAM | 0.935949504 | 7.62E-06 |
| TCONS_00012168 | ACRBP | 0.906971485 | 4.69E-05 |
| NR_027145.2 | ACRBP | 0.929571462 | 1.21E-05 |
| ENST00000608442.1 | ACRBP | 0.911239157 | 3.73E-05 |
| ENST00000467896.2 | ACRBP | 0.904955528 | 5.20E-05 |
| ENST00000420595.1 | ACRBP | 0.964079659 | 4.43E-07 |
| TCONS_00018335 | ACRBP | 0.939265776 | 5.87E-06 |
| LIT2094 | ACRBP | 0.918317775 | 2.49E-05 |
| uc004ehp.2 | ACRBP | 0.941204216 | 5.01E-06 |
| ENST00000424116.2 | ACRBP | 0.900583409 | 6.46E-05 |
| ENST00000607175.1 | ACRBP | 0.909397681 | 4.12E-05 |
| uc022caj.1 | ACRBP | 0.947157977 | 2.97E-06 |
| ENST00000558120.1 | CD36 | 0.916943181 | 2.70E-05 |
| NR_037928.1 | CD36 | 0.935944075 | 7.62E-06 |
| ENST00000560924.1 | CD36 | 0.934853181 | 8.28E-06 |
| TCONS_00016405 | A_21_P0006091 | 0.91184608 | 3.61E-05 |
| HIT000078556_03 | A_24_P144346 | 0.958954855 | 8.56E-07 |
| uc021thc.2 | A_24_P144346 | 0.953668553 | 1.56E-06 |
| XR_429426.1 | A_24_P144346 | 0.969008047 | 2.14E-07 |
| XR_429429.1 | A_24_P144346 | 0.975349134 | 6.88E-08 |
| uc002sti.1 | A_24_P144346 | 0.925759213 | 1.57E-05 |
| ENST00000605136.1 | NENF | -0.921252385 | 2.09E-05 |
| ENST00000511279.1 | LOC101927095 | 0.923010051 | 1.87E-05 |
| ENST00000448017.1 | TMEM40 | 0.909416375 | 4.12E-05 |
| NR_027145.2 | TMEM40 | 0.951569317 | 1.93E-06 |
| ENST00000447323.1 | TMEM40 | 0.916950284 | 2.70E-05 |
| ENST00000467896.2 | TMEM40 | 0.920462621 | 2.19E-05 |
| ENST00000443523.1 | TMEM40 | 0.901361543 | 6.22E-05 |
| ENST00000607175.1 | TMEM40 | 0.902407255 | 5.91E-05 |
| XR_427724.1 | TMEM40 | 0.919229392 | 2.36E-05 |
| ENST00000443574.1 | HLA-DQB1 | 0.939673156 | 5.68E-06 |
| HIT000078556_03 | A_24_P367432 | 0.941175459 | 5.02E-06 |
| uc021thc.2 | A_24_P367432 | 0.907507093 | 4.56E-05 |
| XR_429426.1 | A_24_P367432 | 0.976279551 | 5.68E-08 |
| NR_026800.1 | A_24_P367432 | 0.928038628 | 1.35E-05 |
| XR_429429.1 | A_24_P367432 | 0.93906334 | 5.97E-06 |
| uc021vkt.1 | A_24_P367432 | 0.903935379 | 5.47E-05 |
| ENST00000606593.1 | KCNE5 | 0.93558684 | 7.83E-06 |
| ENST00000431928.1 | SCARNA23 | 0.909536353 | 4.09E-05 |
| ENST00000555918.1 | LMNA | 0.967592296 | 2.67E-07 |
| ENST00000558120.1 | LMNA | 0.955815964 | 1.23E-06 |
| ENST00000552502.1 | LMNA | -0.919063736 | 2.38E-05 |
| NR_027145.2 | LMNA | 0.940654187 | 5.25E-06 |
| TCONS_00018467 | LMNA | 0.938855167 | 6.07E-06 |
| NR_037928.1 | LMNA | 0.90690802 | 4.70E-05 |
| ENST00000560924.1 | LMNA | 0.958638015 | 8.89E-07 |
| uc004ehp.2 | LMNA | 0.912230408 | 3.53E-05 |
| uc022caj.1 | LMNA | 0.930903657 | 1.10E-05 |
| TCONS_00022516 | LMNA | 0.901276858 | 6.24E-05 |
| ENST00000430228.1 | A_33_P3482466 | 0.91759644 | 2.60E-05 |
| ENST00000609413.1 | A_33_P3482466 | 0.90452163 | 5.31E-05 |
| RNA147577\|p0681_imsncRNA442 | A_33_P3482466 | 0.930486572 | 1.14E-05 |
| ENST00000586885.1 | A_33_P3482466 | 0.900347214 | 6.53E-05 |
| TCONS_00024308 | A_33_P3482466 | 0.940540676 | 5.29E-06 |
| uc002tzb.1 | A_33_P3482466 | 0.902309189 | 5.94E-05 |
| XR_429339.1 | A_33_P3482466 | 0.907490851 | 4.56E-05 |
| ENST00000430694.1 | A_33_P3274678 | 0.901572941 | 6.15E-05 |
| NR_026800.1 | A_33_P3274678 | 0.904616309 | 5.29E-05 |
| TCONS_00016405 | A_33_P3274678 | -0.926124146 | 1.53E-05 |
| HIT000078556_03 | A_24_P204727 | 0.968585639 | 2.29E-07 |
| uc021thc.2 | A_24_P204727 | 0.960202199 | 7.35E-07 |
| XR_429426.1 | A_24_P204727 | 0.977639441 | 4.24E-08 |
| XR_429429.1 | A_24_P204727 | 0.982583805 | 1.23E-08 |
| uc002sti.1 | A_24_P204727 | 0.93731497 | 6.86E-06 |
| ENST00000592381.1 | N4BP2L1 | 0.905575444 | 5.04E-05 |
| TCONS_00026998 | N4BP2L1 | 0.946663125 | 3.11E-06 |
| NR_024586.1 | N4BP2L1 | 0.935666546 | 7.79E-06 |
| XR_246176.1 | N4BP2L1 | 0.92785692 | 1.36E-05 |
| ENST00000555918.1 | PCYT1B | 0.902489937 | 5.88E-05 |
| ENST00000608505.1 | PCYT1B | 0.909798171 | 4.04E-05 |
| NR_027145.2 | PCYT1B | 0.95278215 | 1.71E-06 |
| ENST00000447323.1 | PCYT1B | 0.93628979 | 7.42E-06 |
| ENST00000554253.1 | PCYT1B | 0.915983271 | 2.86E-05 |
| ENST00000445429.1 | PCYT1B | 0.916100461 | 2.84E-05 |
| ENST00000467896.2 | PCYT1B | 0.908869607 | 4.24E-05 |
| ENST00000553317.1 | PCYT1B | 0.932340313 | 9.96E-06 |
| ENST00000457661.1 | PCYT1B | 0.95093931 | 2.06E-06 |
| ENST00000556936.1 | PCYT1B | 0.911225607 | 3.74E-05 |
| ENST00000430620.1 | PCYT1B | 0.939892416 | 5.58E-06 |
| ENST00000607175.1 | PCYT1B | 0.914599416 | 3.10E-05 |
| XR_427724.1 | PCYT1B | 0.953460894 | 1.59E-06 |
| ENST00000558120.1 | GP9 | 0.920234939 | 2.22E-05 |
| XR_241556.1 | GP9 | 0.935328063 | 7.99E-06 |
| NR_027145.2 | GP9 | 0.921637703 | 2.04E-05 |
| ENST00000608442.1 | GP9 | 0.908143347 | 4.41E-05 |
| ENST00000561134.1 | GP9 | 0.91654822 | 2.77E-05 |
| ENST00000420595.1 | GP9 | 0.9405087 | 5.31E-06 |
| TCONS_00018335 | GP9 | 0.905218119 | 5.13E-05 |
| ENST00000442008.2 | GP9 | 0.909091481 | 4.19E-05 |
| ENST00000602325.1 | GP9 | 0.944855242 | 3.66E-06 |
| LIT2094 | GP9 | 0.938179525 | 6.41E-06 |
| NR_037928.1 | GP9 | 0.945130277 | 3.57E-06 |
| ENST00000424116.2 | GP9 | 0.962114822 | 5.77E-07 |
| ENST00000607175.1 | GP9 | 0.944718073 | 3.70E-06 |
| TCONS_00022516 | GP9 | 0.908482548 | 4.33E-05 |
| ENST00000442008.2 | INMT | 0.922444891 | 1.94E-05 |
| LIT2094 | INMT | 0.916524433 | 2.77E-05 |
| ENST00000608241.1 | INMT | 0.920539864 | 2.18E-05 |
| XR_246348.2 | LOC101927412 | 0.986994441 | 2.87E-09 |
| ENST00000443565.1 | LOC101927412 | 0.987709238 | 2.16E-09 |
| ENST00000558120.1 | GPX1 | 0.92421858 | 1.73E-05 |
| XR_241556.1 | GPX1 | 0.939429395 | 5.80E-06 |
| NR_027145.2 | GPX1 | 0.90018302 | 6.59E-05 |
| ENST00000442008.2 | GPX1 | 0.921377519 | 2.07E-05 |
| LIT2094 | GPX1 | 0.955298638 | 1.30E-06 |
| NR_037928.1 | GPX1 | 0.979207063 | 2.96E-08 |
| ENST00000432668.1 | GPX1 | -0.905163157 | 5.14E-05 |
| ENST00000424116.2 | GPX1 | 0.922370336 | 1.95E-05 |
| TCONS_00024392 | GPX1 | -0.917221781 | 2.66E-05 |
| ENST00000439622.1 | HSD17B7 | 0.900204742 | 6.58E-05 |
| TCONS_00026998 | HSD17B7 | 0.903084518 | 5.71E-05 |
| XR_246176.1 | HSD17B7 | 0.910075582 | 3.98E-05 |
| ENST00000426704.1 | HSD17B7 | 0.936125474 | 7.52E-06 |
| ENST00000413818.2 | HSD17B7 | 0.922494855 | 1.93E-05 |
| XR_428131.1 | HSD17B7 | 0.902856651 | 5.78E-05 |
| uc001ttj.1 | HSD17B7 | 0.910000654 | 3.99E-05 |
| NR_037928.1 | CTSA | 0.944434724 | 3.80E-06 |
| ENST00000424116.2 | CTSA | 0.925029368 | 1.64E-05 |
| ENST00000607314.1 | A_33_P3392192 | 0.996037145 | 7.65E-12 |
| HIT000078556_03 | A_24_P110242 | 0.964414679 | 4.23E-07 |
| uc021thc.2 | A_24_P110242 | 0.955309905 | 1.30E-06 |
| XR_429426.1 | A_24_P110242 | 0.978194375 | 3.74E-08 |
| XR_429429.1 | A_24_P110242 | 0.986269905 | 3.76E-09 |
| uc002sti.1 | A_24_P110242 | 0.948033068 | 2.73E-06 |
| TCONS_00012168 | KCND3 | 0.908145875 | 4.41E-05 |
| ENST00000510536.1 | KCND3 | 0.924852819 | 1.66E-05 |
| XR_427724.1 | KCND3 | 0.909329299 | 4.14E-05 |
| ENST00000555918.1 | MFAP3L | 0.902762828 | 5.80E-05 |
| ENST00000558120.1 | MFAP3L | 0.924718407 | 1.68E-05 |
| NR_027145.2 | MFAP3L | 0.929415548 | 1.22E-05 |
| ENST00000467896.2 | MFAP3L | 0.906450106 | 4.81E-05 |
| ENST00000420595.1 | MFAP3L | 0.908712344 | 4.28E-05 |
| XR_426860.1 | MFAP3L | 0.914663839 | 3.08E-05 |
| ENST00000602325.1 | MFAP3L | 0.904969417 | 5.19E-05 |
| ENST00000457661.1 | MFAP3L | 0.917803138 | 2.57E-05 |
| NR_037616.1 | MFAP3L | 0.949228381 | 2.44E-06 |
| ENST00000430620.1 | MFAP3L | 0.950625759 | 2.13E-06 |
| ENST00000424116.2 | MFAP3L | 0.915656494 | 2.91E-05 |
| uc022caj.1 | MFAP3L | 0.931261021 | 1.08E-05 |
| TCONS_00022516 | MFAP3L | 0.937402497 | 6.81E-06 |
| ENST00000458479.1 | lnc-C1orf31-1 | 0.908224716 | 4.39E-05 |
| XR_429354.1 | lnc-C1orf31-1 | 0.912979114 | 3.39E-05 |
| TCONS_00026998 | lnc-C1orf31-1 | 0.903685575 | 5.54E-05 |
| NR_024586.1 | lnc-C1orf31-1 | 0.906630185 | 4.77E-05 |
| TCONS_00026223 | lnc-C1orf31-1 | 0.909711339 | 4.05E-05 |
| ENST00000432967.1 | lnc-C1orf31-1 | 0.911030213 | 3.78E-05 |
| ENST00000425104.1 | lnc-C1orf31-1 | 0.966415895 | 3.18E-07 |
| ENST00000440004.1 | lnc-C1orf31-1 | 0.922233245 | 1.96E-05 |
| ENST00000568248.1 | lnc-C1orf31-1 | 0.906688594 | 4.76E-05 |
| ENST00000432411.1 | lnc-C1orf31-1 | 0.914828404 | 3.06E-05 |
| ENST00000565547.1 | lnc-C1orf31-1 | -0.913533254 | 3.29E-05 |
| ENST00000556030.1 | RAB30 | -0.922181067 | 1.97E-05 |
| ENST00000514571.1 | RAB30 | 0.937383099 | 6.82E-06 |
| TCONS_00013375 | RAB30 | 0.912499428 | 3.48E-05 |
| ENST00000564485.1 | PDE3A | 0.910978171 | 3.79E-05 |
| ENST00000573861.1 | OR2L5 | -0.905464376 | 5.06E-05 |
| TCONS_00013375 | RASGRP3 | 0.9093398 | 4.14E-05 |
| ENST00000578583.1 | HDAC9 | -0.903938974 | 5.47E-05 |
| ENST00000609413.1 | HDAC9 | -0.917628378 | 2.60E-05 |
| RNA147577\|p0681_imsncRNA442 | HDAC9 | -0.939587381 | 5.72E-06 |
| ENST00000424245.2 | HDAC9 | -0.901808509 | 6.08E-05 |
| XR_428102.1 | HDAC9 | 0.905774112 | 4.98E-05 |
| RNA146910\|p0014_imsncRNA45 | HDAC9 | -0.90611059 | 4.90E-05 |
| ENST00000555918.1 | PGRMC1 | 0.952121972 | 1.83E-06 |
| ENST00000558120.1 | PGRMC1 | 0.977059355 | 4.82E-08 |
| XR_241556.1 | PGRMC1 | 0.926707299 | 1.47E-05 |
| NR_027145.2 | PGRMC1 | 0.95952948 | 7.99E-07 |
| ENST00000447323.1 | PGRMC1 | 0.906307078 | 4.85E-05 |
| ENST00000561134.1 | PGRMC1 | 0.934462347 | 8.52E-06 |
| TCONS_00018467 | PGRMC1 | 0.915140678 | 3.00E-05 |
| ENST00000442008.2 | PGRMC1 | 0.935390057 | 7.95E-06 |
| ENST00000553317.1 | PGRMC1 | 0.92653018 | 1.49E-05 |
| ENST00000602325.1 | PGRMC1 | 0.928508558 | 1.30E-05 |
| LIT2094 | PGRMC1 | 0.903284187 | 5.65E-05 |
| NR_037928.1 | PGRMC1 | 0.963749984 | 4.64E-07 |
| ENST00000424116.2 | PGRMC1 | 0.953057693 | 1.66E-06 |
| ENST00000607175.1 | PGRMC1 | 0.932657005 | 9.73E-06 |
| uc022caj.1 | PGRMC1 | 0.90831201 | 4.37E-05 |
| TCONS_00022516 | PGRMC1 | 0.928809891 | 1.28E-05 |
| ENST00000555918.1 | SPARC | 0.902489241 | 5.88E-05 |
| ENST00000558120.1 | SPARC | 0.964659854 | 4.09E-07 |
| XR_241556.1 | SPARC | 0.907332786 | 4.60E-05 |
| NR_027145.2 | SPARC | 0.927286426 | 1.42E-05 |
| ENST00000561134.1 | SPARC | 0.92588594 | 1.55E-05 |
| XR_429366.1 | SPARC | 0.900491282 | 6.49E-05 |
| ENST00000442008.2 | SPARC | 0.926016024 | 1.54E-05 |
| ENST00000553317.1 | SPARC | 0.902379829 | 5.91E-05 |
| ENST00000602325.1 | SPARC | 0.907578927 | 4.54E-05 |
| NR_037928.1 | SPARC | 0.942737468 | 4.40E-06 |
| ENST00000424116.2 | SPARC | 0.976685639 | 5.22E-08 |
| ENST00000607175.1 | SPARC | 0.942014974 | 4.68E-06 |
| TCONS_00022516 | SPARC | 0.942257863 | 4.59E-06 |
| HIT000078556_03 | LOC101930405 | 0.935450961 | 7.91E-06 |
| uc021thc.2 | LOC101930405 | 0.905050947 | 5.17E-05 |
| ENST00000430694.1 | LOC101930405 | 0.90332672 | 5.64E-05 |
| XR_429426.1 | LOC101930405 | 0.941175995 | 5.02E-06 |
| NR_026800.1 | LOC101930405 | 0.942535328 | 4.48E-06 |
| XR_429429.1 | LOC101930405 | 0.917048717 | 2.69E-05 |
| NR_022010.1 | PIGK | 0.934727993 | 8.36E-06 |
| ENST00000558120.1 | GP1BB | 0.926023116 | 1.54E-05 |
| XR_241556.1 | GP1BB | 0.919140415 | 2.37E-05 |
| NR_027145.2 | GP1BB | 0.917584759 | 2.60E-05 |
| ENST00000420595.1 | GP1BB | 0.915601603 | 2.92E-05 |
| ENST00000442008.2 | GP1BB | 0.924260428 | 1.73E-05 |
| ENST00000602325.1 | GP1BB | 0.918173314 | 2.52E-05 |
| LIT2094 | GP1BB | 0.936292467 | 7.42E-06 |
| NR_037928.1 | GP1BB | 0.960853515 | 6.78E-07 |
| uc004ehp.2 | GP1BB | 0.9003112 | 6.55E-05 |
| ENST00000424116.2 | GP1BB | 0.96041827 | 7.16E-07 |
| ENST00000607175.1 | GP1BB | 0.922875757 | 1.89E-05 |
| uc022caj.1 | GP1BB | 0.904943959 | 5.20E-05 |
| ENST00000605056.1 | ZNF90 | -0.90164507 | 6.13E-05 |
| HIT000078556_03 | A_21_P0011347 | 0.939221138 | 5.90E-06 |
| uc021thc.2 | A_21_P0011347 | 0.919369904 | 2.34E-05 |
| XR_429426.1 | A_21_P0011347 | 0.929791325 | 1.19E-05 |
| XR_429429.1 | A_21_P0011347 | 0.937294813 | 6.87E-06 |
| ENST00000608442.1 | A_33_P3277096 | 0.933038561 | 9.47E-06 |
| ENST00000602325.1 | A_33_P3277096 | 0.915818677 | 2.89E-05 |
| ENST00000424116.2 | A_33_P3277096 | 0.902603615 | 5.85E-05 |
| ENST00000442008.2 | VSIG2 | 0.91200384 | 3.58E-05 |
| ENST00000424116.2 | VSIG2 | 0.902055626 | 6.01E-05 |
| ENST00000608505.1 | PRKG1 | 0.903398047 | 5.62E-05 |
| ENST00000608442.1 | PRKG1 | 0.903755845 | 5.52E-05 |
| ENST00000445429.1 | PRKG1 | 0.937751877 | 6.63E-06 |
| ENST00000457661.1 | PRKG1 | 0.916130159 | 2.84E-05 |
| ENST00000402410.2 | BMP6 | 0.918875487 | 2.41E-05 |
| ENST00000445429.1 | BMP6 | 0.927884933 | 1.36E-05 |
| ENST00000467896.2 | BMP6 | 0.913016443 | 3.38E-05 |
| ENST00000457661.1 | BMP6 | 0.913426798 | 3.31E-05 |
| NR_037616.1 | BMP6 | 0.974391042 | 8.31E-08 |
| ENST00000556936.1 | BMP6 | 0.920838378 | 2.14E-05 |
| TCONS_00026410 | BMP6 | 0.90707258 | 4.66E-05 |
| ENST00000430620.1 | BMP6 | 0.974135557 | 8.73E-08 |
| ENST00000558120.1 | CDC14B | 0.925759812 | 1.57E-05 |
| NR_027145.2 | CDC14B | 0.923966491 | 1.76E-05 |
| ENST00000561134.1 | CDC14B | 0.921726091 | 2.03E-05 |
| ENST00000420595.1 | CDC14B | 0.911501035 | 3.68E-05 |
| ENST00000442008.2 | CDC14B | 0.905925061 | 4.95E-05 |
| ENST00000553317.1 | CDC14B | 0.919660206 | 2.30E-05 |
| ENST00000602325.1 | CDC14B | 0.927277433 | 1.42E-05 |
| ENST00000506718.1 | CDC14B | 0.909013855 | 4.21E-05 |
| ENST00000424116.2 | CDC14B | 0.930917371 | 1.10E-05 |
| ENST00000607175.1 | CDC14B | 0.955580153 | 1.26E-06 |
| TCONS_00022516 | CDC14B | 0.926547588 | 1.49E-05 |
| XR_429366.1 | ABCC3 | 0.960789605 | 6.83E-07 |
| XR_426860.1 | ABCC3 | 0.904200531 | 5.40E-05 |
| TCONS_00026411 | GNGT1 | 0.908890256 | 4.24E-05 |
| ENST00000445429.1 | GNGT1 | 0.935573083 | 7.84E-06 |
| ENST00000457661.1 | GNGT1 | 0.936481101 | 7.32E-06 |
| ENST00000584509.1 | GNGT1 | 0.92530561 | 1.61E-05 |
| TCONS_00026410 | GNGT1 | 0.904066081 | 5.44E-05 |
| ENST00000430620.1 | GNGT1 | 0.968378532 | 2.36E-07 |
| uc021thc.2 | A_33_P3395675 | 0.928392034 | 1.31E-05 |
| TCONS_00026436 | A_33_P3395675 | 0.901864771 | 6.07E-05 |
| XR_429426.1 | A_33_P3395675 | 0.944600117 | 3.74E-06 |
| XR_429429.1 | A_33_P3395675 | 0.979063672 | 3.06E-08 |
| uc002sti.1 | A_33_P3395675 | 0.983889631 | 8.32E-09 |
| NR_027145.2 | SH3BGRL2 | 0.907498064 | 4.56E-05 |
| ENST00000561134.1 | SH3BGRL2 | 0.919307357 | 2.35E-05 |
| ENST00000420595.1 | SH3BGRL2 | 0.93694733 | 7.06E-06 |
| XR_426860.1 | SH3BGRL2 | 0.931899114 | 1.03E-05 |
| ENST00000602325.1 | SH3BGRL2 | 0.947009915 | 3.01E-06 |
| ENST00000451706.2 | SH3BGRL2 | 0.901463225 | 6.19E-05 |
| TCONS_00010294 | SH3BGRL2 | 0.922736567 | 1.90E-05 |
| ENST00000424116.2 | SH3BGRL2 | 0.919080947 | 2.38E-05 |
| ENST00000607175.1 | SH3BGRL2 | 0.92030087 | 2.21E-05 |
| ENST00000441316.1 | SH3BGRL2 | 0.919886676 | 2.27E-05 |
| TCONS_00022516 | SH3BGRL2 | 0.915350736 | 2.97E-05 |
| uc009wka.2 | FAM122C | 0.914812049 | 3.06E-05 |
| NR_024586.1 | FAM122C | 0.928299436 | 1.32E-05 |
| ENST00000555918.1 | TREML1 | 0.927057197 | 1.44E-05 |
| ENST00000558120.1 | TREML1 | 0.962244482 | 5.67E-07 |
| XR_241556.1 | TREML1 | 0.921778108 | 2.02E-05 |
| NR_027145.2 | TREML1 | 0.942095364 | 4.65E-06 |
| ENST00000561134.1 | TREML1 | 0.937324775 | 6.85E-06 |
| TCONS_00018467 | TREML1 | 0.942857417 | 4.36E-06 |
| XR_429366.1 | TREML1 | 0.908151597 | 4.40E-05 |
| ENST00000442008.2 | TREML1 | 0.909695295 | 4.06E-05 |
| NR_037928.1 | TREML1 | 0.936561602 | 7.27E-06 |
| ENST00000424116.2 | TREML1 | 0.93603132 | 7.57E-06 |
| ENST00000607175.1 | TREML1 | 0.909804303 | 4.03E-05 |
| uc022caj.1 | TREML1 | 0.900481787 | 6.49E-05 |
| TCONS_00022516 | TREML1 | 0.928821602 | 1.28E-05 |
| TCONS_00013375 | lnc-IL6-3 | 0.957192182 | 1.05E-06 |
| ENST00000558120.1 | TUBB1 | 0.950244451 | 2.21E-06 |
| XR_241556.1 | TUBB1 | 0.916882839 | 2.71E-05 |
| LIT2061 | TUBB1 | 0.928570286 | 1.30E-05 |
| NR_027145.2 | TUBB1 | 0.93207011 | 1.02E-05 |
| ENST00000420595.1 | TUBB1 | 0.929667345 | 1.20E-05 |
| ENST00000442008.2 | TUBB1 | 0.904395555 | 5.35E-05 |
| XR_426860.1 | TUBB1 | 0.929053535 | 1.26E-05 |
| ENST00000553317.1 | TUBB1 | 0.912261861 | 3.53E-05 |
| ENST00000602325.1 | TUBB1 | 0.948427612 | 2.63E-06 |
| LIT2094 | TUBB1 | 0.914101678 | 3.18E-05 |
| NR_037928.1 | TUBB1 | 0.920061972 | 2.25E-05 |
| ENST00000506718.1 | TUBB1 | 0.924050894 | 1.75E-05 |
| ENST00000424116.2 | TUBB1 | 0.958631827 | 8.90E-07 |
| ENST00000607175.1 | TUBB1 | 0.947400061 | 2.90E-06 |
| TCONS_00022516 | TUBB1 | 0.964981107 | 3.91E-07 |
| ENST00000439622.1 | HDDC2 | 0.913878369 | 3.22E-05 |
| XR_429354.1 | HDDC2 | 0.903715434 | 5.53E-05 |
| ENST00000602310.1 | HDDC2 | 0.90218186 | 5.97E-05 |
| ENST00000584683.1 | HDDC2 | 0.914079248 | 3.19E-05 |
| TCONS_00026998 | HDDC2 | 0.94352912 | 4.11E-06 |
| TCONS_00013891 | HDDC2 | 0.903144737 | 5.69E-05 |
| NR_110099.1 | HDDC2 | 0.913444087 | 3.30E-05 |
| ENST00000426704.1 | HDDC2 | 0.921700935 | 2.03E-05 |
| NR_027145.2 | MITF | 0.915169553 | 3.00E-05 |
| ENST00000447323.1 | MITF | 0.958062298 | 9.52E-07 |
| ENST00000467896.2 | MITF | 0.917607802 | 2.60E-05 |
| ENST00000567327.1 | MITF | 0.929211061 | 1.24E-05 |
| ENST00000457661.1 | MITF | 0.927398133 | 1.41E-05 |
| XR_427746.1 | MITF | 0.904414983 | 5.34E-05 |
| TCONS_00026410 | MITF | 0.918900625 | 2.41E-05 |
| ENST00000430620.1 | MITF | 0.920056929 | 2.25E-05 |
| XR_427724.1 | MITF | 0.902805134 | 5.79E-05 |
| XR_245040.2 | NAPSA | -0.907137668 | 4.65E-05 |
| TCONS_00026539 | BMP7 | 0.911651012 | 3.65E-05 |
| TCONS_00026538 | BMP7 | 0.933012135 | 9.49E-06 |
| ENST00000418746.1 | BMP7 | 0.900473447 | 6.49E-05 |
| TCONS_00018467 | NFIB | 0.924297276 | 1.72E-05 |
| ENST00000609423.1 | NFIB | -0.912711045 | 3.44E-05 |
| TCONS_00018641 | NFIB | 0.939438585 | 5.79E-06 |
| uc004ehp.2 | NFIB | 0.911547882 | 3.67E-05 |
| uc022caj.1 | NFIB | 0.901419341 | 6.20E-05 |
| ENST00000555918.1 | ZNF438 | 0.923241832 | 1.84E-05 |
| NR_027145.2 | ZNF438 | 0.929070614 | 1.25E-05 |
| TCONS_00018467 | ZNF438 | 0.988911623 | 1.30E-09 |
| ENST00000609423.1 | ZNF438 | -0.909902556 | 4.01E-05 |
| ENST00000592441.1 | ZNF438 | 0.907226692 | 4.62E-05 |
| ENST00000558120.1 | C6orf25 | 0.923263451 | 1.84E-05 |
| ENST00000561134.1 | C6orf25 | 0.938949297 | 6.03E-06 |
| ENST00000442008.2 | C6orf25 | 0.927474599 | 1.40E-05 |
| NR_037928.1 | C6orf25 | 0.93137891 | 1.07E-05 |
| ENST00000424116.2 | C6orf25 | 0.933409623 | 9.21E-06 |
| TCONS_00022516 | C6orf25 | 0.91504388 | 3.02E-05 |
| ENST00000558120.1 | CMTM5 | 0.916692908 | 2.74E-05 |
| XR_241556.1 | CMTM5 | 0.90639334 | 4.83E-05 |
| NR_027145.2 | CMTM5 | 0.906475739 | 4.81E-05 |
| ENST00000561134.1 | CMTM5 | 0.906487143 | 4.80E-05 |
| ENST00000420595.1 | CMTM5 | 0.91878067 | 2.43E-05 |
| ENST00000442008.2 | CMTM5 | 0.93945273 | 5.79E-06 |
| ENST00000602325.1 | CMTM5 | 0.912625992 | 3.46E-05 |
| LIT2094 | CMTM5 | 0.944654602 | 3.73E-06 |
| NR_037928.1 | CMTM5 | 0.939835037 | 5.61E-06 |
| ENST00000424116.2 | CMTM5 | 0.952971846 | 1.67E-06 |
| ENST00000607175.1 | CMTM5 | 0.934010455 | 8.82E-06 |
| HIT000078556_03 | A_33_P3240182 | 0.950255482 | 2.21E-06 |
| uc021thc.2 | A_33_P3240182 | 0.941238964 | 5.00E-06 |
| XR_429426.1 | A_33_P3240182 | 0.914992535 | 3.03E-05 |
| XR_429429.1 | A_33_P3240182 | 0.942525253 | 4.48E-06 |
| ENST00000608505.1 | ATP5E | 0.918401215 | 2.48E-05 |
| ENST00000558120.1 | ATP5E | 0.946778278 | 3.07E-06 |
| XR_241556.1 | ATP5E | 0.905214487 | 5.13E-05 |
| LIT2061 | ATP5E | 0.932279215 | 1.00E-05 |
| NR_027145.2 | ATP5E | 0.912308251 | 3.52E-05 |
| ENST00000442008.2 | ATP5E | 0.902455028 | 5.89E-05 |
| XR_426860.1 | ATP5E | 0.912514547 | 3.48E-05 |
| ENST00000553317.1 | ATP5E | 0.908776216 | 4.26E-05 |
| ENST00000602325.1 | ATP5E | 0.942662081 | 4.43E-06 |
| LIT2094 | ATP5E | 0.901320835 | 6.23E-05 |
| NR_037928.1 | ATP5E | 0.923118139 | 1.86E-05 |
| ENST00000457661.1 | ATP5E | 0.913477066 | 3.30E-05 |
| ENST00000424116.2 | ATP5E | 0.974589994 | 7.99E-08 |
| ENST00000607175.1 | ATP5E | 0.950401765 | 2.17E-06 |
| ENST00000556996.1 | ATP5E | 0.904786854 | 5.24E-05 |
| TCONS_00022516 | ATP5E | 0.958800318 | 8.72E-07 |
| ENST00000555918.1 | SLC35D2 | 0.90413079 | 5.42E-05 |
| NR_027145.2 | SLC35D2 | 0.906560015 | 4.79E-05 |
| RNA95734\|RNS_816_119 | SLC35D2 | 0.902863197 | 5.77E-05 |
| HIT000078556_03 | A_33_P3331178 | 0.970941808 | 1.55E-07 |
| uc021thc.2 | A_33_P3331178 | 0.957817273 | 9.80E-07 |
| XR_429426.1 | A_33_P3331178 | 0.96041661 | 7.16E-07 |
| XR_429429.1 | A_33_P3331178 | 0.96162058 | 6.15E-07 |
| uc002sti.1 | A_33_P3331178 | 0.903507588 | 5.59E-05 |
| uc021vkt.1 | A_33_P3331178 | 0.90765691 | 4.52E-05 |
| ENST00000606593.1 | VAV2 | -0.915935038 | 2.87E-05 |
| ENST00000458479.1 | ZNF780A | 0.926677583 | 1.47E-05 |
| ENST00000523456.1 | ZNF780A | 0.909332897 | 4.14E-05 |
| TCONS_00013891 | ZNF780A | 0.935125327 | 8.11E-06 |
| ENST00000535911.1 | ZNF780A | 0.907836929 | 4.48E-05 |
| ENST00000443574.1 | ZNF780A | -0.908733515 | 4.27E-05 |
| ENST00000416310.1 | ZNF780A | 0.924637663 | 1.69E-05 |
| LIT2061 | ENDOD1 | 0.907460311 | 4.57E-05 |
| XR_429366.1 | ENDOD1 | 0.929294863 | 1.23E-05 |
| XR_426860.1 | ENDOD1 | 0.907578777 | 4.54E-05 |
| ENST00000506718.1 | ENDOD1 | 0.916984101 | 2.70E-05 |
| TCONS_00010294 | ENDOD1 | 0.94221347 | 4.60E-06 |
| ENST00000441316.1 | ENDOD1 | 0.919986278 | 2.26E-05 |
| TCONS_00022516 | ENDOD1 | 0.927467753 | 1.40E-05 |
| ENST00000555918.1 | XK | 0.90770766 | 4.51E-05 |
| ENST00000558120.1 | XK | 0.925823829 | 1.56E-05 |
| XR_241556.1 | XK | 0.956503393 | 1.14E-06 |
| NR_027145.2 | XK | 0.943277309 | 4.20E-06 |
| ENST00000561134.1 | XK | 0.904677233 | 5.27E-05 |
| ENST00000420595.1 | XK | 0.954282762 | 1.46E-06 |
| TCONS_00018335 | XK | 0.913793149 | 3.24E-05 |
| XR_426860.1 | XK | 0.906714692 | 4.75E-05 |
| ENST00000602325.1 | XK | 0.961793549 | 6.01E-07 |
| ENST00000451706.2 | XK | 0.923518852 | 1.81E-05 |
| LIT2094 | XK | 0.926489023 | 1.49E-05 |
| NR_037928.1 | XK | 0.933226036 | 9.34E-06 |
| ENST00000457661.1 | XK | 0.910697586 | 3.84E-05 |
| ENST00000424116.2 | XK | 0.93860146 | 6.20E-06 |
| ENST00000607175.1 | XK | 0.930965378 | 1.10E-05 |
| TCONS_00022516 | XK | 0.904710202 | 5.26E-05 |
| uc021thc.2 | A_24_P627503 | 0.914309217 | 3.15E-05 |
| NR_027145.2 | LOC102724479 | 0.934089658 | 8.76E-06 |
| ENST00000447323.1 | LOC102724479 | 0.922732096 | 1.90E-05 |
| ENST00000467896.2 | LOC102724479 | 0.996620098 | 3.45E-12 |
| RNA95734\|RNS_816_119 | LOC102724479 | 0.902460831 | 5.89E-05 |
| ENST00000513153.1 | LOC102724479 | 0.950366402 | 2.18E-06 |
| NR_037616.1 | LOC102724479 | 0.929297334 | 1.23E-05 |
| uc004ehp.2 | LOC102724479 | 0.906181722 | 4.88E-05 |
| TCONS_00026410 | LOC102724479 | 0.919718552 | 2.29E-05 |
| ENST00000430620.1 | LOC102724479 | 0.922847149 | 1.89E-05 |
| uc022caj.1 | LOC102724479 | 0.907763195 | 4.50E-05 |
| ENST00000585784.1 | TPTE | 0.959694588 | 7.83E-07 |
| XR_427192.1 | ETFA | 0.953522613 | 1.58E-06 |
| ENST00000577848.1 | ETFA | 0.919152554 | 2.37E-05 |
| ENST00000416080.1 | ETFA | 0.932687721 | 9.71E-06 |
| TCONS_00012168 | ETFA | 0.916211847 | 2.82E-05 |
| NR_027145.2 | ETFA | 0.92292485 | 1.88E-05 |
| ENST00000510536.1 | ETFA | 0.967385407 | 2.75E-07 |
| ENST00000467896.2 | ETFA | 0.920824079 | 2.14E-05 |
| ENST00000451706.2 | ETFA | 0.925360091 | 1.61E-05 |
| ENST00000458314.1 | ETFA | 0.952985265 | 1.67E-06 |
| ENST00000513153.1 | ETFA | 0.919104679 | 2.38E-05 |
| ENST00000450238.1 | ETFA | 0.904800861 | 5.24E-05 |
| ENST00000430620.1 | ETFA | 0.915448509 | 2.95E-05 |
| XR_427724.1 | ETFA | 0.959314178 | 8.20E-07 |
| uc022caj.1 | ETFA | 0.901864198 | 6.07E-05 |
| uc004ehp.2 | FKBP1B | 0.902088894 | 6.00E-05 |
| uc022caj.1 | FKBP1B | 0.911260167 | 3.73E-05 |
| TCONS_00018467 | ST7 | 0.905722616 | 5.00E-05 |
| TCONS_00018641 | ST7 | 0.93027278 | 1.15E-05 |
| ENST00000555918.1 | TRIM58 | 0.904596635 | 5.29E-05 |
| ENST00000558120.1 | TRIM58 | 0.932342443 | 9.96E-06 |
| XR_241556.1 | TRIM58 | 0.932706929 | 9.70E-06 |
| NR_027145.2 | TRIM58 | 0.940088087 | 5.49E-06 |
| ENST00000420595.1 | TRIM58 | 0.948124582 | 2.71E-06 |
| TCONS_00018335 | TRIM58 | 0.945383995 | 3.49E-06 |
| ENST00000442008.2 | TRIM58 | 0.911401025 | 3.70E-05 |
| ENST00000602325.1 | TRIM58 | 0.944660406 | 3.72E-06 |
| LIT2094 | TRIM58 | 0.947653091 | 2.83E-06 |
| NR_037928.1 | TRIM58 | 0.933232398 | 9.34E-06 |
| uc004ehp.2 | TRIM58 | 0.916774001 | 2.73E-05 |
| ENST00000424116.2 | TRIM58 | 0.910399716 | 3.91E-05 |
| uc022caj.1 | TRIM58 | 0.92427885 | 1.72E-05 |
| TCONS_00022516 | TRIM58 | 0.937667475 | 6.67E-06 |
| RNA147249\|p0353_imsncRNA771 | lnc-IL6-3 | 0.919390986 | 2.34E-05 |
| RNA147248\|p0352_imsncRNA771 | lnc-IL6-3 | 0.928733479 | 1.28E-05 |
| ENST00000530194.1 | lnc-IL6-3 | 0.9531325 | 1.65E-06 |
| ENST00000439823.1 | lnc-IL6-3 | 0.98963775 | 9.25E-10 |
| TCONS_00013375 | lnc-IL6-3 | 0.960591399 | 7.01E-07 |
| XR_426970.1 | ZFHX3 | 0.91402135 | 3.20E-05 |
| ENST00000558120.1 | C6orf25 | 0.927254456 | 1.42E-05 |
| ENST00000561134.1 | C6orf25 | 0.922245885 | 1.96E-05 |
| ENST00000442008.2 | C6orf25 | 0.903928361 | 5.48E-05 |
| NR_037928.1 | C6orf25 | 0.933702701 | 9.02E-06 |
| ENST00000424116.2 | C6orf25 | 0.944992788 | 3.62E-06 |
| TCONS_00022516 | C6orf25 | 0.902948634 | 5.75E-05 |
| uc009wka.2 | ZNF239 | 0.910999528 | 3.78E-05 |
| NR_024586.1 | ZNF239 | 0.901696519 | 6.12E-05 |
| ENST00000535911.1 | ZNF239 | 0.917043255 | 2.69E-05 |
| TCONS_00000305 | ZNF239 | 0.953341814 | 1.61E-06 |
| XR_158905.1 | ZNF239 | 0.90366474 | 5.55E-05 |
| ENST00000607314.1 | NRG1 | 0.925511079 | 1.59E-05 |
| NR_037928.1 | ITGB5 | 0.910358024 | 3.92E-05 |
| TCONS_00024392 | ITGB5 | -0.900422806 | 6.51E-05 |
| ENST00000448017.1 | lnc-MBL2-3 | 0.98613458 | 3.94E-09 |
| ENST00000443523.1 | lnc-MBL2-3 | 0.989591777 | 9.45E-10 |
| NR_026710.1 | lnc-MBL2-3 | 0.911177785 | 3.75E-05 |
| HIT000078556_03 | A_23_P390209 | 0.958017751 | 9.57E-07 |
| uc021thc.2 | A_23_P390209 | 0.926370191 | 1.50E-05 |
| XR_429426.1 | A_23_P390209 | 0.952823514 | 1.70E-06 |
| XR_429429.1 | A_23_P390209 | 0.949012675 | 2.49E-06 |
| uc002sti.1 | A_23_P390209 | 0.919584503 | 2.31E-05 |
| ENST00000608241.1 | CCDC83 | -0.900945712 | 6.35E-05 |
| uc004ehp.2 | CCDC83 | -0.950027577 | 2.26E-06 |
| uc022caj.1 | CCDC83 | -0.950597252 | 2.13E-06 |
| ENST00000558120.1 | A_21_P0013153 | 0.920367935 | 2.20E-05 |
| ENST00000560924.1 | A_21_P0013153 | 0.929850838 | 1.19E-05 |
| RNA147574\|p0678_imsncRNA439 | CABP5 | -0.910677218 | 3.85E-05 |
| ENST00000513153.1 | CABP5 | 0.912081644 | 3.56E-05 |
| ENST00000608505.1 | PTGS1 | 0.903592646 | 5.57E-05 |
| XR_241556.1 | PTGS1 | 0.955251032 | 1.31E-06 |
| ENST00000561134.1 | PTGS1 | 0.917176478 | 2.67E-05 |
| ENST00000420595.1 | PTGS1 | 0.922820906 | 1.89E-05 |
| ENST00000602325.1 | PTGS1 | 0.98425266 | 7.43E-09 |
| LIT2094 | PTGS1 | 0.928285741 | 1.32E-05 |
| NR_037928.1 | PTGS1 | 0.929623607 | 1.21E-05 |
| ENST00000457661.1 | PTGS1 | 0.907584124 | 4.54E-05 |
| ENST00000424116.2 | PTGS1 | 0.972774523 | 1.13E-07 |
| ENST00000607175.1 | PTGS1 | 0.918913768 | 2.41E-05 |
| TCONS_00022516 | PTGS1 | 0.922880071 | 1.89E-05 |
| ENST00000402410.2 | PARD3 | 0.925563563 | 1.59E-05 |
| TCONS_00026539 | PARD3 | 0.930837768 | 1.11E-05 |
| TCONS_00026538 | PARD3 | 0.920596594 | 2.17E-05 |
| ENST00000458479.1 | CCDC37 | 0.924041603 | 1.75E-05 |
| ENST00000555918.1 | ANKRD9 | 0.90226082 | 5.95E-05 |
| ENST00000558120.1 | ANKRD9 | 0.948252361 | 2.68E-06 |
| LIT2061 | ANKRD9 | 0.941467166 | 4.90E-06 |
| NR_027145.2 | ANKRD9 | 0.940016995 | 5.53E-06 |
| ENST00000608442.1 | ANKRD9 | 0.900291988 | 6.55E-05 |
| ENST00000554253.1 | ANKRD9 | 0.923811623 | 1.78E-05 |
| ENST00000420595.1 | ANKRD9 | 0.924136617 | 1.74E-05 |
| ENST00000442008.2 | ANKRD9 | 0.947070417 | 2.99E-06 |
| ENST00000553317.1 | ANKRD9 | 0.933165372 | 9.38E-06 |
| ENST00000602325.1 | ANKRD9 | 0.901848053 | 6.07E-05 |
| LIT2094 | ANKRD9 | 0.924295703 | 1.72E-05 |
| NR_037928.1 | ANKRD9 | 0.926989457 | 1.44E-05 |
| ENST00000506718.1 | ANKRD9 | 0.905681646 | 5.01E-05 |
| ENST00000424116.2 | ANKRD9 | 0.959028638 | 8.49E-07 |
| ENST00000607175.1 | ANKRD9 | 0.968471157 | 2.33E-07 |
| uc022caj.1 | ANKRD9 | 0.912095857 | 3.56E-05 |
| TCONS_00022516 | ANKRD9 | 0.920579783 | 2.18E-05 |
| TCONS_00007196 | MSN | 0.903655353 | 5.55E-05 |
| NR_027145.2 | MSN | 0.921801458 | 2.02E-05 |
| ENST00000447323.1 | MSN | 0.98852417 | 1.54E-09 |
| ENST00000467896.2 | MSN | 0.908543982 | 4.31E-05 |
| XR_427724.1 | MSN | 0.923030695 | 1.87E-05 |
| HIT000078556_03 | A_33_P3331193 | 0.944110699 | 3.91E-06 |
| uc021thc.2 | A_33_P3331193 | 0.932139481 | 1.01E-05 |
| XR_429426.1 | A_33_P3331193 | 0.988124407 | 1.82E-09 |
| NR_026800.1 | A_33_P3331193 | 0.922981916 | 1.87E-05 |
| XR_429429.1 | A_33_P3331193 | 0.955285043 | 1.31E-06 |
| uc002sti.1 | A_33_P3331193 | 0.902068952 | 6.01E-05 |
| uc021vkt.1 | A_33_P3331193 | 0.923676422 | 1.79E-05 |
| TCONS_00018467 | ZNF385D | 0.939571152 | 5.73E-06 |
| TCONS_00018641 | ZNF385D | 0.936835901 | 7.12E-06 |
| XR_429354.1 | TSGA10 | 0.911163413 | 3.75E-05 |
| TCONS_00013891 | TSGA10 | 0.926337474 | 1.51E-05 |
| ENST00000440004.1 | TSGA10 | 0.903641554 | 5.55E-05 |
| ENST00000600477.1 | MITF | -0.908922014 | 4.23E-05 |
| ENST00000457661.1 | MITF | 0.918471594 | 2.47E-05 |
| RNA95734\|RNS_816_119 | HIST1H2AG | 0.900868694 | 6.37E-05 |
| ENST00000450238.1 | HIST1H2AG | 0.907419095 | 4.58E-05 |
| TCONS_00010294 | HIST1H2AG | 0.912397003 | 3.50E-05 |
| ENST00000605136.1 | IFI27L2 | -0.93297317 | 9.51E-06 |
| NR_027034.1 | IFI27L2 | -0.957549502 | 1.01E-06 |
| RNA146910\|p0014_imsncRNA45 | IFI27L2 | -0.917121958 | 2.68E-05 |
| XR_428739.1 | CCDC13 | -0.925622904 | 1.58E-05 |
| ENST00000412353.1 | CCDC13 | -0.909949167 | 4.00E-05 |
| XR_244697.1 | CCDC13 | 0.907350568 | 4.59E-05 |
| ENST00000592381.1 | MDFIC | 0.914431336 | 3.13E-05 |
| TCONS_00026998 | MDFIC | 0.916039028 | 2.85E-05 |
| XR_246176.1 | MDFIC | 0.913059805 | 3.38E-05 |
| ENST00000527067.1 | MDFIC | 0.907752426 | 4.50E-05 |
| ENST00000413818.2 | MDFIC | 0.910806007 | 3.82E-05 |
| ENST00000558120.1 | SELP | 0.938034182 | 6.48E-06 |
| XR_241556.1 | SELP | 0.935249112 | 8.04E-06 |
| NR_027145.2 | SELP | 0.9121715 | 3.55E-05 |
| ENST00000561134.1 | SELP | 0.908804153 | 4.26E-05 |
| TCONS_00018467 | SELP | 0.901301834 | 6.24E-05 |
| LIT2094 | SELP | 0.904888674 | 5.22E-05 |
| NR_037928.1 | SELP | 0.943839771 | 4.00E-06 |
| ENST00000424116.2 | SELP | 0.96196231 | 5.88E-07 |
| ENST00000607175.1 | SELP | 0.930283666 | 1.15E-05 |
| TCONS_00022516 | SELP | 0.908648209 | 4.29E-05 |
| ENST00000606593.1 | UNC93B1 | -0.90939557 | 4.12E-05 |
| ENST00000556030.1 | TLE1 | -0.91217295 | 3.55E-05 |
| ENST00000561460.1 | TLE1 | 0.901855413 | 6.07E-05 |
| ENST00000514571.1 | TLE1 | 0.901891625 | 6.06E-05 |
| TCONS_00012168 | PRTFDC1 | 0.934376167 | 8.58E-06 |
| ENST00000558120.1 | PRTFDC1 | 0.907219611 | 4.63E-05 |
| LIT2061 | PRTFDC1 | 0.910052511 | 3.98E-05 |
| NR_027145.2 | PRTFDC1 | 0.955465249 | 1.28E-06 |
| ENST00000608442.1 | PRTFDC1 | 0.916866324 | 2.72E-05 |
| ENST00000420595.1 | PRTFDC1 | 0.942938472 | 4.33E-06 |
| TCONS_00018335 | PRTFDC1 | 0.90439343 | 5.35E-05 |
| ENST00000553317.1 | PRTFDC1 | 0.900244028 | 6.57E-05 |
| ENST00000602325.1 | PRTFDC1 | 0.91894436 | 2.40E-05 |
| ENST00000424116.2 | PRTFDC1 | 0.912845377 | 3.42E-05 |
| ENST00000607175.1 | PRTFDC1 | 0.963054767 | 5.09E-07 |
| XR_427724.1 | PRTFDC1 | 0.91373372 | 3.25E-05 |
| HIT000078556_03 | A_32_P190951 | 0.942575572 | 4.46E-06 |
| uc021thc.2 | A_32_P190951 | 0.925757969 | 1.57E-05 |
| XR_429426.1 | A_32_P190951 | 0.974864977 | 7.57E-08 |
| NR_026800.1 | A_32_P190951 | 0.94556723 | 3.43E-06 |
| XR_429429.1 | A_32_P190951 | 0.934740696 | 8.35E-06 |
| uc021vkt.1 | A_32_P190951 | 0.917823178 | 2.57E-05 |
| HIT000078556_03 | A_33_P3223964 | 0.960783657 | 6.84E-07 |
| uc021thc.2 | A_33_P3223964 | 0.932239481 | 1.00E-05 |
| XR_429426.1 | A_33_P3223964 | 0.957483828 | 1.02E-06 |
| NR_026800.1 | A_33_P3223964 | 0.955291938 | 1.30E-06 |
| XR_429429.1 | A_33_P3223964 | 0.931436801 | 1.06E-05 |
| XR_108954.2 | CARD9 | 0.922619712 | 1.92E-05 |
| ENST00000444125.1 | CARD9 | 0.900078119 | 6.62E-05 |
| HIT000078556_03 | A_33_P3301075 | 0.968020033 | 2.50E-07 |
| uc021thc.2 | A_33_P3301075 | 0.920917024 | 2.13E-05 |
| XR_429426.1 | A_33_P3301075 | 0.949446346 | 2.39E-06 |
| NR_026800.1 | A_33_P3301075 | 0.904667899 | 5.27E-05 |
| XR_429429.1 | A_33_P3301075 | 0.944041705 | 3.93E-06 |
| XR_241556.1 | RTN2 | 0.928519893 | 1.30E-05 |
| ENST00000561134.1 | RTN2 | 0.913213552 | 3.35E-05 |
| NR_037928.1 | RTN2 | 0.941936254 | 4.71E-06 |
| ENST00000424116.2 | RTN2 | 0.910098173 | 3.97E-05 |
| ENST00000458479.1 | GDAP1L1 | 0.931170135 | 1.08E-05 |
| NR_022010.1 | GDAP1L1 | 0.911777879 | 3.62E-05 |
| NR_026880.1 | GDAP1L1 | 0.921309206 | 2.08E-05 |
| LIT1273 | GDAP1L1 | 0.901113299 | 6.29E-05 |
| ENST00000583262.1 | GDAP1L1 | 0.908683496 | 4.28E-05 |
| XR_428131.1 | GDAP1L1 | 0.922539387 | 1.93E-05 |
| ENST00000432411.1 | GDAP1L1 | 0.939050016 | 5.98E-06 |
| ENST00000559026.1 | NAPSA | 0.931245801 | 1.08E-05 |
| XR_108954.2 | IQSEC2 | 0.900607931 | 6.45E-05 |
| ENST00000444125.1 | IQSEC2 | 0.911618585 | 3.66E-05 |
| XR_428603.1 | IQSEC2 | 0.917654951 | 2.59E-05 |
| XR_108954.2 | MARCO | 0.915768641 | 2.90E-05 |
| ENST00000444125.1 | MARCO | 0.905998277 | 4.93E-05 |
| ENST00000449620.2 | MARCO | 0.933998533 | 8.82E-06 |
| ENST00000527450.1 | MARCO | -0.908591028 | 4.30E-05 |
| HIT000078556_03 | A_24_P101642 | 0.974371838 | 8.34E-08 |
| uc021thc.2 | A_24_P101642 | 0.964248684 | 4.33E-07 |
| XR_429426.1 | A_24_P101642 | 0.961763334 | 6.04E-07 |
| XR_429429.1 | A_24_P101642 | 0.987100345 | 2.75E-09 |
| uc002sti.1 | A_24_P101642 | 0.944460782 | 3.79E-06 |
| ENST00000506665.1 | ANKRD18A | -0.905759865 | 4.99E-05 |
| ENST00000510536.1 | ANKRD18A | -0.935501496 | 7.88E-06 |
| ENST00000562942.1 | ANKRD18A | 0.95750056 | 1.02E-06 |
| NR_047651.1 | ANKRD18A | 0.939311123 | 5.85E-06 |
| ENST00000558120.1 | PF4 | 0.907008241 | 4.68E-05 |
| NR_027145.2 | PF4 | 0.927744706 | 1.37E-05 |
| ENST00000608442.1 | PF4 | 0.921073793 | 2.11E-05 |
| ENST00000420595.1 | PF4 | 0.935186685 | 8.07E-06 |
| TCONS_00018335 | PF4 | 0.931433432 | 1.06E-05 |
| ENST00000442008.2 | PF4 | 0.936267164 | 7.44E-06 |
| ENST00000553317.1 | PF4 | 0.901142089 | 6.29E-05 |
| LIT2094 | PF4 | 0.939518914 | 5.76E-06 |
| NR_037928.1 | PF4 | 0.915875249 | 2.88E-05 |
| uc004ehp.2 | PF4 | 0.934724022 | 8.36E-06 |
| ENST00000424116.2 | PF4 | 0.921498784 | 2.06E-05 |
| ENST00000607175.1 | PF4 | 0.923197246 | 1.85E-05 |
| uc022caj.1 | PF4 | 0.94361833 | 4.08E-06 |
| ENST00000610185.1 | PF4 | 0.914378475 | 3.13E-05 |
| ENST00000602325.1 | BEND2 | 0.908897876 | 4.23E-05 |
| ENST00000506718.1 | BEND2 | 0.92006993 | 2.24E-05 |
| NR_027034.1 | PEBP4 | 0.926523786 | 1.49E-05 |
| XR_108954.2 | PEBP4 | -0.906653128 | 4.76E-05 |
| XR_427746.1 | PEBP4 | -0.908140167 | 4.41E-05 |
| ENST00000525714.1 | PKIG | 0.901396829 | 6.21E-05 |
| RNA147249\|p0353_imsncRNA771 | PKIG | 0.911907665 | 3.60E-05 |
| RNA147248\|p0352_imsncRNA771 | PKIG | 0.903367178 | 5.63E-05 |
| ENST00000606593.1 | PKIG | -0.916674213 | 2.75E-05 |
| TCONS_00006817 | PKIG | -0.902593705 | 5.85E-05 |
| ENST00000430694.1 | LMNTD2 | 0.930374325 | 1.15E-05 |
| ENST00000430620.1 | MEIS1 | 0.912694067 | 3.45E-05 |
| ENST00000548170.1 | SPAG11B | 0.989549898 | 9.64E-10 |
| ENST00000528726.1 | SPAG11B | 0.954580078 | 1.41E-06 |
| ENST00000418255.1 | SPAG11B | 0.93768954 | 6.66E-06 |
| ENST00000565547.1 | SPAG11B | 0.965989647 | 3.38E-07 |
| TCONS_00006023 | SPAG11B | 0.907744571 | 4.50E-05 |
| XR_427192.1 | ENKUR | 0.946106222 | 3.27E-06 |
| ENST00000577848.1 | ENKUR | 0.920767401 | 2.15E-05 |
| ENST00000416080.1 | ENKUR | 0.923650643 | 1.80E-05 |
| ENST00000506665.1 | ENKUR | 0.906883512 | 4.71E-05 |
| ENST00000510536.1 | ENKUR | 0.958177817 | 9.39E-07 |
| ENST00000467896.2 | ENKUR | 0.915465415 | 2.95E-05 |
| ENST00000458314.1 | ENKUR | 0.950640406 | 2.12E-06 |
| ENST00000513153.1 | ENKUR | 0.914124931 | 3.18E-05 |
| RNA147249\|p0353_imsncRNA771 | CD79B | 0.933869399 | 8.91E-06 |
| RNA147248\|p0352_imsncRNA771 | CD79B | 0.94494559 | 3.63E-06 |
| ENST00000530194.1 | CD79B | 0.935940141 | 7.62E-06 |
| ENST00000439823.1 | CD79B | 0.9022541 | 5.95E-05 |
| TCONS_00013375 | CD79B | 0.905677935 | 5.01E-05 |
| TCONS_00017180 | CD79B | 0.929022081 | 1.26E-05 |
| ENST00000454086.2 | ELOVL6 | 0.912740192 | 3.44E-05 |
| TCONS_00024392 | JAM3 | -0.911858932 | 3.61E-05 |
| HIT000078556_03 | A_33_P3268664 | 0.984464621 | 6.94E-09 |
| uc021thc.2 | A_33_P3268664 | 0.917293478 | 2.65E-05 |
| XR_429426.1 | A_33_P3268664 | 0.901944741 | 6.04E-05 |
| XR_429429.1 | A_33_P3268664 | 0.905008263 | 5.18E-05 |
| uc021thc.2 | MZB1 | 0.920881349 | 2.14E-05 |
| TCONS_00026436 | MZB1 | 0.93479463 | 8.32E-06 |
| XR_429426.1 | MZB1 | 0.912766739 | 3.43E-05 |
| XR_429429.1 | MZB1 | 0.9398557 | 5.60E-06 |
| uc002sti.1 | MZB1 | 0.941270325 | 4.98E-06 |
| TCONS_00016405 | HLF | -0.945987254 | 3.31E-06 |
| ENST00000609423.1 | C5orf66-AS1 | 0.909583194 | 4.08E-05 |
| ENST00000443523.1 | MYL2 | 0.90648239 | 4.81E-05 |
| HIT000078556_03 | A_19_P00801412 | 0.926536785 | 1.49E-05 |
| uc021thc.2 | A_19_P00801412 | 0.957788287 | 9.83E-07 |
| XR_429426.1 | A_19_P00801412 | 0.942529657 | 4.48E-06 |
| XR_429429.1 | A_19_P00801412 | 0.929990672 | 1.18E-05 |
| uc021vkt.1 | A_19_P00801412 | 0.910174004 | 3.95E-05 |
| ENST00000452622.1 | PKHD1L1 | 0.903454146 | 5.61E-05 |
| ENST00000558120.1 | PTCRA | 0.917640235 | 2.60E-05 |
| XR_241556.1 | PTCRA | 0.927093789 | 1.43E-05 |
| NR_027145.2 | PTCRA | 0.921512575 | 2.05E-05 |
| TCONS_00018467 | PTCRA | 0.929989455 | 1.18E-05 |
| NR_037928.1 | PTCRA | 0.926750847 | 1.47E-05 |
| ENST00000523456.1 | UTS2 | -0.901105619 | 6.30E-05 |
| TCONS_00026411 | FHL2 | 0.911843146 | 3.61E-05 |
| ENST00000448017.1 | HIST2H2BE | 0.903735481 | 5.53E-05 |
| ENST00000443523.1 | HIST2H2BE | 0.917430467 | 2.63E-05 |
| ENST00000534162.1 | HIST2H2BE | 0.936170157 | 7.49E-06 |
| HIT000078556_03 | A_32_P159192 | 0.919892549 | 2.27E-05 |
| uc021thc.2 | A_32_P159192 | 0.957319753 | 1.04E-06 |
| TCONS_00026436 | A_32_P159192 | 0.910775287 | 3.83E-05 |
| XR_429426.1 | A_32_P159192 | 0.959747626 | 7.78E-07 |
| XR_429429.1 | A_32_P159192 | 0.97980329 | 2.56E-08 |
| uc002sti.1 | A_32_P159192 | 0.972628763 | 1.16E-07 |
| TCONS_00007196 | MITF | 0.956854708 | 1.10E-06 |
| ENST00000447323.1 | MITF | 0.952270481 | 1.80E-06 |
| ENST00000467896.2 | MITF | 0.917164438 | 2.67E-05 |
| ENST00000567327.1 | MITF | 0.923493179 | 1.81E-05 |
| TCONS_00026410 | MITF | 0.918819169 | 2.42E-05 |
| RNA95734\|RNS_816_119 | HIST1H3H | 0.928113994 | 1.34E-05 |
| XR_430056.1 | HIST1H3H | 0.923930793 | 1.76E-05 |
| uc021thc.2 | IGLL1 | 0.924242325 | 1.73E-05 |
| XR_429426.1 | IGLL1 | 0.931272586 | 1.08E-05 |
| XR_429429.1 | IGLL1 | 0.968449057 | 2.34E-07 |
| uc002sti.1 | IGLL1 | 0.980034378 | 2.42E-08 |
| XR_108954.2 | LGALS3 | 0.925918501 | 1.55E-05 |
| ENST00000439622.1 | A_21_P0012114 | 0.900882092 | 6.37E-05 |
| XR_429354.1 | A_21_P0012114 | 0.960707695 | 6.90E-07 |
| TCONS_00026223 | A_21_P0012114 | 0.95193749 | 1.86E-06 |
| ENST00000607950.1 | A_21_P0012114 | -0.902850458 | 5.78E-05 |
| NR_026800.1 | POU2AF1 | 0.919040181 | 2.39E-05 |
| XR_426862.1 | PLXDC1 | 0.900359828 | 6.53E-05 |
| ENST00000534089.1 | PLXDC1 | 0.917047261 | 2.69E-05 |
| uc001ttj.1 | PLXDC1 | 0.919474826 | 2.33E-05 |
| uc021thc.2 | A_33_P3279861 | 0.947676751 | 2.83E-06 |
| TCONS_00026436 | A_33_P3279861 | 0.959762013 | 7.76E-07 |
| XR_429426.1 | A_33_P3279861 | 0.934306035 | 8.62E-06 |
| XR_429429.1 | A_33_P3279861 | 0.952723454 | 1.72E-06 |
| uc002sti.1 | A_33_P3279861 | 0.924054178 | 1.75E-05 |
| XR_429366.1 | ABCC3 | 0.957744796 | 9.88E-07 |
| XR_426860.1 | ABCC3 | 0.932163401 | 1.01E-05 |
| ENST00000560924.1 | ABCC3 | 0.906864708 | 4.71E-05 |
| TCONS_00022516 | ABCC3 | 0.909518005 | 4.10E-05 |
| ENST00000555918.1 | PDLIM1 | 0.941767041 | 4.78E-06 |
| ENST00000558120.1 | PDLIM1 | 0.922074094 | 1.98E-05 |
| XR_241556.1 | PDLIM1 | 0.905594122 | 5.03E-05 |
| NR_027145.2 | PDLIM1 | 0.957977084 | 9.62E-07 |
| ENST00000447323.1 | PDLIM1 | 0.920292779 | 2.21E-05 |
| TCONS_00018467 | PDLIM1 | 0.939227961 | 5.89E-06 |
| ENST00000420595.1 | PDLIM1 | 0.930316167 | 1.15E-05 |
| TCONS_00018335 | PDLIM1 | 0.942971055 | 4.32E-06 |
| ENST00000451706.2 | PDLIM1 | 0.925346437 | 1.61E-05 |
| uc004ehp.2 | PDLIM1 | 0.922096446 | 1.98E-05 |
| uc022caj.1 | PDLIM1 | 0.923351115 | 1.83E-05 |
| TCONS_00013375 | BLNK | 0.947568425 | 2.86E-06 |
| TCONS_00017180 | BLNK | 0.934868575 | 8.27E-06 |
| NR_022010.1 | MYO5B | 0.938464677 | 6.26E-06 |
| ENST00000555918.1 | PBX1 | 0.90304134 | 5.72E-05 |
| ENST00000558120.1 | PBX1 | 0.906542898 | 4.79E-05 |
| NR_027145.2 | PBX1 | 0.930374634 | 1.15E-05 |
| ENST00000510536.1 | PBX1 | 0.917486513 | 2.62E-05 |
| ENST00000420595.1 | PBX1 | 0.955984669 | 1.21E-06 |
| TCONS_00018335 | PBX1 | 0.94432248 | 3.84E-06 |
| TCONS_00014261 | PBX1 | 0.905862815 | 4.96E-05 |
| ENST00000451706.2 | PBX1 | 0.959820014 | 7.71E-07 |
| uc004ehp.2 | PBX1 | 0.924352285 | 1.72E-05 |
| XR_427724.1 | PBX1 | 0.911526372 | 3.67E-05 |
| uc022caj.1 | PBX1 | 0.946861518 | 3.05E-06 |
| TCONS_00022516 | PBX1 | 0.913642979 | 3.27E-05 |
| ENST00000458479.1 | ZC3H13 | 0.907251007 | 4.62E-05 |
| ENST00000599908.1 | ZC3H13 | 0.900800812 | 6.39E-05 |
| ENST00000602310.1 | ZC3H13 | 0.917760084 | 2.58E-05 |
| ENST00000430228.1 | ZC3H13 | 0.906416948 | 4.82E-05 |
| NR_036530.1 | ZC3H13 | 0.90925027 | 4.16E-05 |
| ENST00000443574.1 | ZC3H13 | -0.940948905 | 5.12E-06 |
| uc002tzb.1 | ZC3H13 | 0.901110169 | 6.30E-05 |
| ENST00000432411.1 | ZC3H13 | 0.93788182 | 6.56E-06 |
| XR_429354.1 | ARL15 | -0.913188482 | 3.35E-05 |
| ENST00000565623.1 | ARL15 | -0.901156323 | 6.28E-05 |
| ENST00000607950.1 | ARL15 | 0.904594427 | 5.29E-05 |
| NR_027145.2 | TPM1 | 0.912193833 | 3.54E-05 |
| ENST00000608442.1 | TPM1 | 0.912048025 | 3.57E-05 |
| ENST00000420595.1 | TPM1 | 0.925159077 | 1.63E-05 |
| ENST00000442008.2 | TPM1 | 0.957236096 | 1.05E-06 |
| ENST00000602325.1 | TPM1 | 0.93886537 | 6.07E-06 |
| LIT2094 | TPM1 | 0.937819777 | 6.59E-06 |
| uc004ehp.2 | TPM1 | 0.903217053 | 5.67E-05 |
| ENST00000424116.2 | TPM1 | 0.939440458 | 5.79E-06 |
| ENST00000607175.1 | TPM1 | 0.936728545 | 7.18E-06 |
| uc022caj.1 | TPM1 | 0.903823979 | 5.50E-05 |
| ENST00000451706.2 | lnc-GTF2E2-1 | 0.906967531 | 4.69E-05 |
| ENST00000555918.1 | HIST1H2BK | 0.902010221 | 6.02E-05 |
| ENST00000518090.1 | HIST1H2BK | 0.912647116 | 3.45E-05 |
| ENST00000608505.1 | HIST1H2BK | 0.939197996 | 5.91E-06 |
| ENST00000558120.1 | HIST1H2BK | 0.917438754 | 2.63E-05 |
| NR_027145.2 | HIST1H2BK | 0.9217933 | 2.02E-05 |
| ENST00000447323.1 | HIST1H2BK | 0.90912863 | 4.18E-05 |
| ENST00000445429.1 | HIST1H2BK | 0.942744075 | 4.40E-06 |
| ENST00000553317.1 | HIST1H2BK | 0.927206005 | 1.42E-05 |
| ENST00000457661.1 | HIST1H2BK | 0.938578477 | 6.21E-06 |
| NR_037616.1 | HIST1H2BK | 0.926302827 | 1.51E-05 |
| ENST00000556936.1 | HIST1H2BK | 0.903115705 | 5.70E-05 |
| ENST00000430620.1 | HIST1H2BK | 0.925520286 | 1.59E-05 |
| ENST00000607175.1 | HIST1H2BK | 0.906217922 | 4.87E-05 |
| ENST00000556996.1 | HIST1H2BK | 0.913013078 | 3.38E-05 |
| TCONS_00011821 | DLX1 | 0.912347546 | 3.51E-05 |
| TCONS_00000467 | INTS6 | 0.909614758 | 4.08E-05 |
| TCONS_00024392 | INTS6 | 0.900364547 | 6.53E-05 |
| ENST00000518090.1 | ALOX12 | 0.915880164 | 2.88E-05 |
| LIT2094 | ALOX12 | 0.904032133 | 5.45E-05 |
| NR_037928.1 | ALOX12 | 0.901273358 | 6.25E-05 |
| ENST00000424116.2 | ALOX12 | 0.929619054 | 1.21E-05 |
| ENST00000555918.1 | CAV2 | 0.915630494 | 2.92E-05 |
| ENST00000510536.1 | CAV2 | 0.918511554 | 2.46E-05 |
| TCONS_00001806 | CAV2 | 0.926927894 | 1.45E-05 |
| ENST00000451706.2 | CAV2 | 0.977077964 | 4.80E-08 |
| XR_427724.1 | CAV2 | 0.933336975 | 9.26E-06 |
| uc021thc.2 | LOC102724332 | 0.934802876 | 8.31E-06 |
| TCONS_00026436 | LOC102724332 | 0.942957725 | 4.32E-06 |
| XR_429426.1 | LOC102724332 | 0.962307214 | 5.62E-07 |
| XR_429429.1 | LOC102724332 | 0.975996246 | 6.03E-08 |
| uc002sti.1 | LOC102724332 | 0.989945571 | 7.96E-10 |
| HIT000078556_03 | A_33_P3289246 | 0.955852464 | 1.23E-06 |
| uc021thc.2 | A_33_P3289246 | 0.929442582 | 1.22E-05 |
| XR_429426.1 | A_33_P3289246 | 0.969749101 | 1.90E-07 |
| NR_026800.1 | A_33_P3289246 | 0.933011717 | 9.49E-06 |
| XR_429429.1 | A_33_P3289246 | 0.948836706 | 2.53E-06 |
| RNA147249\|p0353_imsncRNA771 | BLK | 0.940522625 | 5.30E-06 |
| RNA147248\|p0352_imsncRNA771 | BLK | 0.930626933 | 1.13E-05 |
| TCONS_00001176 | BLK | 0.904440481 | 5.34E-05 |
| XR_426860.1 | lnc-PDZD8-1 | 0.90148771 | 6.18E-05 |
| TCONS_00022516 | lnc-PDZD8-1 | 0.941181463 | 5.02E-06 |
| ENST00000608505.1 | GNG11 | 0.901860603 | 6.07E-05 |
| ENST00000558120.1 | GNG11 | 0.956801859 | 1.10E-06 |
| LIT2061 | GNG11 | 0.936691446 | 7.20E-06 |
| NR_027145.2 | GNG11 | 0.944188389 | 3.88E-06 |
| ENST00000554253.1 | GNG11 | 0.920665108 | 2.16E-05 |
| ENST00000420595.1 | GNG11 | 0.900088048 | 6.62E-05 |
| ENST00000442008.2 | GNG11 | 0.931351512 | 1.07E-05 |
| XR_426860.1 | GNG11 | 0.908166682 | 4.40E-05 |
| ENST00000553317.1 | GNG11 | 0.929878874 | 1.19E-05 |
| ENST00000602325.1 | GNG11 | 0.938175224 | 6.41E-06 |
| LIT2094 | GNG11 | 0.90724116 | 4.62E-05 |
| NR_037928.1 | GNG11 | 0.91497791 | 3.03E-05 |
| ENST00000457661.1 | GNG11 | 0.916717656 | 2.74E-05 |
| NR_037616.1 | GNG11 | 0.909763481 | 4.04E-05 |
| ENST00000556936.1 | GNG11 | 0.913945295 | 3.21E-05 |
| ENST00000424116.2 | GNG11 | 0.969439148 | 1.99E-07 |
| ENST00000607175.1 | GNG11 | 0.958950886 | 8.57E-07 |
| TCONS_00022516 | GNG11 | 0.967079759 | 2.88E-07 |
| uc021thc.2 | A_24_P605563 | 0.935648988 | 7.80E-06 |
| TCONS_00026436 | A_24_P605563 | 0.912996602 | 3.39E-05 |
| XR_429426.1 | A_24_P605563 | 0.940829013 | 5.17E-06 |
| XR_429429.1 | A_24_P605563 | 0.979955626 | 2.46E-08 |
| uc002sti.1 | A_24_P605563 | 0.986318792 | 3.69E-09 |
| HIT000078556_03 | LOC102725284 | 0.91912312 | 2.38E-05 |
| TCONS_00013375 | LOC102725284 | 0.904751935 | 5.25E-05 |
| XR_241556.1 | NT5M | 0.967507132 | 2.70E-07 |
| ENST00000561134.1 | NT5M | 0.916836247 | 2.72E-05 |
| ENST00000602325.1 | NT5M | 0.915604779 | 2.92E-05 |
| LIT2094 | NT5M | 0.917794827 | 2.57E-05 |
| NR_037928.1 | NT5M | 0.930634215 | 1.12E-05 |
| ENST00000424116.2 | NT5M | 0.92233503 | 1.95E-05 |
| TCONS_00018268 | WDFY4 | -0.922562453 | 1.92E-05 |
| LIT1273 | WDFY4 | -0.939826997 | 5.61E-06 |
| TCONS_00000305 | WDFY4 | -0.911018447 | 3.78E-05 |
| ENST00000512716.1 | WDFY4 | 0.905618064 | 5.02E-05 |
| ENST00000554253.1 | CRAT | 0.91945512 | 2.33E-05 |
| ENST00000420595.1 | CRAT | 0.906668388 | 4.76E-05 |
| ENST00000442008.2 | CRAT | 0.935584502 | 7.83E-06 |
| ENST00000553317.1 | CRAT | 0.903384154 | 5.63E-05 |
| ENST00000602325.1 | CRAT | 0.917895419 | 2.56E-05 |
| ENST00000451706.2 | CRAT | 0.917872218 | 2.56E-05 |
| LIT2094 | CRAT | 0.942683347 | 4.42E-06 |
| NR_037928.1 | CRAT | 0.926866283 | 1.46E-05 |
| ENST00000457661.1 | CRAT | 0.903777062 | 5.52E-05 |
| ENST00000506718.1 | CRAT | 0.900552072 | 6.47E-05 |
| ENST00000424116.2 | CRAT | 0.921833025 | 2.01E-05 |
| ENST00000607175.1 | CRAT | 0.921849565 | 2.01E-05 |
| ENST00000610185.1 | CRAT | 0.905566867 | 5.04E-05 |
| ENST00000558120.1 | SLC3A1 | 0.903501461 | 5.59E-05 |
| NR_037928.1 | SLC3A1 | 0.902444477 | 5.90E-05 |
| ENST00000560924.1 | SLC3A1 | 0.941874137 | 4.74E-06 |
| ENST00000560296.1 | SLC3A1 | 0.91026823 | 3.93E-05 |
| ENST00000428088.1 | LOC101928932 | 0.987674462 | 2.19E-09 |
| ENST00000478759.1 | LOC101928932 | 0.990662316 | 5.50E-10 |
| XR_245347.1 | LOC101928932 | 0.990965188 | 4.67E-10 |
| TCONS_00006776 | LOC101928932 | 0.989419008 | 1.03E-09 |
| ENST00000430694.1 | PNOC | 0.916493525 | 2.78E-05 |
| RNA147248\|p0352_imsncRNA771 | PNOC | 0.900596641 | 6.46E-05 |
| ENST00000558120.1 | SH3TC2 | 0.914715653 | 3.07E-05 |
| LIT2061 | SH3TC2 | 0.92784545 | 1.36E-05 |
| NR_027145.2 | SH3TC2 | 0.912941096 | 3.40E-05 |
| ENST00000420595.1 | SH3TC2 | 0.938048623 | 6.47E-06 |
| TCONS_00018335 | SH3TC2 | 0.901264726 | 6.25E-05 |
| LIT2094 | SH3TC2 | 0.903857701 | 5.49E-05 |
| ENST00000506718.1 | SH3TC2 | 0.901369277 | 6.22E-05 |
| ENST00000424116.2 | SH3TC2 | 0.912669364 | 3.45E-05 |
| ENST00000607175.1 | SH3TC2 | 0.915277725 | 2.98E-05 |
| uc022caj.1 | SH3TC2 | 0.907837685 | 4.48E-05 |
| TCONS_00022516 | SH3TC2 | 0.914664589 | 3.08E-05 |
| XR_429426.1 | A_33_P3289248 | 0.943626212 | 4.08E-06 |
| XR_429429.1 | A_33_P3289248 | 0.902721603 | 5.82E-05 |
| XR_429426.1 | PPP1R14A | 0.903632748 | 5.56E-05 |
| uc004ehp.2 | PPP1R14A | 0.901207465 | 6.27E-05 |
| uc022caj.1 | PPP1R14A | 0.930464354 | 1.14E-05 |
| HIT000078556_03 | IGLL5 | 0.900054152 | 6.63E-05 |
| uc021thc.2 | IGLL5 | 0.926137613 | 1.53E-05 |
| XR_429426.1 | IGLL5 | 0.916470313 | 2.78E-05 |
| XR_429429.1 | IGLL5 | 0.969268722 | 2.05E-07 |
| uc002sti.1 | IGLL5 | 0.980483901 | 2.16E-08 |
| ENST00000555918.1 | DNM3 | 0.932734266 | 9.68E-06 |
| ENST00000558120.1 | DNM3 | 0.944189456 | 3.88E-06 |
| NR_027145.2 | DNM3 | 0.97801557 | 3.90E-08 |
| ENST00000447323.1 | DNM3 | 0.9182387 | 2.51E-05 |
| TCONS_00018467 | DNM3 | 0.913318254 | 3.33E-05 |
| ENST00000445429.1 | DNM3 | 0.914583132 | 3.10E-05 |
| ENST00000467896.2 | DNM3 | 0.945664773 | 3.40E-06 |
| ENST00000592441.1 | DNM3 | 0.913541427 | 3.29E-05 |
| XR_426860.1 | DNM3 | 0.905527889 | 5.05E-05 |
| ENST00000553317.1 | DNM3 | 0.935636906 | 7.80E-06 |
| ENST00000457661.1 | DNM3 | 0.920053808 | 2.25E-05 |
| NR_037616.1 | DNM3 | 0.939286776 | 5.86E-06 |
| ENST00000556936.1 | DNM3 | 0.909979936 | 4.00E-05 |
| ENST00000430620.1 | DNM3 | 0.948561644 | 2.60E-06 |
| ENST00000424116.2 | DNM3 | 0.902395869 | 5.91E-05 |
| ENST00000607175.1 | DNM3 | 0.94393096 | 3.97E-06 |
| XR_427724.1 | DNM3 | 0.913866613 | 3.23E-05 |
| ENST00000556996.1 | DNM3 | 0.923355338 | 1.83E-05 |
| ENST00000561134.1 | SH3BGRL2 | 0.93852253 | 6.23E-06 |
| XR_426860.1 | SH3BGRL2 | 0.900398823 | 6.52E-05 |
| ENST00000602325.1 | SH3BGRL2 | 0.959082384 | 8.43E-07 |
| ENST00000424116.2 | SH3BGRL2 | 0.931128229 | 1.09E-05 |
| ENST00000607175.1 | SH3BGRL2 | 0.920514178 | 2.18E-05 |
| ENST00000503611.1 | TMEM144 | 0.948130944 | 2.71E-06 |
| ENST00000514571.1 | AVIL | -0.910387395 | 3.91E-05 |
| ENST00000573950.1 | AVIL | -0.940634199 | 5.25E-06 |
| ENST00000430694.1 | KLHL14 | 0.925139411 | 1.63E-05 |
| NR_026800.1 | KLHL14 | 0.92486751 | 1.66E-05 |
| NR_037928.1 | H2AFJ | 0.931382412 | 1.07E-05 |
| ENST00000518090.1 | TFPI | 0.922690234 | 1.91E-05 |
| ENST00000558120.1 | TFPI | 0.912439561 | 3.49E-05 |
| NR_027145.2 | TFPI | 0.931714328 | 1.04E-05 |
| ENST00000592441.1 | TFPI | 0.931243938 | 1.08E-05 |
| XR_426860.1 | TFPI | 0.907085695 | 4.66E-05 |
| ENST00000412276.1 | TFPI | 0.927586122 | 1.39E-05 |
| ENST00000560924.1 | TFPI | 0.901022981 | 6.32E-05 |
| ENST00000556936.1 | TFPI | 0.911727685 | 3.63E-05 |
| ENST00000556996.1 | TFPI | 0.943108753 | 4.26E-06 |
| uc003kmx.1 | CPB2 | 0.930242106 | 1.16E-05 |
| ENST00000584683.1 | CPB2 | 0.916703957 | 2.74E-05 |
| TCONS_00026998 | CPB2 | 0.903553732 | 5.58E-05 |
| RNA147028\|p0132_imsncRNA212 | CPB2 | 0.937814867 | 6.59E-06 |
| ENST00000609413.1 | CPB2 | 0.925465464 | 1.60E-05 |
| RNA147577\|p0681_imsncRNA442 | CPB2 | 0.929312495 | 1.23E-05 |
| ENST00000413818.2 | CPB2 | 0.914835263 | 3.05E-05 |
| ENST00000586885.1 | CPB2 | 0.963415601 | 4.85E-07 |
| TCONS_00024308 | CPB2 | 0.903295989 | 5.65E-05 |
| ENST00000552061.1 | CPB2 | 0.900277981 | 6.56E-05 |
| ENST00000432411.1 | CPB2 | 0.909862873 | 4.02E-05 |
| ENST00000442008.2 | HOMER3 | 0.940076314 | 5.50E-06 |
| ENST00000602325.1 | HOMER3 | 0.908868316 | 4.24E-05 |
| LIT2094 | HOMER3 | 0.955737763 | 1.24E-06 |
| NR_037928.1 | HOMER3 | 0.964750589 | 4.04E-07 |
| ENST00000424116.2 | HOMER3 | 0.931157952 | 1.08E-05 |
| ENST00000610185.1 | HOMER3 | 0.904346734 | 5.36E-05 |
| ENST00000421937.3 | ATF7 | -0.906453184 | 4.81E-05 |
| LIT2094 | GSTO1 | 0.901866485 | 6.07E-05 |
| HIT000078556_03 | A_33_P3331172 | 0.921002516 | 2.12E-05 |
| uc021thc.2 | A_33_P3331172 | 0.97046972 | 1.68E-07 |
| TCONS_00026436 | A_33_P3331172 | 0.934742478 | 8.35E-06 |
| XR_429426.1 | A_33_P3331172 | 0.977357904 | 4.51E-08 |
| XR_429429.1 | A_33_P3331172 | 0.967335882 | 2.77E-07 |
| uc002sti.1 | A_33_P3331172 | 0.919903414 | 2.27E-05 |
| uc021vkt.1 | A_33_P3331172 | 0.925674684 | 1.58E-05 |
| ENST00000431928.1 | FSCN1 | -0.931887743 | 1.03E-05 |
| ENST00000508021.1 | SNCA | 0.978804706 | 3.25E-08 |
| RNA147574\|p0678_imsncRNA439 | RNF208 | -0.946155266 | 3.26E-06 |
| ENST00000608442.1 | RNF208 | 0.948151575 | 2.70E-06 |
| ENST00000467896.2 | RNF208 | 0.926301775 | 1.51E-05 |
| ENST00000513153.1 | RNF208 | 0.9295411 | 1.21E-05 |
| ENST00000607175.1 | RNF208 | 0.901561077 | 6.16E-05 |
| ENST00000558120.1 | LEPROT | 0.910765723 | 3.83E-05 |
| NR_027145.2 | LEPROT | 0.922514041 | 1.93E-05 |
| ENST00000447323.1 | LEPROT | 0.920845 | 2.14E-05 |
| LIT2094 | LEPROT | 0.906968947 | 4.69E-05 |
| NR_037928.1 | LEPROT | 0.922391406 | 1.94E-05 |
| uc004ehp.2 | LEPROT | 0.917841386 | 2.57E-05 |
| ENST00000561559.1 | STAB1 | 0.941842966 | 4.75E-06 |
| ENST00000608505.1 | TFPI | 0.923267134 | 1.84E-05 |
| ENST00000558120.1 | TFPI | 0.921575889 | 2.05E-05 |
| LIT2061 | TFPI | 0.921952393 | 2.00E-05 |
| NR_027145.2 | TFPI | 0.902971941 | 5.74E-05 |
| ENST00000608442.1 | TFPI | 0.920667373 | 2.16E-05 |
| ENST00000554253.1 | TFPI | 0.901862297 | 6.07E-05 |
| ENST00000553317.1 | TFPI | 0.926880493 | 1.45E-05 |
| NR_037928.1 | TFPI | 0.908709229 | 4.28E-05 |
| ENST00000424116.2 | TFPI | 0.958069953 | 9.51E-07 |
| ENST00000607175.1 | TFPI | 0.971769335 | 1.35E-07 |
| TCONS_00010294 | lnc-CHADL-1 | 0.911902259 | 3.60E-05 |
| uc022caj.1 | lnc-CHADL-1 | 0.907754946 | 4.50E-05 |
| ENST00000441316.1 | lnc-CHADL-1 | 0.924427384 | 1.71E-05 |
| TCONS_00007196 | KLHL6 | 0.908888234 | 4.24E-05 |
| HIT000078556_03 | A_33_P3247639 | 0.955011879 | 1.35E-06 |
| uc021thc.2 | A_33_P3247639 | 0.902832464 | 5.78E-05 |
| XR_429426.1 | A_33_P3247639 | 0.919883275 | 2.27E-05 |
| NR_026800.1 | A_33_P3247639 | 0.934389378 | 8.57E-06 |
| XR_429429.1 | A_33_P3247639 | 0.909110865 | 4.19E-05 |
| ENST00000454312.1 | A_21_P0001243 | -0.944913431 | 3.64E-06 |
| NR_036530.1 | A_21_P0001243 | 0.902538809 | 5.87E-05 |
| ENST00000417976.1 | A_21_P0001243 | 0.903969909 | 5.46E-05 |
| ENST00000558120.1 | PDGFA | 0.910471868 | 3.89E-05 |
| LIT2061 | PDGFA | 0.915968211 | 2.86E-05 |
| ENST00000424116.2 | PDGFA | 0.926662876 | 1.48E-05 |
| TCONS_00022516 | PDGFA | 0.970870483 | 1.57E-07 |
| TCONS_00011821 | LAMTOR1 | -0.921157481 | 2.10E-05 |
| NR_037928.1 | LAMTOR1 | 0.925125282 | 1.63E-05 |
| ENST00000421937.3 | LAMTOR1 | -0.90315353 | 5.69E-05 |
| ENST00000530194.1 | A_33_P3346881 | 0.900856279 | 6.37E-05 |
| NR_110177.1 | NCK1-AS1 | 0.950128884 | 2.23E-06 |
| XR_430056.1 | MEIS1 | 0.939360353 | 5.83E-06 |
| ENST00000558120.1 | PRKAR2B | 0.912810707 | 3.42E-05 |
| LIT2061 | PRKAR2B | 0.913124742 | 3.36E-05 |
| ENST00000420595.1 | PRKAR2B | 0.921543272 | 2.05E-05 |
| ENST00000442008.2 | PRKAR2B | 0.930475015 | 1.14E-05 |
| XR_426860.1 | PRKAR2B | 0.906994686 | 4.68E-05 |
| ENST00000602325.1 | PRKAR2B | 0.948302887 | 2.67E-06 |
| LIT2094 | PRKAR2B | 0.906172609 | 4.88E-05 |
| ENST00000424116.2 | PRKAR2B | 0.944898973 | 3.65E-06 |
| ENST00000607175.1 | PRKAR2B | 0.905925896 | 4.95E-05 |
| TCONS_00022516 | PRKAR2B | 0.972445926 | 1.19E-07 |
| ENST00000608505.1 | MPL | 0.916446313 | 2.78E-05 |
| ENST00000607175.1 | MPL | 0.918650239 | 2.44E-05 |
| LIT2094 | NT5C3A | 0.950090293 | 2.24E-06 |
| ENST00000432668.1 | NT5C3A | -0.901149043 | 6.28E-05 |
| TCONS_00001179 | NT5C3A | -0.900676956 | 6.43E-05 |
| ENST00000558120.1 | ZNF185 | 0.943170674 | 4.24E-06 |
| NR_027145.2 | ZNF185 | 0.958802143 | 8.72E-07 |
| ENST00000467896.2 | ZNF185 | 0.905603299 | 5.03E-05 |
| RNA95734\|RNS_816_119 | ZNF185 | 0.907458544 | 4.57E-05 |
| NR_037928.1 | ZNF185 | 0.908567247 | 4.31E-05 |
| ENST00000508021.1 | ZNF185 | 0.910052769 | 3.98E-05 |
| ENST00000424116.2 | ZNF185 | 0.916195045 | 2.82E-05 |
| TCONS_00022516 | ZNF185 | 0.902706549 | 5.82E-05 |
| ENST00000448017.1 | lnc-MBL2-3 | 0.926858919 | 1.46E-05 |
| ENST00000443523.1 | lnc-MBL2-3 | 0.927975319 | 1.35E-05 |
| TCONS_00015115 | lnc-MBL2-3 | 0.926383166 | 1.50E-05 |
| ENST00000555918.1 | GP1BB | 0.914318812 | 3.15E-05 |
| ENST00000558120.1 | GP1BB | 0.936774698 | 7.15E-06 |
| XR_241556.1 | GP1BB | 0.918743986 | 2.43E-05 |
| NR_027145.2 | GP1BB | 0.925930873 | 1.55E-05 |
| ENST00000561134.1 | GP1BB | 0.901449061 | 6.19E-05 |
| TCONS_00018467 | GP1BB | 0.903140965 | 5.70E-05 |
| ENST00000420595.1 | GP1BB | 0.917216481 | 2.66E-05 |
| ENST00000442008.2 | GP1BB | 0.92618408 | 1.52E-05 |
| ENST00000602325.1 | GP1BB | 0.912477026 | 3.49E-05 |
| LIT2094 | GP1BB | 0.925450191 | 1.60E-05 |
| NR_037928.1 | GP1BB | 0.960812439 | 6.81E-07 |
| uc004ehp.2 | GP1BB | 0.906658278 | 4.76E-05 |
| ENST00000424116.2 | GP1BB | 0.954590209 | 1.41E-06 |
| ENST00000607175.1 | GP1BB | 0.922774749 | 1.90E-05 |
| uc022caj.1 | GP1BB | 0.915025194 | 3.02E-05 |
| TCONS_00022516 | GP1BB | 0.902796337 | 5.79E-05 |
| HIT000078556_03 | A_24_P357847 | 0.929502198 | 1.22E-05 |
| uc021thc.2 | A_24_P357847 | 0.959805027 | 7.72E-07 |
| TCONS_00026436 | A_24_P357847 | 0.917797428 | 2.57E-05 |
| XR_429426.1 | A_24_P357847 | 0.963288079 | 4.94E-07 |
| XR_429429.1 | A_24_P357847 | 0.982609599 | 1.22E-08 |
| uc002sti.1 | A_24_P357847 | 0.972988524 | 1.08E-07 |
| NR_027145.2 | PBXIP1 | 0.907736156 | 4.50E-05 |
| ENST00000447323.1 | PBXIP1 | 0.904479249 | 5.32E-05 |
| ENST00000467896.2 | PBXIP1 | 0.91205841 | 3.57E-05 |
| ENST00000513153.1 | PBXIP1 | 0.913374055 | 3.32E-05 |
| ENST00000450238.1 | PBXIP1 | 0.937783321 | 6.61E-06 |
| uc004ehp.2 | PBXIP1 | 0.900065089 | 6.62E-05 |
| uc022caj.1 | PBXIP1 | 0.912732719 | 3.44E-05 |
| ENST00000430228.1 | LOC100507144 | 0.904025113 | 5.45E-05 |
| RNA147009\|p0113_imsncRNA187 | LOC100507144 | 0.916433441 | 2.79E-05 |
| RNA146910\|p0014_imsncRNA45 | LOC100507144 | 0.910545906 | 3.88E-05 |
| TCONS_00026998 | FAM47A | 0.945497604 | 3.46E-06 |
| ENST00000426704.1 | FAM47A | 0.924127767 | 1.74E-05 |
| XR_427192.1 | ENKUR | 0.905051075 | 5.17E-05 |
| ENST00000510536.1 | ENKUR | 0.911463973 | 3.69E-05 |
| ENST00000467896.2 | ENKUR | 0.934459337 | 8.53E-06 |
| ENST00000458314.1 | ENKUR | 0.905869772 | 4.96E-05 |
| ENST00000513153.1 | ENKUR | 0.952769633 | 1.71E-06 |
| ENST00000430620.1 | ENKUR | 0.908401484 | 4.35E-05 |
| TCONS_00026998 | A_33_P3272668 | 0.913304612 | 3.33E-05 |
| ENST00000573861.1 | A_33_P3272668 | 0.910375487 | 3.91E-05 |
| ENST00000527067.1 | A_33_P3272668 | 0.935764025 | 7.73E-06 |
| TCONS_00024308 | A_33_P3272668 | 0.913276969 | 3.33E-05 |
| ENST00000449457.1 | A_33_P3272668 | 0.902649283 | 5.84E-05 |
| ENST00000439622.1 | RAD23B | -0.907192445 | 4.63E-05 |
| NR_027034.1 | RAD23B | -0.918148153 | 2.52E-05 |
| uc003kmx.1 | RAD23B | -0.914065625 | 3.19E-05 |
| ENST00000505637.1 | RAD23B | -0.903379655 | 5.63E-05 |
| ENST00000548170.1 | RAD23B | 0.906662895 | 4.76E-05 |
| TCONS_00026223 | RAD23B | -0.923468577 | 1.82E-05 |
| ENST00000527067.1 | RAD23B | -0.935061402 | 8.15E-06 |
| ENST00000413818.2 | RAD23B | -0.930731152 | 1.12E-05 |
| TCONS_00005965 | RAD23B | 0.939079197 | 5.96E-06 |
| ENST00000421937.3 | RAD23B | -0.910241008 | 3.94E-05 |
| ENST00000432411.1 | RAD23B | -0.906841083 | 4.72E-05 |
| RNA146910\|p0014_imsncRNA45 | RAD23B | -0.913694324 | 3.26E-05 |
| TCONS_00006023 | RAD23B | 0.912904667 | 3.40E-05 |
| ENST00000558120.1 | LIMS1 | 0.956319974 | 1.16E-06 |
| XR_241556.1 | LIMS1 | 0.91111043 | 3.76E-05 |
| NR_027145.2 | LIMS1 | 0.945349127 | 3.50E-06 |
| TCONS_00018467 | LIMS1 | 0.90746837 | 4.57E-05 |
| ENST00000420595.1 | LIMS1 | 0.911240513 | 3.73E-05 |
| ENST00000442008.2 | LIMS1 | 0.94117005 | 5.03E-06 |
| ENST00000602325.1 | LIMS1 | 0.914270309 | 3.15E-05 |
| LIT2094 | LIMS1 | 0.936388637 | 7.37E-06 |
| NR_037928.1 | LIMS1 | 0.936962586 | 7.05E-06 |
| ENST00000424116.2 | LIMS1 | 0.94489271 | 3.65E-06 |
| ENST00000607175.1 | LIMS1 | 0.922064364 | 1.98E-05 |
| TCONS_00022516 | LIMS1 | 0.936928082 | 7.07E-06 |
| uc010vhc.3 | THSD1 | 0.928565956 | 1.30E-05 |
| ENST00000558120.1 | CTTN | 0.926985224 | 1.44E-05 |
| LIT2061 | CTTN | 0.909996227 | 3.99E-05 |
| NR_027145.2 | CTTN | 0.913194704 | 3.35E-05 |
| ENST00000554253.1 | CTTN | 0.923180865 | 1.85E-05 |
| XR_426860.1 | CTTN | 0.935827362 | 7.69E-06 |
| ENST00000553317.1 | CTTN | 0.920762764 | 2.15E-05 |
| ENST00000602325.1 | CTTN | 0.937916717 | 6.54E-06 |
| ENST00000457661.1 | CTTN | 0.918092776 | 2.53E-05 |
| ENST00000556936.1 | CTTN | 0.913524275 | 3.29E-05 |
| ENST00000424116.2 | CTTN | 0.925593728 | 1.58E-05 |
| ENST00000607175.1 | CTTN | 0.927984252 | 1.35E-05 |
| TCONS_00022516 | CTTN | 0.951274036 | 1.99E-06 |
| HIT000078556_03 | A_33_P3281444 | 0.980798058 | 1.99E-08 |
| ENST00000421937.3 | MAPK10 | -0.915694507 | 2.91E-05 |
| ENST00000467896.2 | PRDX6 | 0.925415934 | 1.60E-05 |
| ENST00000608505.1 | SMIM5 | 0.914566376 | 3.10E-05 |
| ENST00000554253.1 | SMIM5 | 0.904409092 | 5.34E-05 |
| ENST00000553317.1 | SMIM5 | 0.908237411 | 4.39E-05 |
| ENST00000457661.1 | SMIM5 | 0.942949087 | 4.32E-06 |
| ENST00000556936.1 | SMIM5 | 0.9549795 | 1.35E-06 |
| ENST00000430620.1 | SMIM5 | 0.902471966 | 5.89E-05 |
| ENST00000458479.1 | AKTIP | 0.934318531 | 8.62E-06 |
| NR_022010.1 | AKTIP | 0.966100969 | 3.33E-07 |
| LIT1273 | AKTIP | 0.906390067 | 4.83E-05 |
| ENST00000432411.1 | AKTIP | 0.922304663 | 1.95E-05 |
| HIT000078556_03 | A_24_P813550 | 0.949991025 | 2.26E-06 |
| uc021thc.2 | A_24_P813550 | 0.946429371 | 3.17E-06 |
| XR_429426.1 | A_24_P813550 | 0.948860931 | 2.53E-06 |
| XR_429429.1 | A_24_P813550 | 0.956220356 | 1.18E-06 |
| uc002sti.1 | A_24_P813550 | 0.90004327 | 6.63E-05 |
| uc021vkt.1 | A_24_P813550 | 0.912940771 | 3.40E-05 |
| ENST00000440004.1 | PLXDC1 | 0.921098785 | 2.11E-05 |
| uc001ttj.1 | PLXDC1 | 0.901711547 | 6.11E-05 |
| NR_027145.2 | TPM1 | 0.921116993 | 2.10E-05 |
| ENST00000608442.1 | TPM1 | 0.902544382 | 5.87E-05 |
| ENST00000561134.1 | TPM1 | 0.911065369 | 3.77E-05 |
| ENST00000420595.1 | TPM1 | 0.917445874 | 2.63E-05 |
| ENST00000442008.2 | TPM1 | 0.946575001 | 3.13E-06 |
| ENST00000602325.1 | TPM1 | 0.939716274 | 5.66E-06 |
| LIT2094 | TPM1 | 0.920688275 | 2.16E-05 |
| uc004ehp.2 | TPM1 | 0.907517166 | 4.55E-05 |
| ENST00000424116.2 | TPM1 | 0.939482609 | 5.77E-06 |
| ENST00000607175.1 | TPM1 | 0.935481858 | 7.90E-06 |
| uc022caj.1 | TPM1 | 0.905010588 | 5.18E-05 |
| HIT000078556_03 | A_24_P608268 | 0.957218745 | 1.05E-06 |
| uc021thc.2 | A_24_P608268 | 0.924991853 | 1.65E-05 |
| XR_429426.1 | A_24_P608268 | 0.954717301 | 1.39E-06 |
| NR_026800.1 | A_24_P608268 | 0.931726298 | 1.04E-05 |
| XR_429429.1 | A_24_P608268 | 0.94643319 | 3.17E-06 |
| HIT000078556_03 | A_33_P3331233 | 0.951686159 | 1.91E-06 |
| ENST00000558120.1 | LY6G6F | 0.919151831 | 2.37E-05 |
| XR_241556.1 | LY6G6F | 0.940167104 | 5.46E-06 |
| NR_027145.2 | LY6G6F | 0.913907616 | 3.22E-05 |
| ENST00000561134.1 | LY6G6F | 0.924716022 | 1.68E-05 |
| ENST00000602325.1 | LY6G6F | 0.946214465 | 3.24E-06 |
| NR_037928.1 | LY6G6F | 0.91276737 | 3.43E-05 |
| ENST00000424116.2 | LY6G6F | 0.954296919 | 1.45E-06 |
| ENST00000607175.1 | LY6G6F | 0.927186014 | 1.43E-05 |
| TCONS_00022516 | LY6G6F | 0.926704045 | 1.47E-05 |
| NR_026880.1 | SH2B2 | -0.931566427 | 1.05E-05 |
| ENST00000426704.1 | SH2B2 | -0.928660909 | 1.29E-05 |
| ENST00000583262.1 | SH2B2 | -0.921830181 | 2.01E-05 |
| XR_428131.1 | SH2B2 | -0.92000329 | 2.25E-05 |
| uc001ttj.1 | SH2B2 | -0.919723805 | 2.29E-05 |
| HIT000078556_03 | A_24_P315941 | 0.969177244 | 2.08E-07 |
| XR_429429.1 | A_24_P315941 | 0.92708781 | 1.43E-05 |
| ENST00000416080.1 | XLOC_l2_013153 | 0.932928004 | 9.55E-06 |
| uc003qls.2 | XLOC_l2_013153 | 0.954271949 | 1.46E-06 |
| ENST00000555918.1 | CXCL5 | 0.902022281 | 6.02E-05 |
| ENST00000558120.1 | CXCL5 | 0.90816915 | 4.40E-05 |
| ENST00000554253.1 | CXCL5 | 0.939231262 | 5.89E-06 |
| ENST00000442008.2 | CXCL5 | 0.914340452 | 3.14E-05 |
| ENST00000553317.1 | CXCL5 | 0.914480527 | 3.12E-05 |
| ENST00000451706.2 | CXCL5 | 0.914252916 | 3.16E-05 |
| LIT2094 | CXCL5 | 0.90327948 | 5.66E-05 |
| NR_037928.1 | CXCL5 | 0.919968508 | 2.26E-05 |
| ENST00000506718.1 | CXCL5 | 0.929689359 | 1.20E-05 |
| ENST00000607175.1 | CXCL5 | 0.926470922 | 1.49E-05 |
| ENST00000608505.1 | PTGS1 | 0.916356981 | 2.80E-05 |
| ENST00000558120.1 | PTGS1 | 0.908199689 | 4.39E-05 |
| XR_241556.1 | PTGS1 | 0.939322177 | 5.85E-06 |
| ENST00000561134.1 | PTGS1 | 0.911842498 | 3.61E-05 |
| ENST00000442008.2 | PTGS1 | 0.903884416 | 5.49E-05 |
| ENST00000602325.1 | PTGS1 | 0.936632141 | 7.23E-06 |
| LIT2094 | PTGS1 | 0.925048907 | 1.64E-05 |
| NR_037928.1 | PTGS1 | 0.952160569 | 1.82E-06 |
| ENST00000424116.2 | PTGS1 | 0.982987584 | 1.09E-08 |
| ENST00000607175.1 | PTGS1 | 0.929267455 | 1.24E-05 |
| ENST00000602310.1 | SLC26A3 | 0.911161036 | 3.75E-05 |
| ENST00000420595.1 | ST3GAL3 | 0.919176283 | 2.37E-05 |
| TCONS_00018335 | ST3GAL3 | 0.908543281 | 4.31E-05 |
| ENST00000451706.2 | ST3GAL3 | 0.923924348 | 1.76E-05 |
| LIT2094 | ST3GAL3 | 0.951030261 | 2.04E-06 |
| ENST00000437721.1 | ST3GAL3 | -0.902370721 | 5.92E-05 |
| ENST00000505267.2 | ST3GAL3 | -0.906245657 | 4.87E-05 |
| ENST00000605136.1 | SEPT10 | -0.911878395 | 3.60E-05 |
| ENST00000608241.1 | SEPT10 | 0.914350377 | 3.14E-05 |
| uc002sti.1 | A_33_P3351180 | 0.917342113 | 2.64E-05 |
| ENST00000608995.1 | AKR1C1 | 0.939976357 | 5.55E-06 |
| uc009wka.2 | AKTIP | 0.905410986 | 5.08E-05 |
| NR_026880.1 | AKTIP | 0.900225487 | 6.57E-05 |
| XR_242712.2 | AKTIP | 0.928114185 | 1.34E-05 |
| ENST00000467896.2 | ARMCX6 | 0.900230052 | 6.57E-05 |
| uc002vvv.3 | HOXB2 | 0.932847577 | 9.60E-06 |
| ASO1647 | ZAK | 0.903418012 | 5.62E-05 |
| ENST00000555918.1 | EHD3 | 0.939515887 | 5.76E-06 |
| ENST00000558120.1 | EHD3 | 0.95254267 | 1.75E-06 |
| NR_027145.2 | EHD3 | 0.925574322 | 1.59E-05 |
| TCONS_00018467 | EHD3 | 0.932765505 | 9.66E-06 |
| XR_429366.1 | EHD3 | 0.954784002 | 1.38E-06 |
| ENST00000560924.1 | EHD3 | 0.91024819 | 3.94E-05 |
| uc022caj.1 | EHD3 | 0.91621351 | 2.82E-05 |
| TCONS_00022516 | EHD3 | 0.93033902 | 1.15E-05 |
| ENST00000558120.1 | SIAE | 0.922605688 | 1.92E-05 |
| XR_241556.1 | SIAE | 0.904846377 | 5.23E-05 |
| NR_027145.2 | SIAE | 0.920437428 | 2.19E-05 |
| ENST00000561134.1 | SIAE | 0.962925112 | 5.18E-07 |
| ENST00000442008.2 | SIAE | 0.93238417 | 9.93E-06 |
| ENST00000602325.1 | SIAE | 0.946979042 | 3.02E-06 |
| LIT2094 | SIAE | 0.901884888 | 6.06E-05 |
| NR_037928.1 | SIAE | 0.925252489 | 1.62E-05 |
| ENST00000424116.2 | SIAE | 0.96079088 | 6.83E-07 |
| ENST00000607175.1 | SIAE | 0.942378899 | 4.54E-06 |
| TCONS_00022516 | SIAE | 0.900381613 | 6.52E-05 |
| ENST00000434081.1 | DCBLD2 | 0.921952513 | 2.00E-05 |
| ENST00000535511.1 | TTLL7 | 0.938451262 | 6.27E-06 |
| TCONS_00018335 | TTLL7 | 0.91739631 | 2.63E-05 |
| ENST00000451706.2 | TTLL7 | 0.917117015 | 2.68E-05 |
| uc004ehp.2 | TTLL7 | 0.910504973 | 3.88E-05 |
| TCONS_00024392 | TTLL7 | -0.915321426 | 2.97E-05 |
| uc003kmx.1 | FAM178A | 0.932475209 | 9.86E-06 |
| ENST00000573861.1 | FAM178A | 0.929147288 | 1.25E-05 |
| ENST00000527067.1 | FAM178A | 0.951685345 | 1.91E-06 |
| TCONS_00024308 | FAM178A | 0.934695092 | 8.38E-06 |
| ENST00000430694.1 | GNG7 | 0.9372419 | 6.90E-06 |
| NR_026800.1 | GNG7 | 0.916630083 | 2.75E-05 |
| ENST00000457856.1 | GNG7 | -0.908220214 | 4.39E-05 |
| uc021thc.2 | A_33_P3422298 | 0.934393691 | 8.57E-06 |
| XR_429426.1 | A_33_P3422298 | 0.922741274 | 1.90E-05 |
| XR_429429.1 | A_33_P3422298 | 0.975672166 | 6.44E-08 |
| uc002sti.1 | A_33_P3422298 | 0.987516463 | 2.34E-09 |
| ENST00000429328.2 | FRMD3 | 0.913084888 | 3.37E-05 |
| ENST00000430620.1 | FRMD3 | 0.911671368 | 3.65E-05 |
| XR_241556.1 | RNF11 | 0.955868647 | 1.22E-06 |
| ENST00000561134.1 | RNF11 | 0.921330682 | 2.08E-05 |
| ENST00000420595.1 | RNF11 | 0.929623908 | 1.21E-05 |
| ENST00000602325.1 | RNF11 | 0.947695754 | 2.82E-06 |
| LIT2094 | RNF11 | 0.926810731 | 1.46E-05 |
| NR_037928.1 | RNF11 | 0.917471411 | 2.62E-05 |
| ENST00000424116.2 | RNF11 | 0.959305894 | 8.21E-07 |
| TCONS_00022516 | RNF11 | 0.922086283 | 1.98E-05 |
| ENST00000431928.1 | ZMYND10 | 0.947874283 | 2.78E-06 |
| ENST00000548170.1 | ZMYND10 | -0.926412828 | 1.50E-05 |
| ENST00000430228.1 | ZMYND10 | 0.901571595 | 6.16E-05 |
| ENST00000528726.1 | ZMYND10 | -0.904485973 | 5.32E-05 |
| int-HOXB3-91 | ZMYND10 | 0.935924495 | 7.63E-06 |
| ENST00000565547.1 | ZMYND10 | -0.958277461 | 9.28E-07 |
| ENST00000605056.1 | NRP2 | -0.934682654 | 8.39E-06 |
| ENST00000559026.1 | NRP2 | 0.904910132 | 5.21E-05 |
| ENST00000446847.1 | NRP2 | -0.93188089 | 1.03E-05 |
| ENST00000430228.1 | SCGB1D2 | 0.925001914 | 1.65E-05 |
| ENST00000609413.1 | SCGB1D2 | 0.913519661 | 3.29E-05 |
| RNA147577\|p0681_imsncRNA442 | SCGB1D2 | 0.933290123 | 9.30E-06 |
| ENST00000432411.1 | SCGB1D2 | 0.910185113 | 3.95E-05 |
| XR_429339.1 | SCGB1D2 | 0.913957481 | 3.21E-05 |
| ENST00000439622.1 | OLFM2 | 0.921678318 | 2.03E-05 |
| ENST00000447019.1 | OLFM2 | 0.905133362 | 5.15E-05 |
| ENST00000426704.1 | OLFM2 | 0.9526259 | 1.74E-06 |
| ENST00000607611.1 | OLFM2 | -0.908938181 | 4.23E-05 |
| uc001ttj.1 | OLFM2 | 0.927951726 | 1.35E-05 |
| XR_241556.1 | ARMC3 | 0.912661215 | 3.45E-05 |
| ENST00000420595.1 | ARMC3 | 0.957927177 | 9.67E-07 |
| TCONS_00018335 | ARMC3 | 0.917408308 | 2.63E-05 |
| ENST00000602325.1 | ARMC3 | 0.933657015 | 9.05E-06 |
| LIT2094 | ARMC3 | 0.94968792 | 2.33E-06 |
| NR_037928.1 | ARMC3 | 0.911093514 | 3.76E-05 |
| ENST00000424116.2 | ARMC3 | 0.930777843 | 1.11E-05 |
| TCONS_00022516 | ARMC3 | 0.921923276 | 2.00E-05 |
| ENST00000558120.1 | PEAR1 | 0.941163655 | 5.03E-06 |
| LIT2061 | PEAR1 | 0.930267533 | 1.15E-05 |
| NR_027145.2 | PEAR1 | 0.939545502 | 5.74E-06 |
| ENST00000608442.1 | PEAR1 | 0.918231676 | 2.51E-05 |
| ENST00000554253.1 | PEAR1 | 0.92791934 | 1.36E-05 |
| ENST00000420595.1 | PEAR1 | 0.92831825 | 1.32E-05 |
| ENST00000442008.2 | PEAR1 | 0.925414178 | 1.60E-05 |
| ENST00000553317.1 | PEAR1 | 0.940072934 | 5.50E-06 |
| ENST00000602325.1 | PEAR1 | 0.915811704 | 2.89E-05 |
| LIT2094 | PEAR1 | 0.909830378 | 4.03E-05 |
| NR_037928.1 | PEAR1 | 0.911644261 | 3.65E-05 |
| ENST00000506718.1 | PEAR1 | 0.923180488 | 1.85E-05 |
| ENST00000424116.2 | PEAR1 | 0.943120904 | 4.26E-06 |
| ENST00000607175.1 | PEAR1 | 0.966228336 | 3.27E-07 |
| TCONS_00022516 | PEAR1 | 0.932146013 | 1.01E-05 |
| XR_429429.1 | A_32_P76137 | 0.901670083 | 6.13E-05 |
| HIT000078556_03 | A_33_P3281437 | 0.905945392 | 4.94E-05 |
| uc021thc.2 | A_33_P3281437 | 0.969668627 | 1.92E-07 |
| TCONS_00026436 | A_33_P3281437 | 0.917591128 | 2.60E-05 |
| XR_429426.1 | A_33_P3281437 | 0.942933731 | 4.33E-06 |
| XR_429429.1 | A_33_P3281437 | 0.962289128 | 5.64E-07 |
| uc002sti.1 | A_33_P3281437 | 0.951077581 | 2.03E-06 |
| TCONS_00014580 | A_21_P0005647 | 0.988061994 | 1.87E-09 |
| ENST00000450238.1 | HSPB1 | 0.903480076 | 5.60E-05 |
| TCONS_00012818 | TAS2R60 | 0.938904578 | 6.05E-06 |
| ENST00000608442.1 | A_23_P168092 | 0.936330516 | 7.40E-06 |
| ENST00000602325.1 | A_23_P168092 | 0.923675923 | 1.79E-05 |
| LIT2094 | A_23_P168092 | 0.900632786 | 6.44E-05 |
| ENST00000424116.2 | A_23_P168092 | 0.901415419 | 6.20E-05 |
| ENST00000608560.1 | ADI1 | 0.901169366 | 6.28E-05 |
| ENST00000451340.2 | FAM200B | 0.919622981 | 2.31E-05 |
| ENST00000447019.1 | GTSCR1 | 0.945326798 | 3.51E-06 |
| HIT000078556_03 | A_24_P16004 | 0.916735818 | 2.74E-05 |
| uc021thc.2 | A_24_P16004 | 0.971955767 | 1.30E-07 |
| XR_429426.1 | A_24_P16004 | 0.912420567 | 3.50E-05 |
| XR_429429.1 | A_24_P16004 | 0.965778079 | 3.49E-07 |
| uc001yuj.2 | A_24_P16004 | 0.900025681 | 6.64E-05 |
| uc002sti.1 | A_24_P16004 | 0.95530515 | 1.30E-06 |
| ENST00000555918.1 | SDPR | 0.921329166 | 2.08E-05 |
| ENST00000558120.1 | SDPR | 0.958488062 | 9.05E-07 |
| XR_241556.1 | SDPR | 0.907948746 | 4.45E-05 |
| NR_027145.2 | SDPR | 0.953405253 | 1.60E-06 |
| ENST00000420595.1 | SDPR | 0.909947293 | 4.00E-05 |
| ENST00000442008.2 | SDPR | 0.916275053 | 2.81E-05 |
| XR_426860.1 | SDPR | 0.913887195 | 3.22E-05 |
| ENST00000602325.1 | SDPR | 0.931030624 | 1.09E-05 |
| ENST00000451706.2 | SDPR | 0.928910472 | 1.27E-05 |
| NR_037928.1 | SDPR | 0.912337932 | 3.51E-05 |
| ENST00000418746.1 | SDPR | 0.901008586 | 6.33E-05 |
| ENST00000424116.2 | SDPR | 0.928623766 | 1.29E-05 |
| ENST00000607175.1 | SDPR | 0.911849132 | 3.61E-05 |
| uc022caj.1 | SDPR | 0.903549788 | 5.58E-05 |
| TCONS_00022516 | SDPR | 0.938862656 | 6.07E-06 |
| ENST00000607314.1 | NRG1 | 0.924893479 | 1.66E-05 |
| ENST00000439622.1 | NOL4L | 0.91710923 | 2.68E-05 |
| NR_027034.1 | NOL4L | 0.902378911 | 5.92E-05 |
| ENST00000548170.1 | NOL4L | -0.909628483 | 4.07E-05 |
| TCONS_00026223 | NOL4L | 0.915491043 | 2.94E-05 |
| ENST00000426704.1 | NOL4L | 0.904317867 | 5.37E-05 |
| ENST00000432967.1 | NOL4L | 0.91892233 | 2.41E-05 |
| ENST00000425104.1 | NOL4L | 0.933137903 | 9.40E-06 |
| ENST00000421937.3 | NOL4L | 0.910397577 | 3.91E-05 |
| TCONS_00012168 | NGFRAP1 | 0.924092001 | 1.75E-05 |
| ENST00000558120.1 | NGFRAP1 | 0.917409242 | 2.63E-05 |
| NR_027145.2 | NGFRAP1 | 0.968378498 | 2.36E-07 |
| ENST00000608442.1 | NGFRAP1 | 0.90134015 | 6.23E-05 |
| ENST00000554253.1 | NGFRAP1 | 0.900894286 | 6.36E-05 |
| ENST00000467896.2 | NGFRAP1 | 0.958371213 | 9.18E-07 |
| ENST00000420595.1 | NGFRAP1 | 0.937588726 | 6.71E-06 |
| TCONS_00018335 | NGFRAP1 | 0.9103853 | 3.91E-05 |
| ENST00000553317.1 | NGFRAP1 | 0.902293133 | 5.94E-05 |
| ENST00000451706.2 | NGFRAP1 | 0.902537299 | 5.87E-05 |
| LIT2094 | NGFRAP1 | 0.910412317 | 3.90E-05 |
| ENST00000513153.1 | NGFRAP1 | 0.933491152 | 9.16E-06 |
| ENST00000585784.1 | NGFRAP1 | 0.901427183 | 6.20E-05 |
| uc004ehp.2 | NGFRAP1 | 0.950462387 | 2.16E-06 |
| ENST00000430620.1 | NGFRAP1 | 0.911689852 | 3.64E-05 |
| ENST00000607175.1 | NGFRAP1 | 0.919104933 | 2.38E-05 |
| XR_427724.1 | NGFRAP1 | 0.905877843 | 4.96E-05 |
| uc022caj.1 | NGFRAP1 | 0.955590437 | 1.26E-06 |
| uc021thc.2 | A_33_P3768930 | 0.941543871 | 4.87E-06 |
| TCONS_00026436 | A_33_P3768930 | 0.910229418 | 3.94E-05 |
| XR_429426.1 | A_33_P3768930 | 0.923377992 | 1.83E-05 |
| XR_429429.1 | A_33_P3768930 | 0.968259522 | 2.41E-07 |
| uc002sti.1 | A_33_P3768930 | 0.973947125 | 9.05E-08 |
| ENST00000608505.1 | TBXA2R | 0.937346391 | 6.84E-06 |
| ENST00000558120.1 | TBXA2R | 0.901300648 | 6.24E-05 |
| ENST00000553317.1 | TBXA2R | 0.907444597 | 4.57E-05 |
| ENST00000602325.1 | TBXA2R | 0.910033353 | 3.98E-05 |
| LIT2094 | TBXA2R | 0.900100819 | 6.61E-05 |
| NR_037928.1 | TBXA2R | 0.947735355 | 2.81E-06 |
| ENST00000457661.1 | TBXA2R | 0.916504546 | 2.77E-05 |
| ENST00000424116.2 | TBXA2R | 0.958324612 | 9.23E-07 |
| ENST00000607175.1 | TBXA2R | 0.931819127 | 1.03E-05 |
| ENST00000525714.1 | PKIG | 0.913997637 | 3.20E-05 |
| RNA147249\|p0353_imsncRNA771 | PKIG | 0.941182624 | 5.02E-06 |
| XR_108564.1 | PKIG | 0.905798858 | 4.98E-05 |
| RNA147248\|p0352_imsncRNA771 | PKIG | 0.944738002 | 3.70E-06 |
| ENST00000530194.1 | PKIG | 0.924725178 | 1.68E-05 |
| ENST00000439823.1 | PKIG | 0.918075931 | 2.53E-05 |
| TCONS_00006817 | PKIG | -0.943094391 | 4.27E-06 |
| ENST00000555918.1 | MMD | 0.949655373 | 2.34E-06 |
| ENST00000558120.1 | MMD | 0.966853186 | 2.98E-07 |
| NR_027145.2 | MMD | 0.97231172 | 1.22E-07 |
| TCONS_00018467 | MMD | 0.919578976 | 2.31E-05 |
| XR_429366.1 | MMD | 0.914716913 | 3.07E-05 |
| ENST00000592441.1 | MMD | 0.914093183 | 3.19E-05 |
| XR_426860.1 | MMD | 0.949136274 | 2.46E-06 |
| ENST00000553317.1 | MMD | 0.944469007 | 3.79E-06 |
| NR_037928.1 | MMD | 0.901891199 | 6.06E-05 |
| ENST00000457661.1 | MMD | 0.915182369 | 2.99E-05 |
| ENST00000560924.1 | MMD | 0.933702883 | 9.02E-06 |
| ENST00000556936.1 | MMD | 0.924933446 | 1.65E-05 |
| ENST00000430620.1 | MMD | 0.921597276 | 2.04E-05 |
| ENST00000607175.1 | MMD | 0.910387644 | 3.91E-05 |
| ENST00000556996.1 | MMD | 0.933934349 | 8.87E-06 |
| TCONS_00022516 | MMD | 0.932032186 | 1.02E-05 |
| RNA147249\|p0353_imsncRNA771 | FCRL1 | 0.955822626 | 1.23E-06 |
| RNA147248\|p0352_imsncRNA771 | FCRL1 | 0.959487466 | 8.03E-07 |
| ENST00000530194.1 | FCRL1 | 0.962965698 | 5.16E-07 |
| ENST00000439823.1 | FCRL1 | 0.926905719 | 1.45E-05 |
| TCONS_00013375 | FCRL1 | 0.907370283 | 4.59E-05 |
| ENST00000458479.1 | OR5B21 | 0.93826247 | 6.36E-06 |
| ENST00000584683.1 | OR5B21 | 0.917690419 | 2.59E-05 |
| LIT1273 | OR5B21 | 0.941818517 | 4.76E-06 |
| ENST00000599026.1 | OR5B21 | 0.924409734 | 1.71E-05 |
| ENST00000416310.1 | OR5B21 | 0.923708653 | 1.79E-05 |
| ENST00000432411.1 | OR5B21 | 0.948588828 | 2.59E-06 |
| XR_429339.1 | OR5B21 | 0.940499172 | 5.31E-06 |
| HIT000078556_03 | A_33_P3250971 | 0.938610815 | 6.19E-06 |
| ENST00000430694.1 | A_33_P3250971 | 0.905315524 | 5.10E-05 |
| XR_429426.1 | A_33_P3250971 | 0.914307754 | 3.15E-05 |
| NR_026800.1 | A_33_P3250971 | 0.957511942 | 1.02E-06 |
| ENST00000558120.1 | TUBA8 | 0.946745629 | 3.08E-06 |
| XR_241556.1 | TUBA8 | 0.936617108 | 7.24E-06 |
| NR_027145.2 | TUBA8 | 0.952885561 | 1.69E-06 |
| ENST00000420595.1 | TUBA8 | 0.9430474 | 4.29E-06 |
| ENST00000442008.2 | TUBA8 | 0.934350296 | 8.60E-06 |
| ENST00000602325.1 | TUBA8 | 0.940390305 | 5.36E-06 |
| LIT2094 | TUBA8 | 0.956885322 | 1.09E-06 |
| NR_037928.1 | TUBA8 | 0.948457286 | 2.63E-06 |
| uc004ehp.2 | TUBA8 | 0.924359208 | 1.72E-05 |
| ENST00000424116.2 | TUBA8 | 0.966945442 | 2.94E-07 |
| ENST00000607175.1 | TUBA8 | 0.934281623 | 8.64E-06 |
| uc022caj.1 | TUBA8 | 0.916693603 | 2.74E-05 |
| TCONS_00022516 | TUBA8 | 0.932323145 | 9.97E-06 |
| ENST00000558120.1 | CLU | 0.935421428 | 7.93E-06 |
| NR_027145.2 | CLU | 0.903296377 | 5.65E-05 |
| ENST00000561134.1 | CLU | 0.90303894 | 5.72E-05 |
| TCONS_00018467 | CLU | 0.907548075 | 4.55E-05 |
| ENST00000442008.2 | CLU | 0.918407187 | 2.48E-05 |
| NR_037928.1 | CLU | 0.921436338 | 2.06E-05 |
| TCONS_00018641 | CLU | 0.902579115 | 5.86E-05 |
| uc004ehp.2 | CLU | 0.902162882 | 5.98E-05 |
| ENST00000424116.2 | CLU | 0.919472623 | 2.33E-05 |
| uc022caj.1 | CLU | 0.903281168 | 5.66E-05 |
| TCONS_00022516 | CLU | 0.911922725 | 3.60E-05 |
| LIT2094 | GPX1 | 0.935484105 | 7.89E-06 |
| NR_037928.1 | GPX1 | 0.952129163 | 1.83E-06 |
| ENST00000424116.2 | GPX1 | 0.900565171 | 6.47E-05 |
| TCONS_00024392 | GPX1 | -0.912951999 | 3.40E-05 |
| ENST00000558120.1 | C2orf88 | 0.912570587 | 3.47E-05 |
| NR_027145.2 | C2orf88 | 0.923141738 | 1.85E-05 |
| XR_429366.1 | C2orf88 | 0.906649816 | 4.76E-05 |
| XR_426860.1 | C2orf88 | 0.936043371 | 7.57E-06 |
| uc022caj.1 | C2orf88 | 0.905268902 | 5.12E-05 |
| TCONS_00022516 | C2orf88 | 0.931490273 | 1.06E-05 |
| TCONS_00026436 | IGJ | 0.913688408 | 3.26E-05 |
| XR_429429.1 | IGJ | 0.9147977 | 3.06E-05 |
| uc002sti.1 | IGJ | 0.965966212 | 3.40E-07 |
| ENST00000608505.1 | RNF208 | 0.917472184 | 2.62E-05 |
| ENST00000608442.1 | RNF208 | 0.940378587 | 5.37E-06 |
| ENST00000445429.1 | RNF208 | 0.904876815 | 5.22E-05 |
| ENST00000420595.1 | RNF208 | 0.900680313 | 6.43E-05 |
| ENST00000602325.1 | RNF208 | 0.915170899 | 3.00E-05 |
| LIT2094 | RNF208 | 0.913199932 | 3.35E-05 |
| ENST00000457661.1 | RNF208 | 0.928404985 | 1.31E-05 |
| ENST00000430620.1 | RNF208 | 0.903721543 | 5.53E-05 |
| ENST00000424116.2 | RNF208 | 0.936954146 | 7.05E-06 |
| ENST00000607175.1 | RNF208 | 0.936806702 | 7.13E-06 |
| HIT000078556_03 | A_33_P3364959 | 0.928829525 | 1.28E-05 |
| uc021thc.2 | A_33_P3364959 | 0.955949905 | 1.21E-06 |
| XR_429426.1 | A_33_P3364959 | 0.931479923 | 1.06E-05 |
| XR_429429.1 | A_33_P3364959 | 0.973962399 | 9.02E-08 |
| uc002sti.1 | A_33_P3364959 | 0.9614773 | 6.26E-07 |
| ENST00000561134.1 | MYL9 | 0.960889058 | 6.75E-07 |
| ENST00000558120.1 | TUBB2A | 0.926537354 | 1.49E-05 |
| NR_027145.2 | TUBB2A | 0.933396994 | 9.22E-06 |
| ENST00000554253.1 | TUBB2A | 0.92406409 | 1.75E-05 |
| ENST00000420595.1 | TUBB2A | 0.938032771 | 6.48E-06 |
| TCONS_00018335 | TUBB2A | 0.913210232 | 3.35E-05 |
| ENST00000442008.2 | TUBB2A | 0.944658932 | 3.72E-06 |
| ENST00000553317.1 | TUBB2A | 0.906962598 | 4.69E-05 |
| ENST00000602325.1 | TUBB2A | 0.941687838 | 4.81E-06 |
| ENST00000451706.2 | TUBB2A | 0.923487167 | 1.81E-05 |
| LIT2094 | TUBB2A | 0.950184907 | 2.22E-06 |
| NR_037928.1 | TUBB2A | 0.918343198 | 2.49E-05 |
| ENST00000457661.1 | TUBB2A | 0.907994171 | 4.44E-05 |
| ENST00000513153.1 | TUBB2A | 0.906313183 | 4.85E-05 |
| uc004ehp.2 | TUBB2A | 0.922829093 | 1.89E-05 |
| ENST00000424116.2 | TUBB2A | 0.923400499 | 1.82E-05 |
| ENST00000607175.1 | TUBB2A | 0.90748586 | 4.56E-05 |
| uc022caj.1 | TUBB2A | 0.946485937 | 3.16E-06 |
| TCONS_00022516 | TUBB2A | 0.933627659 | 9.07E-06 |
| TCONS_00012168 | ARHGAP6 | 0.909358514 | 4.13E-05 |
| ENST00000558120.1 | ARHGAP6 | 0.908051566 | 4.43E-05 |
| LIT2061 | ARHGAP6 | 0.914145159 | 3.18E-05 |
| NR_027145.2 | ARHGAP6 | 0.936247314 | 7.45E-06 |
| ENST00000420595.1 | ARHGAP6 | 0.947856814 | 2.78E-06 |
| XR_426860.1 | ARHGAP6 | 0.909621408 | 4.07E-05 |
| uc022caj.1 | ARHGAP6 | 0.908226047 | 4.39E-05 |
| TCONS_00022516 | ARHGAP6 | 0.953930582 | 1.51E-06 |
| HIT000078556_03 | A_33_P3331228 | 0.964936731 | 3.94E-07 |
| uc021thc.2 | A_33_P3331228 | 0.914421054 | 3.13E-05 |
| XR_429426.1 | A_33_P3331228 | 0.93623715 | 7.45E-06 |
| NR_026800.1 | A_33_P3331228 | 0.91328445 | 3.33E-05 |
| XR_429429.1 | A_33_P3331228 | 0.934061281 | 8.78E-06 |
| ENST00000510536.1 | PBX1 | 0.912847636 | 3.42E-05 |
| ENST00000450238.1 | PBX1 | 0.929553366 | 1.21E-05 |
| ENST00000454015.1 | PBX1 | 0.91837771 | 2.48E-05 |
| TCONS_00012168 | RNF208 | 0.918291691 | 2.50E-05 |
| ENST00000608505.1 | RNF208 | 0.924982011 | 1.65E-05 |
| ENST00000558120.1 | RNF208 | 0.904950987 | 5.20E-05 |
| LIT2061 | RNF208 | 0.916198323 | 2.82E-05 |
| NR_027145.2 | RNF208 | 0.936144717 | 7.51E-06 |
| ENST00000608442.1 | RNF208 | 0.957558974 | 1.01E-06 |
| ENST00000554253.1 | RNF208 | 0.928279712 | 1.32E-05 |
| ENST00000467896.2 | RNF208 | 0.920939062 | 2.13E-05 |
| ENST00000420595.1 | RNF208 | 0.919337908 | 2.35E-05 |
| ENST00000442008.2 | RNF208 | 0.910887418 | 3.80E-05 |
| ENST00000553317.1 | RNF208 | 0.923699372 | 1.79E-05 |
| ENST00000602325.1 | RNF208 | 0.923193892 | 1.85E-05 |
| LIT2094 | RNF208 | 0.935600033 | 7.83E-06 |
| NR_037928.1 | RNF208 | 0.903899414 | 5.48E-05 |
| ENST00000457661.1 | RNF208 | 0.942697492 | 4.42E-06 |
| NR_037616.1 | RNF208 | 0.910343983 | 3.92E-05 |
| ENST00000430620.1 | RNF208 | 0.917595584 | 2.60E-05 |
| ENST00000424116.2 | RNF208 | 0.942912949 | 4.34E-06 |
| ENST00000607175.1 | RNF208 | 0.964413111 | 4.23E-07 |
| XR_427724.1 | RNF208 | 0.908343109 | 4.36E-05 |
| ENST00000555918.1 | MTURN | 0.920923555 | 2.13E-05 |
| ENST00000608505.1 | MTURN | 0.904620308 | 5.29E-05 |
| ENST00000558120.1 | MTURN | 0.945031836 | 3.60E-06 |
| XR_241556.1 | MTURN | 0.923868952 | 1.77E-05 |
| NR_027145.2 | MTURN | 0.940486714 | 5.32E-06 |
| ENST00000561134.1 | MTURN | 0.945976444 | 3.31E-06 |
| ENST00000420595.1 | MTURN | 0.919058799 | 2.39E-05 |
| ENST00000442008.2 | MTURN | 0.927559234 | 1.39E-05 |
| ENST00000553317.1 | MTURN | 0.91436716 | 3.14E-05 |
| ENST00000602325.1 | MTURN | 0.938987235 | 6.01E-06 |
| LIT2094 | MTURN | 0.916175644 | 2.83E-05 |
| NR_037928.1 | MTURN | 0.955859336 | 1.23E-06 |
| ENST00000424116.2 | MTURN | 0.966763133 | 3.02E-07 |
| ENST00000607175.1 | MTURN | 0.957331756 | 1.04E-06 |
| TCONS_00022516 | MTURN | 0.90054469 | 6.47E-05 |
| ENST00000555918.1 | MSN | 0.911397193 | 3.70E-05 |
| NR_027145.2 | MSN | 0.920129655 | 2.24E-05 |
| ENST00000447323.1 | MSN | 0.953729275 | 1.55E-06 |
| ENST00000554253.1 | MSN | 0.917684151 | 2.59E-05 |
| ENST00000442008.2 | MSN | 0.91124055 | 3.73E-05 |
| ENST00000553317.1 | MSN | 0.929749295 | 1.20E-05 |
| NR_037928.1 | MSN | 0.90417953 | 5.41E-05 |
| ENST00000457661.1 | MSN | 0.900397747 | 6.52E-05 |
| ENST00000607175.1 | MSN | 0.910875399 | 3.81E-05 |
| uc022caj.1 | MSN | 0.911331525 | 3.71E-05 |
| ENST00000535511.1 | NAT8B | 0.916477667 | 2.78E-05 |
| NR_027034.1 | HGD | -0.923154775 | 1.85E-05 |
| ENST00000467896.2 | HGD | 0.90364559 | 5.55E-05 |
| HIT000078556_03 | A_24_P212024 | 0.990116262 | 7.31E-10 |
| uc021thc.2 | A_24_P212024 | 0.943713336 | 4.05E-06 |
| XR_429426.1 | A_24_P212024 | 0.92297154 | 1.87E-05 |
| XR_429429.1 | A_24_P212024 | 0.942549842 | 4.47E-06 |
| ENST00000432967.1 | TRAPPC10 | 0.926750609 | 1.47E-05 |
| ENST00000518090.1 | MPP1 | 0.927669613 | 1.38E-05 |
| ENST00000608505.1 | MPP1 | 0.917775891 | 2.58E-05 |
| ENST00000558120.1 | MPP1 | 0.930557139 | 1.13E-05 |
| XR_241556.1 | MPP1 | 0.950664894 | 2.12E-06 |
| NR_027145.2 | MPP1 | 0.912004264 | 3.58E-05 |
| ENST00000602325.1 | MPP1 | 0.926003044 | 1.54E-05 |
| LIT2094 | MPP1 | 0.912162853 | 3.55E-05 |
| NR_037928.1 | MPP1 | 0.950467934 | 2.16E-06 |
| ENST00000457661.1 | MPP1 | 0.919767091 | 2.29E-05 |
| ENST00000508021.1 | MPP1 | 0.904160341 | 5.41E-05 |
| ENST00000560924.1 | MPP1 | 0.907646943 | 4.52E-05 |
| ENST00000424116.2 | MPP1 | 0.945715324 | 3.39E-06 |
| TCONS_00022516 | MPP1 | 0.924995426 | 1.65E-05 |
| HIT000078556_03 | A_24_P101226 | 0.954353628 | 1.45E-06 |
| HIT000078556_03 | A_33_P3424612 | 0.904769714 | 5.25E-05 |
| uc021thc.2 | A_33_P3424612 | 0.949142407 | 2.46E-06 |
| TCONS_00026436 | A_33_P3424612 | 0.94867796 | 2.57E-06 |
| XR_429426.1 | A_33_P3424612 | 0.959946697 | 7.59E-07 |
| XR_429429.1 | A_33_P3424612 | 0.976437783 | 5.50E-08 |
| uc002sti.1 | A_33_P3424612 | 0.974932328 | 7.47E-08 |
| TCONS_00012168 | RAB27B | 0.920398715 | 2.20E-05 |
| ENST00000558120.1 | RAB27B | 0.922538042 | 1.93E-05 |
| LIT2061 | RAB27B | 0.938018372 | 6.49E-06 |
| NR_027145.2 | RAB27B | 0.950017012 | 2.26E-06 |
| ENST00000467896.2 | RAB27B | 0.932985384 | 9.51E-06 |
| ENST00000420595.1 | RAB27B | 0.913554359 | 3.28E-05 |
| ENST00000442008.2 | RAB27B | 0.910098511 | 3.97E-05 |
| ENST00000513153.1 | RAB27B | 0.905852742 | 4.96E-05 |
| ENST00000450238.1 | RAB27B | 0.911296578 | 3.72E-05 |
| uc004ehp.2 | RAB27B | 0.9159118 | 2.87E-05 |
| TCONS_00010294 | RAB27B | 0.904735358 | 5.26E-05 |
| ENST00000424116.2 | RAB27B | 0.902426558 | 5.90E-05 |
| ENST00000607175.1 | RAB27B | 0.923469765 | 1.82E-05 |
| uc022caj.1 | RAB27B | 0.930446841 | 1.14E-05 |
| TCONS_00022516 | RAB27B | 0.922551894 | 1.92E-05 |
| NR_022010.1 | ATF3 | -0.921814951 | 2.02E-05 |
| TCONS_00018268 | ATF3 | -0.919574568 | 2.31E-05 |
| LIT1273 | ATF3 | -0.913944226 | 3.21E-05 |
| TCONS_00005965 | ATF3 | 0.903464884 | 5.60E-05 |
| ENST00000599026.1 | ATF3 | -0.938002086 | 6.50E-06 |
| ENST00000562983.1 | ATF3 | -0.911405024 | 3.70E-05 |
| uc002tzb.1 | ATF3 | -0.918109845 | 2.52E-05 |
| ENST00000551146.1 | ATF3 | -0.928168938 | 1.33E-05 |
| ENST00000555918.1 | GRAP2 | 0.915277911 | 2.98E-05 |
| ENST00000558120.1 | GRAP2 | 0.940511829 | 5.31E-06 |
| NR_027145.2 | GRAP2 | 0.942532122 | 4.48E-06 |
| ENST00000608442.1 | GRAP2 | 0.920072434 | 2.24E-05 |
| ENST00000554253.1 | GRAP2 | 0.958823015 | 8.70E-07 |
| ENST00000420595.1 | GRAP2 | 0.92662649 | 1.48E-05 |
| TCONS_00018335 | GRAP2 | 0.904790528 | 5.24E-05 |
| ENST00000442008.2 | GRAP2 | 0.960828265 | 6.80E-07 |
| ENST00000553317.1 | GRAP2 | 0.947428991 | 2.89E-06 |
| ENST00000602325.1 | GRAP2 | 0.943401979 | 4.16E-06 |
| ENST00000451706.2 | GRAP2 | 0.908253074 | 4.38E-05 |
| LIT2094 | GRAP2 | 0.948544009 | 2.61E-06 |
| NR_037928.1 | GRAP2 | 0.939565108 | 5.73E-06 |
| ENST00000457661.1 | GRAP2 | 0.921434296 | 2.06E-05 |
| ENST00000506718.1 | GRAP2 | 0.901180452 | 6.27E-05 |
| ENST00000424116.2 | GRAP2 | 0.944375108 | 3.82E-06 |
| ENST00000607175.1 | GRAP2 | 0.957362869 | 1.03E-06 |
| XR_427724.1 | GRAP2 | 0.904276827 | 5.38E-05 |
| uc022caj.1 | GRAP2 | 0.917973476 | 2.55E-05 |
| TCONS_00022516 | GRAP2 | 0.911228483 | 3.73E-05 |
| RNA147577\|p0681_imsncRNA442 | LOC100128670 | 0.953818737 | 1.53E-06 |
| ENST00000522740.1 | LOC100128670 | 0.909465479 | 4.11E-05 |
| ENST00000432411.1 | LOC100128670 | 0.927064582 | 1.44E-05 |
| HIT000078556_03 | DENND5B | 0.903425415 | 5.61E-05 |
| XR_430238.1 | DENND5B | 0.901597831 | 6.15E-05 |
| ENST00000534089.1 | ZNF768 | -0.90426722 | 5.38E-05 |
| ENST00000555918.1 | F13A1 | 0.938498823 | 6.25E-06 |
| ENST00000608505.1 | F13A1 | 0.919522132 | 2.32E-05 |
| ENST00000558120.1 | F13A1 | 0.978369138 | 3.60E-08 |
| XR_241556.1 | F13A1 | 0.930217074 | 1.16E-05 |
| NR_027145.2 | F13A1 | 0.950072566 | 2.25E-06 |
| ENST00000561134.1 | F13A1 | 0.91197374 | 3.59E-05 |
| ENST00000554253.1 | F13A1 | 0.925251255 | 1.62E-05 |
| ENST00000420595.1 | F13A1 | 0.90480784 | 5.24E-05 |
| ENST00000442008.2 | F13A1 | 0.930624523 | 1.13E-05 |
| ENST00000553317.1 | F13A1 | 0.948142519 | 2.71E-06 |
| ENST00000602325.1 | F13A1 | 0.936563752 | 7.27E-06 |
| LIT2094 | F13A1 | 0.921982095 | 1.99E-05 |
| NR_037928.1 | F13A1 | 0.969183191 | 2.08E-07 |
| ENST00000457661.1 | F13A1 | 0.907741781 | 4.50E-05 |
| ENST00000560924.1 | F13A1 | 0.908121481 | 4.41E-05 |
| ENST00000506718.1 | F13A1 | 0.939073743 | 5.97E-06 |
| ENST00000424116.2 | F13A1 | 0.964392526 | 4.25E-07 |
| ENST00000607175.1 | F13A1 | 0.958144769 | 9.43E-07 |
| TCONS_00022516 | F13A1 | 0.93503396 | 8.17E-06 |
| ENST00000608505.1 | HIST1H2AC | 0.936319809 | 7.41E-06 |
| NR_027145.2 | HIST1H2AC | 0.90089057 | 6.36E-05 |
| XR_430341.1 | HIST1H2AC | 0.923625755 | 1.80E-05 |
| ENST00000602325.1 | HIST1H2AC | 0.908436857 | 4.34E-05 |
| LIT2094 | HIST1H2AC | 0.918936118 | 2.40E-05 |
| NR_037928.1 | HIST1H2AC | 0.912192344 | 3.54E-05 |
| ENST00000457661.1 | HIST1H2AC | 0.929431866 | 1.22E-05 |
| ENST00000424116.2 | HIST1H2AC | 0.94040876 | 5.35E-06 |
| ENST00000607175.1 | HIST1H2AC | 0.915326654 | 2.97E-05 |
| ENST00000555918.1 | SMOX | 0.912131293 | 3.55E-05 |
| ENST00000608505.1 | SMOX | 0.904071561 | 5.44E-05 |
| ENST00000558120.1 | SMOX | 0.952103172 | 1.83E-06 |
| LIT2061 | SMOX | 0.902615815 | 5.85E-05 |
| NR_027145.2 | SMOX | 0.946644085 | 3.11E-06 |
| ENST00000554253.1 | SMOX | 0.908994107 | 4.21E-05 |
| ENST00000467896.2 | SMOX | 0.912447674 | 3.49E-05 |
| ENST00000592441.1 | SMOX | 0.907373278 | 4.59E-05 |
| ENST00000442008.2 | SMOX | 0.904410954 | 5.34E-05 |
| ENST00000553317.1 | SMOX | 0.959528965 | 7.99E-07 |
| NR_037928.1 | SMOX | 0.904358548 | 5.36E-05 |
| ENST00000457661.1 | SMOX | 0.905056674 | 5.17E-05 |
| NR_037616.1 | SMOX | 0.936027286 | 7.57E-06 |
| ENST00000556936.1 | SMOX | 0.917189023 | 2.67E-05 |
| ENST00000424116.2 | SMOX | 0.937176757 | 6.93E-06 |
| ENST00000607175.1 | SMOX | 0.966707417 | 3.05E-07 |
| ENST00000556996.1 | SMOX | 0.904836581 | 5.23E-05 |
| TCONS_00022516 | SMOX | 0.912363483 | 3.51E-05 |
| HIT000078556_03 | PRG2 | 0.926543665 | 1.49E-05 |
| uc021thc.2 | PRG2 | 0.950373608 | 2.18E-06 |
| TCONS_00026436 | PRG2 | 0.935731266 | 7.75E-06 |
| XR_429426.1 | PRG2 | 0.964875382 | 3.97E-07 |
| XR_429429.1 | PRG2 | 0.981195288 | 1.79E-08 |
| uc002sti.1 | PRG2 | 0.972973839 | 1.09E-07 |
| NR_027145.2 | ARHGAP6 | 0.933889261 | 8.90E-06 |
| ENST00000510536.1 | ARHGAP6 | 0.931454747 | 1.06E-05 |
| ENST00000420595.1 | ARHGAP6 | 0.932864279 | 9.59E-06 |
| XR_426860.1 | ARHGAP6 | 0.920761931 | 2.15E-05 |
| ENST00000451706.2 | ARHGAP6 | 0.921321351 | 2.08E-05 |
| XR_427724.1 | ARHGAP6 | 0.902604759 | 5.85E-05 |
| ENST00000530194.1 | RASGRP3 | 0.937987158 | 6.50E-06 |
| TCONS_00008872 | RASGRP3 | 0.911456254 | 3.69E-05 |
| TCONS_00013375 | RASGRP3 | 0.914629492 | 3.09E-05 |
| TCONS_00013375 | BLNK | 0.923248302 | 1.84E-05 |
| TCONS_00017180 | BLNK | 0.942579108 | 4.46E-06 |
| ENST00000558120.1 | RGS10 | 0.915689485 | 2.91E-05 |
| NR_027145.2 | RGS10 | 0.939418304 | 5.80E-06 |
| ENST00000608442.1 | RGS10 | 0.902447198 | 5.90E-05 |
| ENST00000554253.1 | RGS10 | 0.915064777 | 3.01E-05 |
| ENST00000467896.2 | RGS10 | 0.936346296 | 7.39E-06 |
| ENST00000420595.1 | RGS10 | 0.923521935 | 1.81E-05 |
| ENST00000442008.2 | RGS10 | 0.929513763 | 1.22E-05 |
| ENST00000553317.1 | RGS10 | 0.906666505 | 4.76E-05 |
| ENST00000602325.1 | RGS10 | 0.901586064 | 6.15E-05 |
| ENST00000451706.2 | RGS10 | 0.917452875 | 2.62E-05 |
| LIT2094 | RGS10 | 0.940556834 | 5.29E-06 |
| NR_037928.1 | RGS10 | 0.907658063 | 4.52E-05 |
| ENST00000457661.1 | RGS10 | 0.908503428 | 4.32E-05 |
| ENST00000513153.1 | RGS10 | 0.928834037 | 1.27E-05 |
| NR_037616.1 | RGS10 | 0.914844967 | 3.05E-05 |
| uc004ehp.2 | RGS10 | 0.931384502 | 1.07E-05 |
| ENST00000430620.1 | RGS10 | 0.914597168 | 3.10E-05 |
| ENST00000424116.2 | RGS10 | 0.926976842 | 1.45E-05 |
| ENST00000607175.1 | RGS10 | 0.926181987 | 1.52E-05 |
| uc022caj.1 | RGS10 | 0.946633903 | 3.12E-06 |
| ENST00000518090.1 | PCYT1B | 0.900100141 | 6.61E-05 |
| ENST00000558120.1 | PCYT1B | 0.906962529 | 4.69E-05 |
| XR_241556.1 | PCYT1B | 0.900659315 | 6.44E-05 |
| NR_027145.2 | PCYT1B | 0.951747213 | 1.90E-06 |
| TCONS_00018467 | PCYT1B | 0.914247327 | 3.16E-05 |
| ENST00000420595.1 | PCYT1B | 0.921335556 | 2.08E-05 |
| ENST00000592441.1 | PCYT1B | 0.916693725 | 2.74E-05 |
| ENST00000424116.2 | PCYT1B | 0.906963799 | 4.69E-05 |
| ENST00000607175.1 | PCYT1B | 0.92939936 | 1.23E-05 |
| ENST00000518090.1 | A_33_P3327192 | 0.920728834 | 2.16E-05 |
| ENST00000608505.1 | A_33_P3327192 | 0.952483927 | 1.76E-06 |
| ENST00000558120.1 | A_33_P3327192 | 0.950845336 | 2.08E-06 |
| NR_027145.2 | A_33_P3327192 | 0.917671259 | 2.59E-05 |
| ENST00000554253.1 | A_33_P3327192 | 0.921864014 | 2.01E-05 |
| XR_426860.1 | A_33_P3327192 | 0.900262295 | 6.56E-05 |
| ENST00000553317.1 | A_33_P3327192 | 0.958353682 | 9.20E-07 |
| ENST00000602325.1 | A_33_P3327192 | 0.906677766 | 4.76E-05 |
| NR_037928.1 | A_33_P3327192 | 0.94106247 | 5.07E-06 |
| ENST00000457661.1 | A_33_P3327192 | 0.962507225 | 5.48E-07 |
| ENST00000560924.1 | A_33_P3327192 | 0.904373487 | 5.35E-05 |
| ENST00000556936.1 | A_33_P3327192 | 0.949680865 | 2.33E-06 |
| ENST00000424116.2 | A_33_P3327192 | 0.938892124 | 6.05E-06 |
| ENST00000607175.1 | A_33_P3327192 | 0.928910872 | 1.27E-05 |
| ENST00000556996.1 | A_33_P3327192 | 0.905620206 | 5.02E-05 |
| TCONS_00022516 | A_33_P3327192 | 0.933895096 | 8.89E-06 |
| XR_241556.1 | NCKAP1 | 0.909470004 | 4.11E-05 |
| ENST00000602325.1 | NCKAP1 | 0.920039856 | 2.25E-05 |
| LIT2094 | NCKAP1 | 0.903542695 | 5.58E-05 |
| TCONS_00026411 | DAPP1 | 0.90012245 | 6.61E-05 |
| ENST00000580291.1 | DAPP1 | -0.921133631 | 2.10E-05 |
| ENST00000445429.1 | DAPP1 | 0.933021892 | 9.48E-06 |
| ENST00000457661.1 | DAPP1 | 0.923470967 | 1.82E-05 |
| XR_427746.1 | DAPP1 | 0.903926996 | 5.48E-05 |
| ENST00000430620.1 | DAPP1 | 0.92256271 | 1.92E-05 |
| XR_427724.1 | DAPP1 | 0.90930483 | 4.14E-05 |
| RNA147248\|p0352_imsncRNA771 | PCDH9 | 0.903911162 | 5.48E-05 |
| eHIT000015952 | UGT3A1 | 0.905215905 | 5.13E-05 |
| TCONS_00024308 | UGT3A1 | -0.909329328 | 4.14E-05 |
| HIT000078556_03 | A_24_P15388 | 0.960938195 | 6.71E-07 |
| uc021thc.2 | A_24_P15388 | 0.915929864 | 2.87E-05 |
| XR_429426.1 | A_24_P15388 | 0.957361606 | 1.03E-06 |
| NR_026800.1 | A_24_P15388 | 0.934011498 | 8.81E-06 |
| XR_429429.1 | A_24_P15388 | 0.936770324 | 7.15E-06 |
| ENST00000441316.1 | MCUR1 | 0.909633767 | 4.07E-05 |
| LIT2061 | GSTA5 | 0.90652888 | 4.79E-05 |
| ENST00000510536.1 | GSTA5 | 0.907906076 | 4.46E-05 |
| ENST00000608505.1 | MPL | 0.919317472 | 2.35E-05 |
| ENST00000447298.1 | MPL | -0.918841218 | 2.42E-05 |
| ENST00000424116.2 | MPL | 0.925195315 | 1.63E-05 |
| ENST00000607175.1 | MPL | 0.945478493 | 3.46E-06 |
| XR_241556.1 | VWF | 0.91186546 | 3.61E-05 |
| TCONS_00024392 | VWF | -0.934233 | 8.67E-06 |
| TCONS_00025508 | HIST1H2AI | -0.947710558 | 2.82E-06 |
| NR_027145.2 | FHL1 | 0.921216115 | 2.09E-05 |
| ENST00000450238.1 | FHL1 | 0.900812142 | 6.39E-05 |
| ENST00000503611.1 | CCDC149 | 0.92534254 | 1.61E-05 |
| RNA147249\|p0353_imsncRNA771 | PPAPDC1B | 0.967641616 | 2.65E-07 |
| RNA147248\|p0352_imsncRNA771 | PPAPDC1B | 0.970912779 | 1.56E-07 |
| ENST00000530194.1 | PPAPDC1B | 0.904735863 | 5.26E-05 |
| TCONS_00006817 | PPAPDC1B | -0.908178436 | 4.40E-05 |
| ENST00000602954.1 | OR6N1 | -0.900563841 | 6.47E-05 |
| HIT000078556_03 | A_24_P315862 | 0.943592453 | 4.09E-06 |
| uc021thc.2 | A_24_P315862 | 0.95455458 | 1.41E-06 |
| XR_429426.1 | A_24_P315862 | 0.929467559 | 1.22E-05 |
| NR_026800.1 | A_24_P315862 | 0.904586768 | 5.30E-05 |
| XR_429429.1 | A_24_P315862 | 0.945337052 | 3.51E-06 |
| HIT000078556_03 | A_33_P3331150 | 0.933242944 | 9.33E-06 |
| XR_429426.1 | A_33_P3331150 | 0.910039873 | 3.98E-05 |
| ENST00000558120.1 | MGLL | 0.905506323 | 5.05E-05 |
| XR_241556.1 | MGLL | 0.952051201 | 1.84E-06 |
| ENST00000561134.1 | MGLL | 0.908407039 | 4.35E-05 |
| NR_037928.1 | MGLL | 0.947152333 | 2.97E-06 |
| ENST00000424116.2 | MGLL | 0.93075439 | 1.12E-05 |
| ENST00000506665.1 | PTGER3 | 0.901716905 | 6.11E-05 |
| ENST00000419027.1 | PTGER3 | -0.920702691 | 2.16E-05 |
| ENST00000555918.1 | F13A1 | 0.936054324 | 7.56E-06 |
| ENST00000608505.1 | F13A1 | 0.90485292 | 5.22E-05 |
| ENST00000558120.1 | F13A1 | 0.960152213 | 7.40E-07 |
| XR_241556.1 | F13A1 | 0.902623254 | 5.84E-05 |
| NR_027145.2 | F13A1 | 0.921407741 | 2.07E-05 |
| ENST00000554253.1 | F13A1 | 0.935650541 | 7.80E-06 |
| ENST00000442008.2 | F13A1 | 0.931472942 | 1.06E-05 |
| ENST00000553317.1 | F13A1 | 0.945888015 | 3.34E-06 |
| ENST00000602325.1 | F13A1 | 0.917076097 | 2.68E-05 |
| LIT2094 | F13A1 | 0.914386382 | 3.13E-05 |
| NR_037928.1 | F13A1 | 0.960560442 | 7.03E-07 |
| ENST00000506718.1 | F13A1 | 0.963752564 | 4.64E-07 |
| ENST00000424116.2 | F13A1 | 0.940160072 | 5.46E-06 |
| ENST00000607175.1 | F13A1 | 0.941308678 | 4.97E-06 |
| TCONS_00022516 | F13A1 | 0.907379382 | 4.59E-05 |
| HIT000078556_03 | A_33_P3235876 | 0.96492413 | 3.94E-07 |
| uc021thc.2 | A_33_P3235876 | 0.914769356 | 3.07E-05 |
| XR_429426.1 | A_33_P3235876 | 0.933384818 | 9.23E-06 |
| XR_429429.1 | A_33_P3235876 | 0.931918379 | 1.03E-05 |
| ENST00000558120.1 | GMPR | 0.937527381 | 6.74E-06 |
| NR_027145.2 | GMPR | 0.950128005 | 2.23E-06 |
| ENST00000447323.1 | GMPR | 0.93582804 | 7.69E-06 |
| TCONS_00018467 | GMPR | 0.901134526 | 6.29E-05 |
| ENST00000553317.1 | GMPR | 0.908783624 | 4.26E-05 |
| ENST00000602325.1 | GMPR | 0.909980318 | 4.00E-05 |
| NR_037928.1 | GMPR | 0.935378119 | 7.96E-06 |
| ENST00000457661.1 | GMPR | 0.906776202 | 4.73E-05 |
| ENST00000424116.2 | GMPR | 0.920756122 | 2.15E-05 |
| ENST00000607175.1 | GMPR | 0.902179939 | 5.97E-05 |
| ENST00000608995.1 | INTU | -0.90653584 | 4.79E-05 |
| ENST00000602918.1 | INTU | 0.902521893 | 5.87E-05 |
| HIT000078556_03 | A_23_P361654 | 0.973115258 | 1.06E-07 |
| uc021thc.2 | A_23_P361654 | 0.955953432 | 1.21E-06 |
| XR_429426.1 | A_23_P361654 | 0.961111022 | 6.56E-07 |
| XR_429429.1 | A_23_P361654 | 0.971188343 | 1.49E-07 |
| uc002sti.1 | A_23_P361654 | 0.934527242 | 8.48E-06 |
| HIT000078556_03 | A_32_P157927 | 0.933361411 | 9.25E-06 |
| uc021thc.2 | A_32_P157927 | 0.943262702 | 4.21E-06 |
| XR_429426.1 | A_32_P157927 | 0.943112476 | 4.26E-06 |
| XR_429429.1 | A_32_P157927 | 0.958115531 | 9.46E-07 |
| uc002sti.1 | A_32_P157927 | 0.935433595 | 7.92E-06 |
| uc009wka.2 | TAS2R41 | 0.960939399 | 6.71E-07 |
| TCONS_00000305 | TAS2R41 | 0.921263807 | 2.09E-05 |
| ENST00000555918.1 | TDRP | 0.911592987 | 3.66E-05 |
| ENST00000608505.1 | TDRP | 0.933685427 | 9.03E-06 |
| ENST00000558120.1 | TDRP | 0.954224531 | 1.47E-06 |
| NR_027145.2 | TDRP | 0.932278697 | 1.00E-05 |
| ENST00000554253.1 | TDRP | 0.937157323 | 6.94E-06 |
| ENST00000442008.2 | TDRP | 0.930631068 | 1.13E-05 |
| ENST00000553317.1 | TDRP | 0.964000927 | 4.48E-07 |
| ENST00000602325.1 | TDRP | 0.913209449 | 3.35E-05 |
| LIT2094 | TDRP | 0.903194219 | 5.68E-05 |
| NR_037928.1 | TDRP | 0.947191697 | 2.96E-06 |
| ENST00000457661.1 | TDRP | 0.958654295 | 8.88E-07 |
| NR_037616.1 | TDRP | 0.92455582 | 1.69E-05 |
| ENST00000556936.1 | TDRP | 0.929910889 | 1.18E-05 |
| ENST00000430620.1 | TDRP | 0.912643736 | 3.45E-05 |
| ENST00000424116.2 | TDRP | 0.947157686 | 2.97E-06 |
| ENST00000607175.1 | TDRP | 0.935670099 | 7.78E-06 |
| TCONS_00022516 | TDRP | 0.932285966 | 1.00E-05 |
| HIT000078556_03 | A_32_P65589 | 0.952578181 | 1.74E-06 |
| uc021thc.2 | A_32_P65589 | 0.921052317 | 2.11E-05 |
| XR_429426.1 | A_32_P65589 | 0.938215145 | 6.39E-06 |
| NR_026800.1 | A_32_P65589 | 0.958153103 | 9.42E-07 |
| XR_429429.1 | A_32_P65589 | 0.920552548 | 2.18E-05 |
| ENST00000454861.1 | lnc-PPIAL4G-4 | 0.919972408 | 2.26E-05 |
| ENST00000412092.1 | lnc-PPIAL4G-4 | 0.982260676 | 1.34E-08 |
| ENST00000419027.1 | lnc-PPIAL4G-4 | 0.9976246 | 5.93E-13 |
| HIT000078556_03 | A_24_P24053 | 0.951875329 | 1.87E-06 |
| XR_429426.1 | A_24_P24053 | 0.915434272 | 2.95E-05 |
| NR_026800.1 | A_24_P24053 | 0.917031641 | 2.69E-05 |
| XR_429429.1 | A_24_P24053 | 0.916336411 | 2.80E-05 |
| HIT000078556_03 | A_33_P3331220 | 0.971792714 | 1.34E-07 |
| uc021thc.2 | A_33_P3331220 | 0.97071772 | 1.61E-07 |
| XR_429426.1 | A_33_P3331220 | 0.952035038 | 1.84E-06 |
| XR_429429.1 | A_33_P3331220 | 0.972884975 | 1.10E-07 |
| uc002sti.1 | A_33_P3331220 | 0.92428689 | 1.72E-05 |
| ENST00000530600.1 | PDGFC | -0.914820919 | 3.06E-05 |
| TCONS_00011690 | SUCNR1 | 0.935911923 | 7.64E-06 |
| ENST00000558120.1 | GUCY1B3 | 0.95823182 | 9.33E-07 |
| XR_241556.1 | GUCY1B3 | 0.922602313 | 1.92E-05 |
| LIT2061 | GUCY1B3 | 0.915771115 | 2.89E-05 |
| NR_027145.2 | GUCY1B3 | 0.961352653 | 6.36E-07 |
| ENST00000561134.1 | GUCY1B3 | 0.909138772 | 4.18E-05 |
| ENST00000420595.1 | GUCY1B3 | 0.953414448 | 1.60E-06 |
| XR_429366.1 | GUCY1B3 | 0.909314978 | 4.14E-05 |
| ENST00000442008.2 | GUCY1B3 | 0.915526157 | 2.94E-05 |
| XR_426860.1 | GUCY1B3 | 0.917734871 | 2.58E-05 |
| ENST00000602325.1 | GUCY1B3 | 0.943227689 | 4.22E-06 |
| LIT2094 | GUCY1B3 | 0.913296342 | 3.33E-05 |
| NR_037928.1 | GUCY1B3 | 0.915647745 | 2.92E-05 |
| ENST00000424116.2 | GUCY1B3 | 0.960101978 | 7.45E-07 |
| ENST00000607175.1 | GUCY1B3 | 0.950927285 | 2.06E-06 |
| uc022caj.1 | GUCY1B3 | 0.906008787 | 4.93E-05 |
| TCONS_00022516 | GUCY1B3 | 0.966047652 | 3.36E-07 |
| XR_426860.1 | SNN | 0.910167239 | 3.96E-05 |
| ENST00000532422.1 | ZNF706 | 0.963335058 | 4.91E-07 |
| ENST00000454737.1 | ZNF706 | 0.935669126 | 7.78E-06 |
| ENST00000599908.1 | A_33_P3283944 | -0.913126418 | 3.36E-05 |
| TCONS_00026998 | A_33_P3283944 | -0.903488124 | 5.60E-05 |
| ENST00000586885.1 | A_33_P3283944 | -0.937999969 | 6.50E-06 |
| ENST00000602814.1 | A_33_P3283944 | -0.93955921 | 5.74E-06 |
| ENST00000431928.1 | ALCAM | -0.913717809 | 3.25E-05 |
| ENST00000430228.1 | ALCAM | -0.934032038 | 8.80E-06 |
| ENST00000558120.1 | MTURN | 0.941250209 | 4.99E-06 |
| XR_241556.1 | MTURN | 0.943801038 | 4.02E-06 |
| NR_027145.2 | MTURN | 0.936364771 | 7.38E-06 |
| ENST00000561134.1 | MTURN | 0.90565329 | 5.02E-05 |
| ENST00000420595.1 | MTURN | 0.90272266 | 5.82E-05 |
| XR_426860.1 | MTURN | 0.933008172 | 9.49E-06 |
| ENST00000602325.1 | MTURN | 0.957552693 | 1.01E-06 |
| NR_037928.1 | MTURN | 0.922349818 | 1.95E-05 |
| ENST00000508021.1 | MTURN | 0.900676226 | 6.43E-05 |
| ENST00000424116.2 | MTURN | 0.94557207 | 3.43E-06 |
| ENST00000607175.1 | MTURN | 0.903766246 | 5.52E-05 |
| TCONS_00022516 | MTURN | 0.958822566 | 8.70E-07 |
| ENST00000558120.1 | RGS6 | 0.911469589 | 3.69E-05 |
| LIT2061 | RGS6 | 0.930829447 | 1.11E-05 |
| NR_027145.2 | RGS6 | 0.900751388 | 6.41E-05 |
| XR_429366.1 | RGS6 | 0.935162932 | 8.09E-06 |
| ENST00000592441.1 | RGS6 | 0.907313421 | 4.60E-05 |
| ENST00000506718.1 | RGS6 | 0.924519467 | 1.70E-05 |
| ENST00000607175.1 | RGS6 | 0.904762117 | 5.25E-05 |
| ENST00000556996.1 | RGS6 | 0.925583883 | 1.58E-05 |
| TCONS_00026411 | CDKN1A | 0.902467148 | 5.89E-05 |
| ENST00000445429.1 | CDKN1A | 0.913720206 | 3.25E-05 |
| ENST00000430620.1 | CDKN1A | 0.902289399 | 5.94E-05 |
| XR_427724.1 | CDKN1A | 0.910469646 | 3.89E-05 |
| ENST00000430228.1 | A_33_P3238479 | 0.938416039 | 6.29E-06 |
| ENST00000568248.1 | A_33_P3238479 | 0.921119357 | 2.10E-05 |
| uc002tzb.1 | A_33_P3238479 | 0.901598317 | 6.15E-05 |
| ENST00000600477.1 | ZAK | -0.907126499 | 4.65E-05 |
| ASO1647 | ZAK | 0.913683398 | 3.26E-05 |
| ENST00000558120.1 | MFAP3L | 0.913755358 | 3.25E-05 |
| LIT2061 | MFAP3L | 0.94186349 | 4.74E-06 |
| XR_426860.1 | MFAP3L | 0.902683676 | 5.83E-05 |
| ENST00000424116.2 | MFAP3L | 0.913204677 | 3.35E-05 |
| ENST00000556996.1 | MFAP3L | 0.913548702 | 3.28E-05 |
| TCONS_00022516 | MFAP3L | 0.956259249 | 1.17E-06 |
| ENST00000535911.1 | MT1X | -0.905818758 | 4.97E-05 |
| ENST00000599908.1 | TRMT13 | 0.929543129 | 1.21E-05 |
| ENST00000426704.1 | TRMT13 | 0.901211366 | 6.26E-05 |
| ENST00000425104.1 | TRMT13 | 0.909097388 | 4.19E-05 |
| ENST00000430694.1 | A_33_P3370019 | 0.927180817 | 1.43E-05 |
| RNA147249\|p0353_imsncRNA771 | A_33_P3370019 | 0.936021514 | 7.58E-06 |
| RNA147248\|p0352_imsncRNA771 | A_33_P3370019 | 0.931851686 | 1.03E-05 |
| ENST00000530194.1 | A_33_P3370019 | 0.925423065 | 1.60E-05 |
| ENST00000555918.1 | GFI1B | 0.901800846 | 6.09E-05 |
| NR_027145.2 | GFI1B | 0.931091078 | 1.09E-05 |
| ENST00000510536.1 | GFI1B | 0.904227825 | 5.39E-05 |
| ENST00000420595.1 | GFI1B | 0.919774424 | 2.28E-05 |
| XR_426860.1 | GFI1B | 0.932953583 | 9.53E-06 |
| ENST00000451706.2 | GFI1B | 0.909841107 | 4.03E-05 |
| ENST00000450238.1 | GFI1B | 0.906356839 | 4.84E-05 |
| TCONS_00010294 | GFI1B | 0.91476504 | 3.07E-05 |
| XR_427724.1 | GFI1B | 0.919195126 | 2.37E-05 |
| ENST00000439622.1 | lnc-AC092031.1-1 | 0.95464431 | 1.40E-06 |
| TCONS_00013892 | lnc-AC092031.1-1 | 0.957718179 | 9.91E-07 |
| TCONS_00013891 | lnc-AC092031.1-1 | 0.953448752 | 1.59E-06 |
| TCONS_00026223 | lnc-AC092031.1-1 | 0.917405916 | 2.63E-05 |
| ENST00000426704.1 | lnc-AC092031.1-1 | 0.922220413 | 1.97E-05 |
| ENST00000514571.1 | ZNF532 | 0.900699541 | 6.42E-05 |

# Table S4. lncRNA-targeted miRNAs predicted by miRcode

| **LncRNA** | **Targeted miRNAs** | **Pairs** |
| --- | --- | --- |
| BX004987.4 | hsa-miR-503, hsa-miR-7, hsa-miR-9, hsa-miR-93, hsa-miR-138, hsa-miR-139-5p, hsa-miR-140, hsa-miR-141, hsa-miR-142-3p, hsa-miR-145, hsa-miR-146, hsa-miR-150, hsa-miR-15, hsa-miR-183, hsa-let-7, hsa-miR-18, hsa-miR-191, hsa-miR-194, hsa-miR-19, hsa-miR-1, hsa-miR-203, hsa-miR-204, hsa-miR-205, hsa-miR-208, hsa-miR-214, hsa-miR-218, hsa-miR-22, hsa-miR-221, hsa-miR-223, hsa-miR-24, hsa-miR-26, hsa-miR-27, hsa-miR-29d, hsa-miR-103, hsa-miR-338, hsa-miR-375, hsa-miR-383, hsa-miR-10, hsa-miR-128, hsa-miR-129-5p, hsa-miR-490-3p, hsa-miR-499-5p | 42 |
| RP11-47J17.2 | hsa-miR-99, hsa-miR-140, hsa-miR-150, hsa-miR-17, hsa-miR-216, hsa-miR-10, hsa-miR-490-3p | 7 |
| RP11-429J17.8 | hsa-miR-503, hsa-miR-146, hsa-miR-199-5p, hsa-miR-22, hsa-miR-24, hsa-miR-27, hsa-miR-31, hsa-miR-128 | 8 |
| AC064834.2 | hsa-miR-7, hsa-miR-9, hsa-miR-135, hsa-miR-139-5p, hsa-miR-140, hsa-miR-144, hsa-miR-155, hsa-miR-216, hsa-miR-22, hsa-miR-101, hsa-miR-30def, hsa-miR-103, hsa-miR-129-5p | 13 |
| HLA-DQB1-AS1 | hsa-miR-9, hsa-miR-140, hsa-miR-145, hsa-miR-199-5p, hsa-miR-203, hsa-miR-205 | 6 |
| AC147651.3 | hsa-miR-551, hsa-miR-7, hsa-miR-133, hsa-miR-93, hsa-miR-96, hsa-miR-99, hsa-miR-138, hsa-miR-150, hsa-miR-17, hsa-miR-187, hsa-miR-191, hsa-miR-193, hsa-miR-203, hsa-miR-204, hsa-miR-24, hsa-miR-128, hsa-miR-93, hsa-miR-219-5p, hsa-miR-26, hsa-miR-27, hsa-miR-129-5p | 21 |
| RP11-445P17.8 | hsa-miR-133, hsa-miR-139-5p, hsa-miR-141, hsa-miR-196, hsa-miR-199-5p, hsa-miR-203, hsa-miR-216, hsa-miR-125-5p | 8 |
| AC007319.1 | hsa-miR-133, hsa-miR-9, hsa-miR-150, hsa-miR-196, hsa-miR-205, hsa-miR-214, hsa-miR-22, hsa-miR-23, hsa-miR-27, hsa-miR-30def, hsa-miR-383, hsa-miR-455-5p | 12 |
| RP11-235G24.1 | hsa-miR-148-3p, hsa-miR-192, hsa-miR-200, hsa-miR-205, hsa-miR-217, hsa-miR-33, hsa-miR-128 | 7 |
| RP1-18D14.4 | hsa-miR-137, hsa-miR-148-3p, hsa-miR-181d, hsa-miR-205, hsa-miR-218, hsa-miR-27, hsa-miR-34, hsa-miR-129-5p | 8 |
| AC017002.2 | hsa-miR-132, hsa-miR-138, hsa-miR-141, hsa-miR-181d, hsa-let-7, hsa-miR-187, hsa-miR-18, hsa-miR-194, hsa-miR-21, hsa-miR-27, hsa-miR-425, hsa-miR-128 | 12 |
| AC093818.1 | hsa-miR-503, hsa-miR-132, hsa-miR-133, hsa-miR-9, hsa-miR-96, hsa-miR-137, hsa-miR-141, hsa-miR-145, hsa-miR-15, hsa-miR-182, hsa-miR-183, hsa-let-7, hsa-miR-190, hsa-miR-192, hsa-miR-193, hsa-miR-194, hsa-miR-199-5p, hsa-miR-1, hsa-miR-21, hsa-miR-25, hsa-miR-26, hsa-miR-31, hsa-miR-103, hsa-miR-124, hsa-miR-33, hsa-miR-34, hsa-miR-129-5p, hsa-miR-490-3p | 28 |
| RP11-190J1.10 | hsa-miR-146, hsa-miR-191, hsa-miR-1, hsa-miR-223, hsa-miR-122, hsa-miR-23, hsa-miR-338, hsa-miR-375 | 8 |
| AC009531.2 | hsa-miR-7, hsa-miR-150, hsa-miR-19, hsa-miR-338, hsa-miR-34, hsa-miR-375 | 6 |
| AKAP11-IT1 | hsa-miR-7, hsa-miR-137, hsa-miR-144, hsa-miR-184, hsa-miR-22 | 5 |
| RP1-18D14.7 | hsa-miR-503, hsa-miR-130, hsa-miR-7, hsa-miR-133, hsa-miR-9, hsa-miR-137, hsa-miR-138, hsa-miR-141, hsa-miR-142-3p, hsa-miR-145, hsa-miR-148-3p, hsa-miR-150, hsa-miR-184, hsa-let-7, hsa-miR-194, hsa-miR-199-5p, hsa-miR-204, hsa-miR-205, hsa-miR-21, hsa-miR-216, hsa-miR-218, hsa-miR-22, hsa-miR-221, hsa-miR-24, hsa-miR-25, hsa-miR-124, hsa-miR-338, hsa-miR-33, hsa-miR-34, hsa-miR-383, hsa-miR-129-5p | 31 |
| RP3-323P24.3 | hsa-miR-17, hsa-miR-194, hsa-miR-214, hsa-miR-383 | 4 |
| AC105461.1 | hsa-miR-124 | 1 |
| RP11-162D16.2 | hsa-miR-138, hsa-miR-181d, hsa-miR-216, hsa-miR-27, hsa-miR-10, hsa-miR-129-5p | 6 |
| AC012501.2 | hsa-miR-130, hsa-miR-7, hsa-miR-138, hsa-miR-141, hsa-miR-143, hsa-miR-146, hsa-miR-148-3p, hsa-miR-15, hsa-miR-194, hsa-miR-196, hsa-miR-199-5p, hsa-miR-19, hsa-miR-216, hsa-miR-221, hsa-miR-27, hsa-miR-30def, hsa-miR-103, hsa-miR-375 | 17 |
| AP001619.2 | hsa-miR-7, hsa-miR-145, hsa-miR-194, hsa-miR-23 | 4 |
| RP1-122O8.7 | hsa-miR-140, hsa-miR-141, hsa-miR-142-3p, hsa-miR-145, hsa-miR-153, hsa-miR-19, hsa-miR-214, hsa-miR-216, hsa-miR-125-5p | 9 |
| AC017076.4 | hsa-miR-133, hsa-miR-9, hsa-miR-93, hsa-miR-96, hsa-miR-135, hsa-miR-138, hsa-miR-141, hsa-miR-143, hsa-miR-146, hsa-miR-148-3p, hsa-miR-150, hsa-miR-17, hsa-miR-181d, hsa-miR-182, hsa-miR-183, hsa-miR-192, hsa-miR-193, hsa-miR-199-5p, hsa-miR-1, hsa-miR-204, hsa-miR-205, hsa-miR-217, hsa-miR-218, hsa-miR-22, hsa-miR-122, hsa-miR-23, hsa-miR-24, hsa-miR-25, hsa-miR-26, hsa-miR-27, hsa-miR-31, hsa-miR-103, hsa-miR-33, hsa-miR-375, hsa-miR-383, hsa-miR-455-5p, hsa-miR-128, hsa-miR-129-5p | 38 |
| RP11-216B9.6 | hsa-miR-7, hsa-miR-141, hsa-miR-143, hsa-miR-193, hsa-miR-19, hsa-miR-219-5p, hsa-miR-22, hsa-miR-122, hsa-miR-24, hsa-miR-27, hsa-miR-375, hsa-miR-125-5p, hsa-miR-129-5p | 13 |
| AC093159.1 | hsa-miR-137, hsa-miR-183 | 2 |
| RP11-108M9.3 | hsa-miR-503, hsa-miR-551, hsa-miR-7, hsa-miR-133, hsa-miR-9, hsa-miR-93, hsa-miR-137, hsa-miR-138, hsa-miR-143, hsa-miR-146, hsa-miR-15, hsa-miR-18, hsa-miR-192, hsa-miR-193, hsa-miR-199-5p, hsa-miR-223, hsa-miR-24, hsa-miR-25, hsa-miR-30def, hsa-miR-125-5p | 20 |
| RP3-399L15.3 | hsa-miR-132, hsa-miR-7, hsa-miR-133, hsa-miR-9, hsa-miR-93, hsa-miR-96, hsa-miR-137, hsa-miR-138, hsa-miR-139-5p, hsa-miR-140, hsa-miR-141, hsa-miR-142-3p, hsa-miR-143, hsa-miR-145, hsa-miR-146, hsa-miR-150, hsa-miR-15, hsa-miR-17, hsa-miR-181d, hsa-miR-182, hsa-let-7, hsa-miR-187, hsa-miR-18, hsa-miR-194, hsa-miR-196, hsa-miR-1, hsa-miR-200, hsa-miR-203, hsa-miR-204, hsa-miR-205, hsa-miR-21, hsa-miR-214, hsa-miR-216, hsa-miR-217, hsa-miR-218, hsa-miR-219-5p, hsa-miR-221, hsa-miR-122, hsa-miR-24, hsa-miR-25, hsa-miR-26, hsa-miR-27, hsa-miR-30def, hsa-miR-103, hsa-miR-124, hsa-miR-338, hsa-miR-33-3p, hsa-miR-33, hsa-miR-375, hsa-miR-383, hsa-miR-125-5p, hsa-miR-129-5p | 52 |
| RP11-556E13.1 | hsa-miR-135, hsa-miR-140, hsa-miR-146, hsa-miR-150, hsa-miR-181d, hsa-miR-199-5p, hsa-miR-208, hsa-miR-29d, hsa-miR-10, hsa-miR-455-5p, hsa-miR-490-3p, hsa-miR-499-5p | 12 |
| TTTY21 | hsa-miR-137, hsa-miR-1, hsa-miR-27, hsa-miR-124, hsa-miR-34, hsa-miR-128 | 6 |
| RP11-175B12.2 | hsa-miR-9, hsa-miR-143, hsa-miR-1, hsa-miR-218, hsa-miR-23, hsa-miR-29d, hsa-miR-31, hsa-miR-10, hsa-miR-129-5p | 9 |
| RP11-384C4.6 | hsa-miR-7, hsa-miR-139-5p, hsa-miR-192, hsa-miR-193, hsa-miR-200, hsa-miR-383, hsa-miR-129-5p, hsa-miR-499-5p | 8 |
| RP11-14C22.6 | hsa-miR-216, hsa-miR-223, hsa-miR-33 | 3 |
| BX571672.1 | hsa-miR-7, hsa-miR-9, hsa-miR-138, hsa-miR-139-5p, hsa-miR-140, hsa-miR-142-3p, hsa-miR-145, hsa-miR-150, hsa-miR-15, hsa-miR-183, hsa-let-7, hsa-miR-194, hsa-miR-19, hsa-miR-1, hsa-miR-203, hsa-miR-214, hsa-miR-218, hsa-miR-219-5p, hsa-miR-223, hsa-miR-24, hsa-miR-26, hsa-miR-27, hsa-miR-29d, hsa-miR-103, hsa-miR-338, hsa-miR-375, hsa-miR-10, hsa-miR-128, hsa-miR-490-3p | 29 |
| AL583842.3 | hsa-miR-139-5p, hsa-miR-140, hsa-miR-146, hsa-miR-183, hsa-miR-194, hsa-miR-205, hsa-miR-208, hsa-miR-218, hsa-miR-26, hsa-miR-27, hsa-miR-29d, hsa-miR-338, hsa-miR-383, hsa-miR-128, hsa-miR-129-5p, hsa-miR-490-3p, hsa-miR-499-5p | 17 |
| RP4-569M23.2 | hsa-miR-132, hsa-miR-139-5p, hsa-miR-145, hsa-miR-153, hsa-miR-193, hsa-miR-194, hsa-miR-196, hsa-miR-19, hsa-miR-200, hsa-miR-204, hsa-miR-205, hsa-miR-214, hsa-miR-217, hsa-miR-29d, hsa-miR-103, hsa-miR-33, hsa-miR-375, hsa-miR-499-5p | 18 |
| RP5-1073O3.2 | hsa-miR-150, hsa-miR-204, hsa-miR-216, hsa-miR-23, hsa-miR-124, hsa-miR-125-5p, hsa-miR-129-5p | 7 |
| FARP1-AS1 | hsa-miR-7, hsa-miR-96, hsa-miR-182, hsa-miR-200, hsa-miR-205, hsa-miR-122, hsa-miR-27, hsa-miR-129-5p | 8 |
| AC096579.7 | hsa-miR-133, hsa-miR-141, hsa-miR-143, hsa-miR-199-5p, hsa-miR-19, hsa-miR-204, hsa-miR-214, hsa-miR-23, hsa-miR-26, hsa-miR-27, hsa-miR-125-5p, hsa-miR-128, hsa-miR-129-5p | 13 |
| RP11-368D24__A.1 | hsa-miR-503, hsa-miR-141, hsa-miR-143, hsa-miR-15, hsa-miR-214, hsa-miR-29d, hsa-miR-103, hsa-miR-34 | 8 |
| RPS6KA2-IT1 | hsa-miR-135, hsa-miR-184, hsa-miR-33, hsa-miR-34, hsa-miR-425, hsa-miR-128 | 6 |
| RP11-490N5.2 | hsa-miR-503, hsa-miR-7, hsa-miR-96, hsa-miR-137, hsa-miR-138, hsa-miR-139-5p, hsa-miR-141, hsa-miR-143, hsa-miR-146, hsa-miR-148-3p, hsa-miR-150, hsa-miR-153, hsa-miR-155, hsa-miR-15, hsa-miR-181d, hsa-miR-182, hsa-miR-183, hsa-let-7, hsa-miR-187, hsa-miR-18, hsa-miR-191, hsa-miR-194, hsa-miR-199-5p, hsa-miR-203, hsa-miR-21, hsa-miR-210, hsa-miR-214, hsa-miR-217, hsa-miR-218, hsa-miR-219-5p, hsa-miR-122, hsa-miR-23, hsa-miR-24, hsa-miR-25, hsa-miR-27, hsa-miR-29d, hsa-miR-33, hsa-miR-34, hsa-miR-375, hsa-miR-383, hsa-miR-425, hsa-miR-125-5p, hsa-miR-10, hsa-miR-451, hsa-miR-455-5p, hsa-miR-128, hsa-miR-129-5p, hsa-miR-490-3p | 48 |
| AC002480.3 | hsa-miR-130, hsa-miR-132, hsa-miR-133, hsa-miR-142-3p, hsa-miR-143, hsa-miR-144, hsa-miR-145, hsa-miR-146, hsa-miR-150, hsa-miR-17, hsa-miR-181d, hsa-miR-183, hsa-let-7, hsa-miR-193, hsa-miR-199-5p, hsa-miR-19, hsa-miR-1, hsa-miR-203, hsa-miR-208, hsa-miR-210, hsa-miR-221, hsa-miR-124, hsa-miR-425, hsa-miR-455-5p, hsa-miR-129-5p, hsa-miR-499-5p | 26 |
| AC078883.3 | hsa-miR-9, hsa-miR-1, hsa-miR-210, hsa-miR-30def, hsa-miR-375, hsa-miR-451 | 6 |
| AC002480.4 | hsa-miR-7, hsa-miR-150, hsa-miR-181d, hsa-let-7, hsa-miR-196, hsa-miR-122, hsa-miR-125-5p, hsa-miR-128, hsa-miR-490-3p | 9 |
| RP11-65J3.1 | hsa-miR-130, hsa-miR-135, hsa-miR-139-5p, hsa-miR-146, hsa-miR-148-3p, hsa-miR-17, hsa-miR-183, hsa-miR-214, hsa-miR-24, hsa-miR-338, hsa-miR-10, hsa-miR-128 | 12 |
| RP11-259P20.1 | hsa-miR-15, hsa-miR-181d, hsa-miR-1, hsa-miR-203, hsa-miR-216, hsa-miR-219-5p, hsa-miR-10 | 7 |
| RP5-887A10.1 | hsa-miR-132, hsa-miR-138, hsa-miR-141, hsa-miR-148-3p, hsa-miR-204, hsa-miR-216, hsa-miR-221, hsa-miR-24, hsa-miR-25 | 9 |
| AC019186.1 | hsa-miR-7, hsa-miR-153, hsa-miR-181d, hsa-miR-199-5p, hsa-miR-205, hsa-miR-27, hsa-miR-31, hsa-miR-128, hsa-miR-129-5p | 9 |
| LINC00163 | hsa-miR-7, hsa-miR-138, hsa-miR-143, hsa-miR-181d, hsa-miR-183, hsa-miR-18, hsa-miR-193, hsa-miR-194, hsa-miR-1, hsa-miR-203, hsa-miR-210, hsa-miR-214, hsa-miR-122, hsa-miR-27, hsa-miR-103, hsa-miR-124, hsa-miR-128 | 17 |
| RP11-384C4.7 | hsa-miR-135, hsa-miR-146, hsa-miR-216, hsa-miR-26 | 4 |
| RP4-756G23.5 | hsa-miR-7, hsa-miR-9, hsa-miR-93, hsa-miR-140, hsa-miR-141, hsa-miR-143, hsa-miR-144, hsa-miR-150, hsa-miR-155, hsa-miR-15, hsa-miR-17, hsa-let-7, hsa-miR-122, hsa-miR-27, hsa-miR-101, hsa-miR-103, hsa-miR-338, hsa-miR-34, hsa-miR-128, hsa-miR-129-5p | 20 |
| RP5-827C21.2 | hsa-miR-138, hsa-miR-193, hsa-miR-205 | 3 |
| RP1-118J21.5 | hsa-miR-146, hsa-miR-150, hsa-miR-18, hsa-miR-190, hsa-miR-196, hsa-miR-219-5p, hsa-miR-29d | 7 |
| LINC00299 | hsa-miR-132, hsa-miR-7, hsa-miR-133, hsa-miR-135, hsa-miR-137, hsa-miR-138, hsa-miR-139-5p, hsa-miR-145, hsa-miR-146, hsa-miR-182, hsa-miR-190, hsa-miR-196, hsa-miR-199-5p, hsa-miR-19, hsa-miR-1, hsa-miR-200, hsa-miR-203, hsa-miR-204, hsa-miR-205, hsa-miR-208, hsa-miR-21, hsa-miR-210, hsa-miR-217, hsa-miR-218, hsa-miR-221, hsa-miR-223, hsa-miR-23, hsa-miR-24, hsa-miR-31, hsa-miR-338, hsa-miR-33-3p, hsa-miR-383, hsa-miR-128, hsa-miR-490-3p, hsa-miR-499-5p | 35 |
| AL592284.1 | hsa-miR-7, hsa-miR-133, hsa-miR-9, hsa-miR-137, hsa-miR-138, hsa-miR-139-5p, hsa-miR-141, hsa-miR-143, hsa-miR-144, hsa-miR-145, hsa-miR-146, hsa-miR-153, hsa-miR-15, hsa-miR-17, hsa-miR-183, hsa-miR-18, hsa-miR-190, hsa-miR-191, hsa-miR-193, hsa-miR-199-5p, hsa-miR-1, hsa-miR-200, hsa-miR-203, hsa-miR-204, hsa-miR-205, hsa-miR-214, hsa-miR-216, hsa-miR-217, hsa-miR-218, hsa-miR-22, hsa-miR-221, hsa-miR-223, hsa-miR-122, hsa-miR-23, hsa-miR-24, hsa-miR-25, hsa-miR-26, hsa-miR-27, hsa-miR-30def, hsa-miR-31, hsa-miR-103, hsa-miR-33, hsa-miR-34, hsa-miR-425, hsa-miR-10, hsa-miR-128, hsa-miR-129-5p | 47 |
| GLIS3-AS1 | hsa-miR-99, hsa-miR-140, hsa-miR-18, hsa-miR-196, hsa-miR-1, hsa-miR-23, hsa-miR-24, hsa-miR-124, hsa-miR-338, | 9 |
| RP11-885N19.6 | hsa-miR-26 | 1 |
| LINC00211 | hsa-miR-130, hsa-miR-132, hsa-miR-9, hsa-miR-139-5p, hsa-miR-148-3p, hsa-miR-153, hsa-miR-17, hsa-miR-181d, hsa-miR-183, hsa-miR-184, hsa-miR-203, hsa-miR-204, hsa-miR-214, hsa-miR-216, hsa-miR-219-5p, hsa-miR-23, hsa-miR-27, hsa-miR-103, hsa-miR-124, hsa-miR-338, hsa-miR-375, hsa-miR-383 | 22 |
| GCFC1-AS1 | hsa-miR-133, hsa-miR-9, hsa-miR-138, hsa-miR-139-5p, hsa-miR-143, hsa-miR-144, hsa-miR-15, hsa-miR-181d, hsa-miR-182, hsa-miR-183, hsa-miR-192, hsa-miR-194, hsa-miR-199-5p, hsa-miR-19, hsa-miR-200, hsa-miR-203, hsa-miR-205, hsa-miR-210, hsa-miR-216, hsa-miR-223, hsa-miR-122, hsa-miR-23, hsa-miR-24, hsa-miR-25, hsa-miR-26, hsa-miR-27, hsa-miR-101, hsa-miR-29d, hsa-miR-30def, hsa-miR-124, hsa-miR-338, hsa-miR-33-3p, hsa-miR-375, hsa-miR-425, hsa-miR-125-5p, hsa-miR-10, hsa-miR-128, hsa-miR-129-5p | 38 |
| RP11-185E8.1 | hsa-miR-7, hsa-miR-141, hsa-miR-1, hsa-miR-205, hsa-miR-23 | 5 |
| XXbac-BPG254F23.6.3 | hsa-miR-143, hsa-miR-148-3p, hsa-miR-218 | 3 |
| RP11-511P7.2 | hsa-miR-130, hsa-miR-7, hsa-miR-9, hsa-miR-135, hsa-miR-137, hsa-miR-138, hsa-miR-139-5p, hsa-miR-143, hsa-miR-144, hsa-miR-17, hsa-miR-183, hsa-miR-19, hsa-miR-214, hsa-miR-219-5p, hsa-miR-24, hsa-miR-25, hsa-miR-27, hsa-miR-101, hsa-miR-31, hsa-miR-103, hsa-miR-124, hsa-miR-338, hsa-miR-34, hsa-miR-128 | 24 |
| AC093627.8 | hsa-miR-7, hsa-miR-15, hsa-miR-214, hsa-miR-22, hsa-miR-26 | 5 |
| AL109761.5 | hsa-miR-503, hsa-miR-130, hsa-miR-9, hsa-miR-138, hsa-miR-141, hsa-miR-153, hsa-miR-15, hsa-miR-204, hsa-miR-124 | 9 |
| RP11-492A10.1 | hsa-miR-138, hsa-miR-153, hsa-miR-155, hsa-miR-190, hsa-miR-199-5p, hsa-miR-203, hsa-miR-204, hsa-miR-205, hsa-miR-216, hsa-miR-223, hsa-miR-29d | 11 |
| RP13-578N3.3 | hsa-miR-143, hsa-miR-124 | 2 |
| RP11-159H10.3 | hsa-miR-135, hsa-miR-138, hsa-miR-142-3p, hsa-miR-145, hsa-let-7, hsa-miR-193, hsa-miR-1, hsa-miR-203, hsa-miR-214, hsa-miR-219-5p, hsa-miR-22, hsa-miR-23, hsa-miR-26, hsa-miR-27, hsa-miR-128, hsa-miR-129-5p | 16 |
| CTC-463N11.3 | hsa-miR-503, hsa-miR-551, hsa-miR-7, hsa-miR-143, hsa-miR-150, hsa-miR-181d, hsa-miR-217, hsa-miR-22, hsa-miR-122, hsa-miR-24, hsa-miR-25, hsa-miR-31, hsa-miR-103, hsa-miR-33, hsa-miR-34, hsa-miR-128, hsa-miR-129-5p, hsa-miR-499-5p | 18 |
| RP11-730K11.1 | hsa-miR-132, hsa-miR-7, hsa-miR-9, hsa-miR-93, hsa-miR-96, hsa-miR-138, hsa-miR-155, hsa-miR-182, hsa-miR-187, hsa-miR-193, hsa-miR-194, hsa-miR-199-5p, hsa-miR-1, hsa-miR-203, hsa-miR-204, hsa-miR-205, hsa-miR-208, hsa-miR-214, hsa-miR-122, hsa-miR-24, hsa-miR-25, hsa-miR-103, hsa-miR-124, hsa-miR-33-3p, hsa-miR-33, hsa-miR-34, hsa-miR-455-5p, hsa-miR-129-5p, hsa-miR-499-5p | 29 |
| RP11-296I10.3 | hsa-miR-7, hsa-miR-133, hsa-miR-9, hsa-miR-144, hsa-miR-146, hsa-miR-148-3p, hsa-miR-153, hsa-miR-183, hsa-miR-18, hsa-miR-204, hsa-miR-205, hsa-miR-208, hsa-miR-214, hsa-miR-216, hsa-miR-218, hsa-miR-223, hsa-miR-24, hsa-miR-27, hsa-miR-103, hsa-miR-338, hsa-miR-34, hsa-miR-128, hsa-miR-499-5p | 23 |
| CTC-454M9.1 | hsa-miR-132, hsa-miR-7, hsa-miR-133, hsa-miR-135, hsa-miR-137, hsa-miR-141, hsa-miR-145, hsa-miR-146, hsa-miR-17, hsa-miR-181d, hsa-miR-183, hsa-let-7, hsa-miR-190, hsa-miR-192, hsa-miR-196, hsa-miR-200, hsa-miR-203, hsa-miR-204, hsa-miR-205, hsa-miR-208, hsa-miR-210, hsa-miR-214, hsa-miR-216, hsa-miR-217, hsa-miR-219-5p, hsa-miR-22, hsa-miR-223, hsa-miR-23, hsa-miR-24, hsa-miR-27, hsa-miR-30def, hsa-miR-103, hsa-miR-124, hsa-miR-338, hsa-miR-33-3p, hsa-miR-33, hsa-miR-425, hsa-miR-10, hsa-miR-455-5p, hsa-miR-128, hsa-miR-129-5p, hsa-miR-490-3p, hsa-miR-499-5p | 43 |
| RP11-597D13.9 | hsa-miR-130, hsa-miR-7, hsa-miR-133, hsa-miR-9, hsa-miR-96, hsa-miR-135, hsa-miR-137, hsa-miR-141, hsa-miR-142-3p, hsa-miR-143, hsa-miR-144, hsa-miR-150, hsa-miR-155, hsa-miR-17, hsa-miR-181d, hsa-miR-183, hsa-miR-187, hsa-miR-18, hsa-miR-190, hsa-miR-19, hsa-miR-1, hsa-miR-200, hsa-miR-203, hsa-miR-205, hsa-miR-21, hsa-miR-210, hsa-miR-214, hsa-miR-216, hsa-miR-217, hsa-miR-218, hsa-miR-22, hsa-miR-122, hsa-miR-23, hsa-miR-24, hsa-miR-25, hsa-miR-27, hsa-miR-101, hsa-miR-29d, hsa-miR-30def, hsa-miR-31, hsa-miR-103, hsa-miR-338, hsa-miR-33, hsa-miR-425, hsa-miR-125-5p, hsa-miR-10, hsa-miR-455-5p, hsa-miR-490-3p | 48 |
| RP11-768F21.1 | hsa-miR-143, hsa-miR-15, hsa-miR-18, hsa-miR-214, hsa-miR-25, hsa-miR-31 | 6 |
| RP11-438E5.1 | hsa-miR-130, hsa-miR-132, hsa-miR-133, hsa-miR-93, hsa-miR-138, hsa-miR-142-3p, hsa-miR-144, hsa-miR-146, hsa-miR-150, hsa-miR-181d, hsa-miR-183, hsa-miR-184, hsa-miR-19, hsa-miR-1, hsa-miR-200, hsa-miR-204, hsa-miR-205, hsa-miR-214, hsa-miR-216, hsa-miR-218, hsa-miR-122, hsa-miR-24, hsa-miR-26, hsa-miR-30def, hsa-miR-103, hsa-miR-338, hsa-miR-34, hsa-miR-375, hsa-miR-455-5p, hsa-miR-129-5p, hsa-miR-490-3p | 31 |
| PVT1 | hsa-miR-503, hsa-miR-551, hsa-miR-7, hsa-miR-133, hsa-miR-9, hsa-miR-93, hsa-miR-139-5p, hsa-miR-140, hsa-miR-143, hsa-miR-145, hsa-miR-148-3p, hsa-miR-150, hsa-miR-15, hsa-miR-17, hsa-miR-181d, hsa-miR-183, hsa-miR-187, hsa-miR-18, hsa-miR-190, hsa-miR-194, hsa-miR-199-5p, hsa-miR-203, hsa-miR-205, hsa-miR-21, hsa-miR-214, hsa-miR-216, hsa-miR-217, hsa-miR-221, hsa-miR-23, hsa-miR-24, hsa-miR-27, hsa-miR-29d, hsa-miR-30def, hsa-miR-31, hsa-miR-124, hsa-miR-33-3p, hsa-miR-34, hsa-miR-383, hsa-miR-455-5p, hsa-miR-128, hsa-miR-490-3p | 41 |
| RP11-476C8.3 | hsa-miR-551, hsa-miR-130, hsa-miR-141, hsa-miR-148-3p, hsa-miR-192, hsa-miR-200, hsa-miR-203, hsa-miR-21, hsa-miR-210, hsa-miR-22, hsa-miR-24, hsa-miR-25, hsa-miR-30def, hsa-miR-124, hsa-miR-33, hsa-miR-34, hsa-miR-10 | 17 |
| RP11-301H24.4 | hsa-miR-9, hsa-miR-139-5p, hsa-miR-150, hsa-miR-18, hsa-miR-1, hsa-miR-22, hsa-miR-103, hsa-miR-338 | 8 |
| U66061.31 | hsa-miR-130, hsa-miR-7, hsa-miR-93, hsa-miR-138, hsa-miR-140, hsa-miR-141, hsa-miR-142-3p, hsa-miR-145, hsa-miR-146, hsa-miR-148-3p, hsa-miR-150, hsa-miR-17, hsa-miR-181d, hsa-let-7, hsa-miR-193, hsa-miR-19, hsa-miR-214, hsa-miR-24, hsa-miR-30def, hsa-miR-31, hsa-miR-103, hsa-miR-33, hsa-miR-34, hsa-miR-125-5p, hsa-miR-128, hsa-miR-129-5p, hsa-miR-490-3p, hsa-miR-499-5p | 28 |
| RP11-53O19.1 | hsa-miR-9, hsa-miR-135, hsa-miR-145, hsa-miR-153, hsa-let-7, hsa-miR-18, hsa-miR-190, hsa-miR-217, hsa-miR-218, hsa-miR-223, hsa-miR-23, hsa-miR-27, hsa-miR-30def, hsa-miR-31, hsa-miR-103, hsa-miR-375, hsa-miR-10, hsa-miR-128 | 18 |
| RP11-689K5.3 | hsa-miR-150, hsa-miR-193, hsa-miR-204, hsa-miR-205, hsa-miR-24, hsa-miR-129-5p | 6 |
| RP11-109E24.1 | hsa-miR-9, hsa-miR-137, hsa-miR-145, hsa-miR-199-5p, hsa-miR-203, hsa-miR-219-5p, hsa-miR-25, hsa-miR-30def, hsa-miR-425 | 9 |
| RP11-673E1.1 | hsa-miR-130, hsa-miR-133, hsa-miR-96, hsa-miR-141, hsa-miR-150, hsa-miR-153, hsa-miR-15, hsa-miR-181d, hsa-miR-182, hsa-miR-203, hsa-miR-214, hsa-miR-216, hsa-miR-219-5p, hsa-miR-23, hsa-miR-26, hsa-miR-27, hsa-miR-30def, hsa-miR-103, hsa-miR-124, hsa-miR-338, hsa-miR-383, hsa-miR-125-5p, hsa-miR-128, hsa-miR-129-5p | 24 |
| LINC00534 | hsa-miR-7, hsa-miR-93, hsa-miR-96, hsa-miR-139-5p, hsa-miR-150, hsa-miR-192, hsa-miR-205, hsa-miR-217, hsa-miR-22, hsa-miR-24, hsa-miR-27, hsa-miR-425 | 12 |
| KB-1460A1.3 | hsa-miR-142-3p, hsa-miR-23, hsa-miR-24, hsa-miR-27, hsa-miR-31, hsa-miR-338, hsa-miR-34, hsa-miR-128 | 8 |
| AC005895.3 | hsa-miR-135, hsa-miR-138, hsa-miR-141, hsa-miR-150, hsa-let-7, hsa-miR-194, hsa-miR-19, hsa-miR-204, hsa-miR-214, hsa-miR-217, hsa-miR-24, hsa-miR-125-5p | 12 |
| RP11-45K10.2 | hsa-miR-7, hsa-miR-93, hsa-miR-137, hsa-miR-138, hsa-miR-141, hsa-miR-143, hsa-miR-144, hsa-miR-153, hsa-miR-17, hsa-miR-181d, hsa-miR-183, hsa-miR-190, hsa-miR-196, hsa-miR-199-5p, hsa-miR-19, hsa-miR-204, hsa-miR-21, hsa-miR-216, hsa-miR-22, hsa-miR-221, hsa-miR-122, hsa-miR-23, hsa-miR-24, hsa-miR-26, hsa-miR-30def, hsa-miR-103, hsa-miR-338, hsa-miR-33, hsa-miR-129-5p | 29 |
| CTD-2336O2.1 | hsa-miR-132, hsa-miR-148-3p, hsa-miR-150, hsa-miR-203, hsa-miR-124, hsa-miR-34, hsa-miR-125-5p, hsa-miR-129-5p | 8 |
| RP11-677I18.3 | hsa-miR-144, hsa-miR-17, hsa-miR-182, hsa-miR-18, hsa-miR-200, hsa-miR-203, hsa-miR-204, hsa-miR-205, hsa-miR-214, hsa-miR-216, hsa-miR-218, hsa-miR-221, hsa-miR-27, hsa-miR-101, hsa-miR-29d, hsa-miR-338, hsa-miR-425, hsa-miR-129-5p | 18 |
| RP11-872D17.4 | hsa-miR-24, hsa-miR-33-3p | 2 |
| CTD-2560E9.3 | hsa-miR-15, hsa-miR-214, hsa-miR-24, hsa-miR-29d, hsa-miR-338, hsa-miR-128 | 6 |
| RP11-672A2.4 | hsa-miR-133, hsa-miR-15, hsa-let-7, hsa-miR-122, hsa-miR-128 | 5 |
| RP11-661A12.5 | hsa-miR-1, hsa-miR-210, hsa-miR-30def | 3 |
| NAV2-AS5 | hsa-miR-130, hsa-miR-7, hsa-miR-133, hsa-miR-93, hsa-miR-135, hsa-miR-141, hsa-miR-143, hsa-miR-144, hsa-miR-150, hsa-miR-182, hsa-miR-193, hsa-miR-19, hsa-miR-1, hsa-miR-203, hsa-miR-205, hsa-miR-216, hsa-miR-217, hsa-miR-219-5p, hsa-miR-122, hsa-miR-23, hsa-miR-26, hsa-miR-101, hsa-miR-30def, hsa-miR-338, hsa-miR-33-3p, hsa-miR-34, hsa-miR-383, hsa-miR-425, hsa-miR-455-5p, | 29 |
| RP11-219O3.2 | hsa-miR-135, hsa-miR-27, hsa-miR-29d, hsa-miR-499-5p | 4 |
| RP11-514F3.5.1 | hsa-miR-141, hsa-miR-10 | 2 |
| RP11-142C4.6 | hsa-miR-132, hsa-miR-135, hsa-miR-143, hsa-miR-144, hsa-miR-145, hsa-miR-146, hsa-miR-148-3p, hsa-miR-150, hsa-miR-153, hsa-miR-15, hsa-miR-183, hsa-miR-193, hsa-miR-1, hsa-miR-200, hsa-miR-203, hsa-miR-204, hsa-miR-205, hsa-miR-208, hsa-miR-214, hsa-miR-218, hsa-miR-219-5p, hsa-miR-221, hsa-miR-223, hsa-miR-23, hsa-miR-24, hsa-miR-27, hsa-miR-101, hsa-miR-103, hsa-miR-124, hsa-miR-338, hsa-miR-33, hsa-miR-34, hsa-miR-425, hsa-miR-125-5p, hsa-miR-10, hsa-miR-128, hsa-miR-490-3p, hsa-miR-499-5p | 38 |
| C8orf77 | hsa-miR-503, hsa-miR-132, hsa-miR-7, hsa-miR-133, hsa-miR-9, hsa-miR-93, hsa-miR-135, hsa-miR-140, hsa-miR-142-3p, hsa-miR-143, hsa-miR-148-3p, hsa-miR-155, hsa-miR-15, hsa-miR-194, hsa-miR-203, hsa-miR-204, hsa-miR-21, hsa-miR-217, hsa-miR-122, hsa-miR-31, hsa-miR-103, hsa-miR-33-3p, hsa-miR-425, hsa-miR-10, hsa-miR-128, hsa-miR-490-3p | 26 |
| RP11-173C20.2 | hsa-miR-135, hsa-miR-143, hsa-miR-183, hsa-miR-184, hsa-miR-194, hsa-miR-199-5p, hsa-miR-200, hsa-miR-21, hsa-miR-210, hsa-miR-26, hsa-miR-27, hsa-miR-31, hsa-miR-338, hsa-miR-34, hsa-miR-425, hsa-miR-10 | 16 |
| RP11-277P12.9 | hsa-miR-7, hsa-miR-133, hsa-miR-9, hsa-miR-135, hsa-miR-140, hsa-miR-18, hsa-miR-190, hsa-miR-199-5p, hsa-miR-122 | 9 |
| RP11-116G8.5 | hsa-miR-143, hsa-miR-184, hsa-miR-128 | 3 |
| RP5-944M2.2 | hsa-miR-133, hsa-miR-137, hsa-miR-155, hsa-miR-190, hsa-miR-210, hsa-miR-338, hsa-miR-33-3p, hsa-miR-375 | 8 |
| RP3-405J10.3 | hsa-miR-132, hsa-miR-9, hsa-miR-93, hsa-miR-96, hsa-miR-135, hsa-miR-138, hsa-miR-141, hsa-miR-143, hsa-miR-144, hsa-miR-148-3p, hsa-miR-150, hsa-miR-153, hsa-miR-15, hsa-miR-17, hsa-miR-181d, hsa-let-7, hsa-miR-192, hsa-miR-199-5p, hsa-miR-1, hsa-miR-200, hsa-miR-203, hsa-miR-204, hsa-miR-205, hsa-miR-214, hsa-miR-216, hsa-miR-217, hsa-miR-22, hsa-miR-122, hsa-miR-23, hsa-miR-24, hsa-miR-26, hsa-miR-27, hsa-miR-103, hsa-miR-338, hsa-miR-33, hsa-miR-34, hsa-miR-375, hsa-miR-125-5p, hsa-miR-10, hsa-miR-128, hsa-miR-129-5p | 41 |
| RP1-197B17.3 | hsa-miR-130, hsa-miR-7, hsa-miR-93, hsa-miR-138, hsa-miR-142-3p, hsa-miR-143, hsa-miR-144, hsa-miR-150, hsa-miR-15, hsa-miR-17, hsa-miR-181d, hsa-miR-199-5p, hsa-miR-19, hsa-miR-203, hsa-miR-204, hsa-miR-210, hsa-miR-216, hsa-miR-217, hsa-miR-218, hsa-miR-24, hsa-miR-25, hsa-miR-26, hsa-miR-27, hsa-miR-29d, hsa-miR-338, hsa-miR-33-3p, hsa-miR-34, hsa-miR-375, hsa-miR-425, hsa-miR-125-5p, hsa-miR-10, hsa-miR-455-5p, hsa-miR-128, hsa-miR-490-3p | 34 |
| RP11-384J4.1 | hsa-miR-130, hsa-miR-132, hsa-miR-142-3p, hsa-miR-144, hsa-miR-148-3p, hsa-miR-150, hsa-miR-155, hsa-miR-187, hsa-miR-192, hsa-miR-193, hsa-miR-19, hsa-miR-200, hsa-miR-203, hsa-miR-204, hsa-miR-101, hsa-miR-34, hsa-miR-125-5p | 17 |
| RP11-616L12.1 | hsa-miR-132, hsa-miR-138, hsa-miR-23, hsa-miR-33, hsa-miR-129-5p | 5 |
| RP11-70F11.6 | hsa-miR-503, hsa-miR-96, hsa-miR-135, hsa-miR-138, hsa-miR-140, hsa-miR-146, hsa-miR-15, hsa-miR-182, hsa-miR-18, hsa-miR-200, hsa-miR-205, hsa-miR-216, hsa-miR-218, hsa-miR-219-5p, hsa-miR-223, hsa-miR-24, hsa-miR-25, hsa-miR-30def, hsa-miR-338, hsa-miR-383, hsa-miR-125-5p, hsa-miR-10, hsa-miR-128, hsa-miR-129-5p, hsa-miR-490-3p | 25 |
| RP11-76E17.3 | hsa-miR-130, hsa-miR-93, hsa-miR-138, hsa-miR-146, hsa-miR-153, hsa-miR-194, hsa-miR-199-5p, hsa-miR-203, hsa-miR-205, hsa-miR-217, hsa-miR-24, hsa-miR-34, hsa-miR-125-5p, hsa-miR-128 | 14 |
| RP11-16B13.1 | hsa-miR-96, hsa-miR-182, hsa-miR-199-5p, hsa-miR-204, hsa-miR-219-5p, hsa-miR-375 | 6 |
| RP11-701B16.3 | hsa-miR-203, hsa-miR-122, hsa-miR-27, hsa-miR-128 | 4 |
| RP11-1033H12.1 | hsa-miR-7, hsa-miR-139-5p | 2 |
| RP11-701B16.2 | hsa-miR-133, hsa-miR-144, hsa-miR-25, hsa-miR-26, hsa-miR-27, hsa-miR-101, hsa-miR-129-5p | 7 |
| LINC00520 | hsa-miR-503, hsa-miR-93, hsa-miR-135, hsa-miR-142-3p, hsa-miR-145, hsa-miR-150, hsa-miR-15, hsa-miR-17, hsa-miR-181d, hsa-let-7, hsa-miR-194, hsa-miR-19, hsa-miR-203, hsa-miR-204, hsa-miR-205, hsa-miR-216, hsa-miR-217, hsa-miR-223, hsa-miR-23, hsa-miR-24, hsa-miR-31, hsa-miR-124, hsa-miR-375, hsa-miR-125-5p, hsa-miR-129-5p | 25 |
| RP11-300J18.3.1 | hsa-miR-135, hsa-miR-137, hsa-miR-138, hsa-miR-143, hsa-miR-146, hsa-miR-150, hsa-miR-15, hsa-miR-187, hsa-miR-190, hsa-miR-196, hsa-miR-1, hsa-miR-205, hsa-miR-214, hsa-miR-216, hsa-miR-219-5p, hsa-miR-25, hsa-miR-27, hsa-miR-33, hsa-miR-375, hsa-miR-383, hsa-miR-129-5p | 21 |
| RP11-317N8.5 | hsa-miR-551, hsa-miR-130, hsa-miR-132, hsa-miR-9, hsa-miR-93, hsa-miR-143, hsa-miR-145, hsa-miR-150, hsa-miR-17, hsa-miR-183, hsa-miR-18, hsa-miR-205, hsa-miR-216, hsa-miR-217, hsa-miR-22, hsa-miR-122, hsa-miR-23, hsa-miR-338, hsa-miR-383, hsa-miR-125-5p, hsa-miR-126-3p, hsa-miR-129-5p | 22 |
| RP11-930O11.2 | hsa-miR-96, hsa-miR-143, hsa-miR-145, hsa-miR-17, hsa-miR-182, hsa-miR-18, hsa-miR-205, hsa-miR-21, hsa-miR-27, hsa-miR-375, hsa-miR-128, hsa-miR-129-5p | 12 |
| RP11-111A22.1 | hsa-miR-9, hsa-miR-96, hsa-miR-135, hsa-miR-146, hsa-miR-148-3p, hsa-miR-181d, hsa-miR-182, hsa-let-7, hsa-miR-191, hsa-miR-194, hsa-miR-199-5p, hsa-miR-200, hsa-miR-204, hsa-miR-214, hsa-miR-217, hsa-miR-122, hsa-miR-25, hsa-miR-375, hsa-miR-383, hsa-miR-451, hsa-miR-455-5p, hsa-miR-129-5p | 22 |
| RP5-977B1.11 | hsa-miR-7, hsa-miR-122, hsa-miR-27, hsa-miR-383, hsa-miR-125-5p, hsa-miR-128 | 6 |
| RP11-930O11.1 | hsa-miR-9, hsa-miR-96, hsa-miR-145, hsa-miR-146, hsa-miR-181d, hsa-miR-182, hsa-miR-196, hsa-miR-199-5p, hsa-miR-214, hsa-miR-29d, hsa-miR-103, hsa-miR-33, hsa-miR-34 | 13 |
| RP11-59H7.3 | hsa-miR-139-5p, hsa-miR-141, hsa-miR-18, hsa-miR-203, hsa-miR-205, hsa-miR-126-3p, hsa-miR-455-5p, hsa-miR-129-5p | 8 |
| AC083843.1 | hsa-miR-132, hsa-miR-9, hsa-miR-93, hsa-miR-96, hsa-miR-99, hsa-miR-138, hsa-miR-139-5p, hsa-miR-141, hsa-miR-143, hsa-miR-144, hsa-miR-145, hsa-miR-146, hsa-miR-148-3p, hsa-miR-150, hsa-miR-153, hsa-miR-155, hsa-miR-17, hsa-miR-182, hsa-miR-183, hsa-miR-194, hsa-miR-1, hsa-miR-200, hsa-miR-203, hsa-miR-204, hsa-miR-205, hsa-miR-208, hsa-miR-21, hsa-miR-216, hsa-miR-217, hsa-miR-218, hsa-miR-23, hsa-miR-25, hsa-miR-26, hsa-miR-27, hsa-miR-101, hsa-miR-29d, hsa-miR-30def, hsa-miR-31, hsa-miR-103, hsa-miR-124, hsa-miR-33, hsa-miR-34, hsa-miR-383, hsa-miR-125-5p, hsa-miR-455-5p, hsa-miR-128, hsa-miR-129-5p, hsa-miR-490-3p, hsa-miR-499-5p | 49 |
| RP11-568N6.1 | hsa-miR-132, hsa-miR-96, hsa-miR-140, hsa-miR-17, hsa-miR-182, hsa-miR-19, hsa-miR-200, hsa-miR-203, hsa-miR-204, hsa-miR-214, hsa-miR-219-5p, hsa-miR-223, hsa-miR-122, hsa-miR-338 | 14 |
| RP11-366L5.1 | hsa-miR-130, hsa-miR-93, hsa-miR-96, hsa-miR-135, hsa-miR-137, hsa-miR-138, hsa-miR-150, hsa-miR-15, hsa-miR-17, hsa-miR-181d, hsa-miR-182, hsa-miR-183, hsa-miR-191, hsa-miR-192, hsa-miR-194, hsa-miR-196, hsa-miR-1, hsa-miR-200, hsa-miR-203, hsa-miR-204, hsa-miR-216, hsa-miR-218, hsa-miR-22, hsa-miR-122, hsa-miR-23, hsa-miR-25, hsa-miR-26, hsa-miR-103, hsa-miR-34, hsa-miR-375, hsa-miR-383, hsa-miR-129-5p, hsa-miR-490-3p | 33 |
| AC137934.1 | hsa-miR-7, hsa-miR-133, hsa-miR-135, hsa-miR-139-5p, hsa-miR-141, hsa-miR-143, hsa-miR-144, hsa-miR-146, hsa-miR-181d, hsa-miR-18, hsa-miR-19, hsa-miR-1, hsa-miR-200, hsa-miR-205, hsa-miR-217, hsa-miR-218, hsa-miR-221, hsa-miR-223, hsa-miR-122, hsa-miR-24, hsa-miR-26, hsa-miR-27, hsa-miR-101, hsa-miR-33-3p, hsa-miR-33, hsa-miR-34, hsa-miR-490-3p | 27 |
| RP11-77H9.8 | hsa-miR-93, hsa-miR-18, hsa-miR-30def | 3 |
| RP11-104N10.1 | hsa-miR-133, hsa-miR-96, hsa-miR-143, hsa-miR-148-3p, hsa-miR-210, hsa-miR-214, hsa-miR-27, hsa-miR-34, hsa-miR-375, hsa-miR-129-5p | 10 |
| RP11-326A19.3 | hsa-miR-7, hsa-miR-181d, hsa-miR-221, hsa-miR-383 | 4 |
| RP11-399O19.8 | hsa-miR-7, hsa-miR-93, hsa-miR-145, hsa-miR-150, hsa-miR-17, hsa-miR-182, hsa-miR-193, hsa-miR-1, hsa-miR-200, hsa-miR-203, hsa-miR-24, hsa-miR-338, hsa-miR-10, | 13 |

# Table S5. miRNA-targeted mRNAs predicted by miRDB, miRTarBase, and TargetScan

| **mRNAs** | **miRNAs** | **Pairs** |
| --- | --- | --- |
| MITF | hsa-miR-182-5p, hsa-miR-340-5p | 2 |
| CTSA | hsa-miR-17-5p | 1 |
| SNCA | hsa-miR-7-5p | 1 |
| CDKN1A | hsa-let-7e-5p, hsa-let-7f-5p, hsa-miR-106a-5p, hsa-miR-519d-3p, hsa-miR-4458, hsa-miR-17-5p | 6 |
| FASLG | hsa-let-7e-5p, hsa-miR-149-5p | 2 |
| PRLR | hsa-miR-218-5p | 1 |
| ACER2 | hsa-let-7a-5p, hsa-let-7d-5p, hsa-let-7f-5p, hsa-let-7g-5p, hsa-let-7i-5p, hsa-miR-4458, hsa-miR-4500 | 7 |
| AKTIP | hsa-miR-17-5p, hsa-miR-20a-5p, hsa-miR-106b-5p, hsa-miR-20b-5p | 4 |
| TFPI | hsa-miR-27a-3p | 1 |
| CREBZF | hsa-miR-26b-5p, hsa-miR-299-5p, hsa-miR-425-5p, hsa-miR-26a-5p | 4 |
| IQSEC2 | hsa-miR-28-5p | 1 |
| PLSCR4 | hsa-miR-16-5p | 1 |
| ELOVL6 | hsa-miR-92a-3p, hsa-miR-211-5p, hsa-miR-204-5p | 3 |
| ZFHX3 | hsa-miR-532-5p, hsa-miR-211-5p | 2 |
| CAV1 | hsa-miR-670-3p | 1 |
| FGFR1 | hsa-miR-296-5p, hsa-miR-133b, hsa-miR-424-5p | 3 |
| NFIB | hsa-miR-21-5p, hsa-miR-93-5p, hsa-miR-129-5p, hsa-miR-130a-3p, hsa-miR-106b-5p, hsa-miR-130b-3p, hsa-miR-372-3p, hsa-miR-373-3p, hsa-miR-20b-5p, hsa-miR-491-5p, hsa-miR-520c-3p, hsa-miR-519d-3p, hsa-miR-3666, hsa-miR-92a-3p, hsa-miR-301a-3p, hsa-miR-302d-3p, hsa-miR-526b-3p, hsa-miR-520d-3p, hsa-miR-454-3p, hsa-miR-301b-3p, hsa-miR-20a-5p | 21 |
| LIMS1 | hsa-miR-129-5p, hsa-miR-340-5p | 2 |
| ALCAM | hsa-miR-148a-3p, hsa-miR-152-3p, hsa-miR-192-5p | 3 |
| RAD23B | hsa-miR-30d-5p, hsa-miR-424-5p, hsa-miR-6838-5p | 3 |
| FRMD3 | hsa-miR-342-3p | 1 |
| NOL4L | hsa-miR-15a-5p, hsa-miR-92a-3p, hsa-miR-195-5p, hsa-miR-92b-3p | 4 |
| RAB30 | hsa-miR-16-5p, hsa-miR-106b-5p, hsa-miR-20a-5p, hsa-miR-17-5p, hsa-miR-93-5p | 5 |
| DENND5B | hsa-miR-17-5p, hsa-miR-20a-5p, hsa-miR-93-5p, hsa-miR-106b-5p, hsa-miR-20b-5p | 5 |
| MAPK10 | hsa-miR-221-3p | 1 |
| ZNF532 | hsa-miR-106b-5p | 1 |
| FKBP1B | hsa-miR-34a-5p | 1 |
| TRAPPC10 | hsa-miR-519d-3p | 1 |
| TRMT13 | hsa-miR-7-5p | 1 |
| MSN | hsa-miR-192-5p, hsa-miR-215-5p, hsa-miR-338-3p | 3 |
| FSCN1 | hsa-miR-6088 | 1 |
| SNN | hsa-miR-92a-3p, hsa-miR-92b-3p | 2 |
| MMD | hsa-miR-205-5p | 1 |
| PDGFC | hsa-miR-29b-3p | 1 |
| DCBLD2 | hsa-miR-17-5p, hsa-miR-93-5p, hsa-miR-106a-5p, hsa-miR-139-5p, hsa-miR-130a-3p, hsa-miR-106b-5p, hsa-miR-130b-3p, hsa-miR-3666 | 8 |
| KLHDC8B | hsa-let-7d-5p, hsa-miR-4458 | 2 |

# Table S6. lncRNA-miRNA-mRNA ceRNA network

| **lncRNA** | **miRNA** | **mRNA** |
| --- | --- | --- |
| BX004987.4 | hsa-miR-139-5p | DCBLD2 |
| AC064834.2 | hsa-miR-139-5p | DCBLD2 |
| RP11-445P17.8 | hsa-miR-139-5p | DCBLD2 |
| RP3-399L15.3 | hsa-miR-139-5p | DCBLD2 |
| RP11-384C4.6 | hsa-miR-139-5p | DCBLD2 |
| BX571672.1 | hsa-miR-139-5p | DCBLD2 |
| AL583842.3 | hsa-miR-139-5p | DCBLD2 |
| RP4-569M23.2 | hsa-miR-139-5p | DCBLD2 |
| RP11-490N5.2 | hsa-miR-139-5p | DCBLD2 |
| RP11-65J3.1 | hsa-miR-139-5p | DCBLD2 |
| LINC00299 | hsa-miR-139-5p | DCBLD2 |
| AL592284.1 | hsa-miR-139-5p | DCBLD2 |
| LINC00211 | hsa-miR-139-5p | DCBLD2 |
| GCFC1-AS1 | hsa-miR-139-5p | DCBLD2 |
| RP11-511P7.2 | hsa-miR-139-5p | DCBLD2 |
| PVT1 | hsa-miR-139-5p | DCBLD2 |
| RP11-301H24.4 | hsa-miR-139-5p | DCBLD2 |
| LINC00534 | hsa-miR-139-5p | DCBLD2 |
| RP11-1033H12.1 | hsa-miR-139-5p | DCBLD2 |
| RP11-59H7.3 | hsa-miR-139-5p | DCBLD2 |
| AC083843.1 | hsa-miR-139-5p | DCBLD2 |
| AC137934.1 | hsa-miR-139-5p | DCBLD2 |
| BX004987.4 | hsa-miR-129-5p | NFIB |
| BX004987.4 | hsa-miR-129-5p | LIMS1 |
| AC064834.2 | hsa-miR-129-5p | NFIB |
| AC064834.2 | hsa-miR-129-5p | LIMS1 |
| AC009480.3 | hsa-miR-129-5p | NFIB |
| AC009480.3 | hsa-miR-129-5p | LIMS1 |
| RP1-18D14.4 | hsa-miR-129-5p | NFIB |
| RP1-18D14.4 | hsa-miR-129-5p | LIMS1 |
| AC093818.1 | hsa-miR-129-5p | NFIB |
| AC093818.1 | hsa-miR-129-5p | LIMS1 |
| RP1-18D14.7 | hsa-miR-129-5p | NFIB |
| RP1-18D14.7 | hsa-miR-129-5p | LIMS1 |
| RP11-162D16.2 | hsa-miR-129-5p | NFIB |
| RP11-162D16.2 | hsa-miR-129-5p | LIMS1 |
| AC017076.4 | hsa-miR-129-5p | NFIB |
| AC017076.4 | hsa-miR-129-5p | LIMS1 |
| RP11-216B9.6 | hsa-miR-129-5p | NFIB |
| RP11-216B9.6 | hsa-miR-129-5p | LIMS1 |
| RP3-399L15.3 | hsa-miR-129-5p | NFIB |
| RP3-399L15.3 | hsa-miR-129-5p | LIMS1 |
| RP11-175B12.2 | hsa-miR-129-5p | NFIB |
| RP11-175B12.2 | hsa-miR-129-5p | LIMS1 |
| RP11-384C4.6 | hsa-miR-129-5p | NFIB |
| RP11-384C4.6 | hsa-miR-129-5p | LIMS1 |
| AL583842.3 | hsa-miR-129-5p | NFIB |
| AL583842.3 | hsa-miR-129-5p | LIMS1 |
| RP5-1073O3.2 | hsa-miR-129-5p | NFIB |
| RP5-1073O3.2 | hsa-miR-129-5p | LIMS1 |
| FARP1-AS1 | hsa-miR-129-5p | NFIB |
| FARP1-AS1 | hsa-miR-129-5p | LIMS1 |
| AC096579.7 | hsa-miR-129-5p | NFIB |
| AC096579.7 | hsa-miR-129-5p | LIMS1 |
| RP11-490N5.2 | hsa-miR-129-5p | NFIB |
| RP11-490N5.2 | hsa-miR-129-5p | LIMS1 |
| AC002480.3 | hsa-miR-129-5p | NFIB |
| AC002480.3 | hsa-miR-129-5p | LIMS1 |
| AC019186.1 | hsa-miR-129-5p | NFIB |
| AC019186.1 | hsa-miR-129-5p | LIMS1 |
| RP4-756G23.5 | hsa-miR-129-5p | NFIB |
| RP4-756G23.5 | hsa-miR-129-5p | LIMS1 |
| AL592284.1 | hsa-miR-129-5p | NFIB |
| AL592284.1 | hsa-miR-129-5p | LIMS1 |
| GCFC1-AS1 | hsa-miR-129-5p | NFIB |
| GCFC1-AS1 | hsa-miR-129-5p | LIMS1 |
| RP11-159H10.3 | hsa-miR-129-5p | NFIB |
| RP11-159H10.3 | hsa-miR-129-5p | LIMS1 |
| CTC-463N11.3 | hsa-miR-129-5p | NFIB |
| CTC-463N11.3 | hsa-miR-129-5p | LIMS1 |
| RP11-730K11.1 | hsa-miR-129-5p | NFIB |
| RP11-730K11.1 | hsa-miR-129-5p | LIMS1 |
| CTC-454M9.1 | hsa-miR-129-5p | NFIB |
| CTC-454M9.1 | hsa-miR-129-5p | LIMS1 |
| RP11-438E5.1 | hsa-miR-129-5p | NFIB |
| RP11-438E5.1 | hsa-miR-129-5p | LIMS1 |
| U66061.31 | hsa-miR-129-5p | NFIB |
| U66061.31 | hsa-miR-129-5p | LIMS1 |
| RP11-689K5.3 | hsa-miR-129-5p | NFIB |
| RP11-689K5.3 | hsa-miR-129-5p | LIMS1 |
| RP11-673E1.1 | hsa-miR-129-5p | NFIB |
| RP11-673E1.1 | hsa-miR-129-5p | LIMS1 |
| RP11-45K10.2 | hsa-miR-129-5p | NFIB |
| RP11-45K10.2 | hsa-miR-129-5p | LIMS1 |
| CTD-2336O2.1 | hsa-miR-129-5p | NFIB |
| CTD-2336O2.1 | hsa-miR-129-5p | LIMS1 |
| RP11-677I18.3 | hsa-miR-129-5p | NFIB |
| RP11-677I18.3 | hsa-miR-129-5p | LIMS1 |
| RP3-405J10.3 | hsa-miR-129-5p | NFIB |
| RP3-405J10.3 | hsa-miR-129-5p | LIMS1 |
| RP11-616L12.1 | hsa-miR-129-5p | NFIB |
| RP11-616L12.1 | hsa-miR-129-5p | LIMS1 |
| RP11-70F11.6 | hsa-miR-129-5p | NFIB |
| RP11-70F11.6 | hsa-miR-129-5p | LIMS1 |
| RP11-701B16.2 | hsa-miR-129-5p | NFIB |
| RP11-701B16.2 | hsa-miR-129-5p | LIMS1 |
| LINC00520 | hsa-miR-129-5p | NFIB |
| LINC00520 | hsa-miR-129-5p | LIMS1 |
| RP11-300J18.3 | hsa-miR-129-5p | NFIB |
| RP11-300J18.3 | hsa-miR-129-5p | LIMS1 |
| RP11-317N8.5 | hsa-miR-129-5p | NFIB |
| RP11-317N8.5 | hsa-miR-129-5p | LIMS1 |
| RP11-930O11.2 | hsa-miR-129-5p | NFIB |
| RP11-930O11.2 | hsa-miR-129-5p | LIMS1 |
| RP11-111A22.1 | hsa-miR-129-5p | NFIB |
| RP11-111A22.1 | hsa-miR-129-5p | LIMS1 |
| RP11-59H7.3 | hsa-miR-129-5p | NFIB |
| RP11-59H7.3 | hsa-miR-129-5p | LIMS1 |
| AC083843.1 | hsa-miR-129-5p | NFIB |
| AC083843.1 | hsa-miR-129-5p | LIMS1 |
| RP11-366L5.1 | hsa-miR-129-5p | NFIB |
| RP11-366L5.1 | hsa-miR-129-5p | LIMS1 |
| RP11-104N10.1 | hsa-miR-129-5p | NFIB |
| RP11-104N10.1 | hsa-miR-129-5p | LIMS1 |

# Table S7. Clinical characteristics of study participants in the hypertensive and control groups of qRT-PCR

|  | Normal (*n* = 15) | Hypertension (*n* = 15) | *P*-value |
| --- | --- | --- | --- |
| Age (years) | 42.00±2.59 | 41.60±1.99 | 0.607^†^ |
| Gender (Male:Female) | 6:9 | 5:10 | >0.9999^‡^ |
| Smoking (Y:N) | 3:12 | 5:10 | 0.682^‡^ |
| Drinking (Y:N) | 4:11 | 6:9 | 0,700^‡^ |
| BMI (kg/m^2^) | 24.23±3.31 | 24.28±3.60 | 0.970^†^ |
| FBG (mmol/L) | 5.30±0.0.46 | 5.37±0.40 | 0.628^†^ |
| TG (mmol/L) | 1.20±0.47 | 1.43±0.35 | 0.132^†^ |
| TC (mmol/L) | 4.52±0.49 | 4.50±0.59 | 0.934^†^ |
| HDL-C (mmol/L) | 1.48±0.16 | 1.45±0.14 | 0.638^†^ |
| LDL-C (mmol/L) | 2.46±0.56 | 2.44±0.46 | 0.930^†^ |
| SBP (mm Hg) | 77.00±8.86 | 97.93±6.45^*^ | *P*<0.05^†^ |
| DBP (mm Hg) | 118.27±9.82 | 161.33±18.69^*^ | *P*<0.05^†^ |

†, Statistical testing by independent-samples t test. ‡, Statistical testing by χ^2^ test.

*^*^P* < 0.05 *vs* normal group.
